# Supplementary figures and images for: Plasmodium falciparum impairs Ang-1 secretion by pericytes in a 3D brain microvessel model (part 1 of 4)
Source: EMBO Mol Med. 2025 Oct 16;17(11):3110–38. doi: 10.1038/s44321-025-00319-y (PMC12603187; doi:10.1038/s44321-025-00319-y)

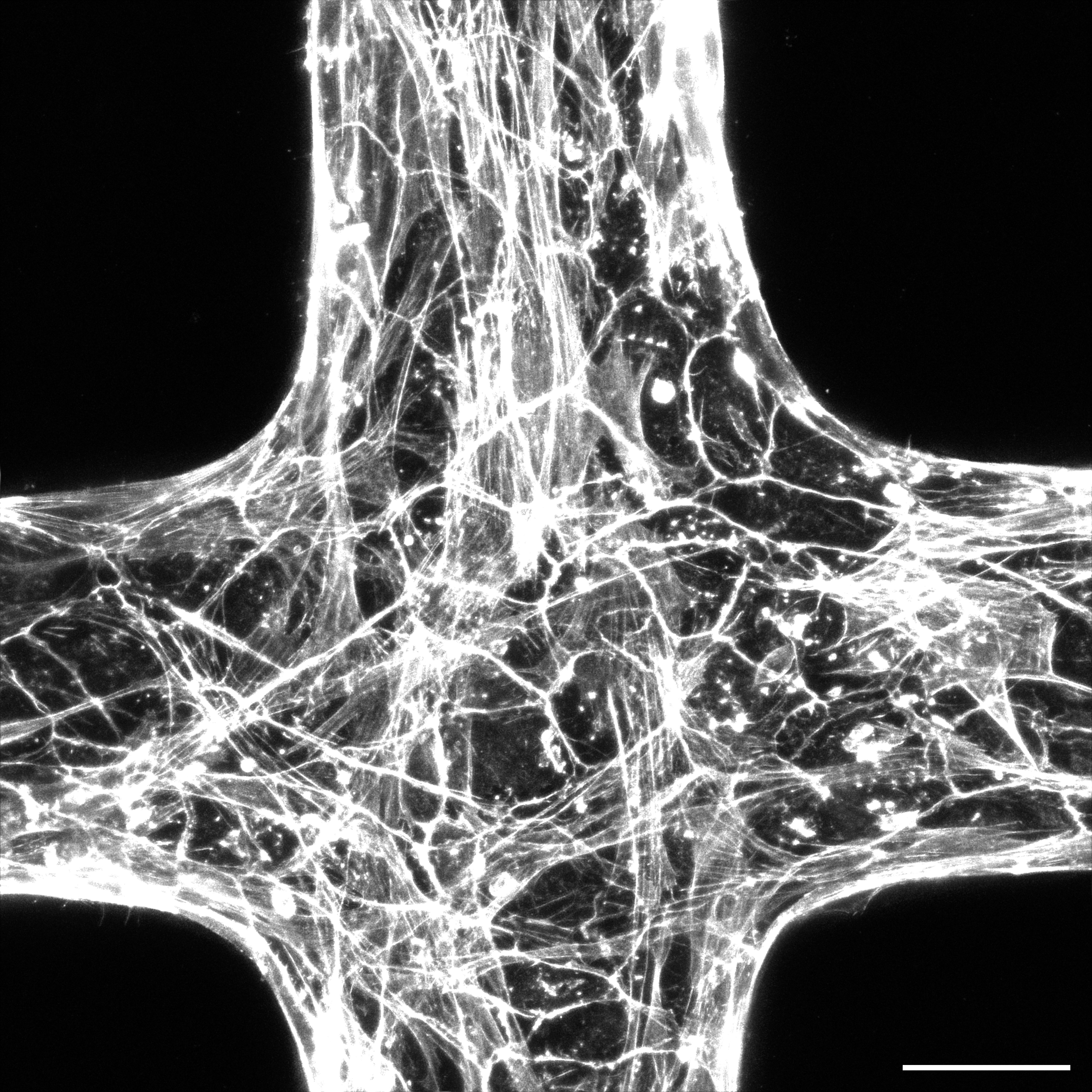

Supplement: Supplementary file 2 — Source data Fig. 1 [file 44321_2025_319_MOESM2_ESM.zip › Figure 1/Panel D/Figure 1 MAX_PC48_6 aSMA Top.jpg]

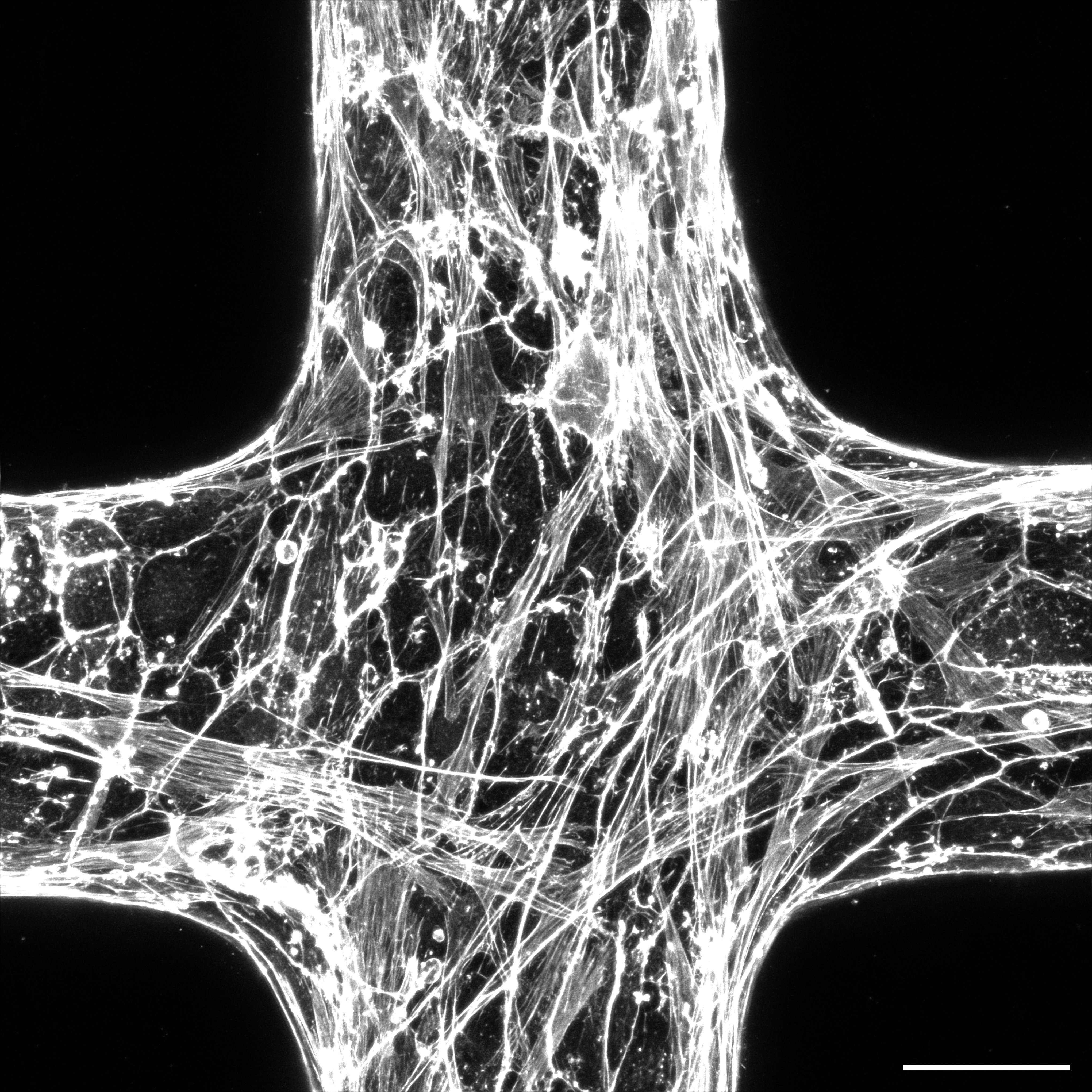

Supplement: Supplementary file 2 — Source data Fig. 1 [file 44321_2025_319_MOESM2_ESM.zip › Figure 1/Panel D/Figure 1_MAX_PC48_6 aSMA Bottom.jpg]

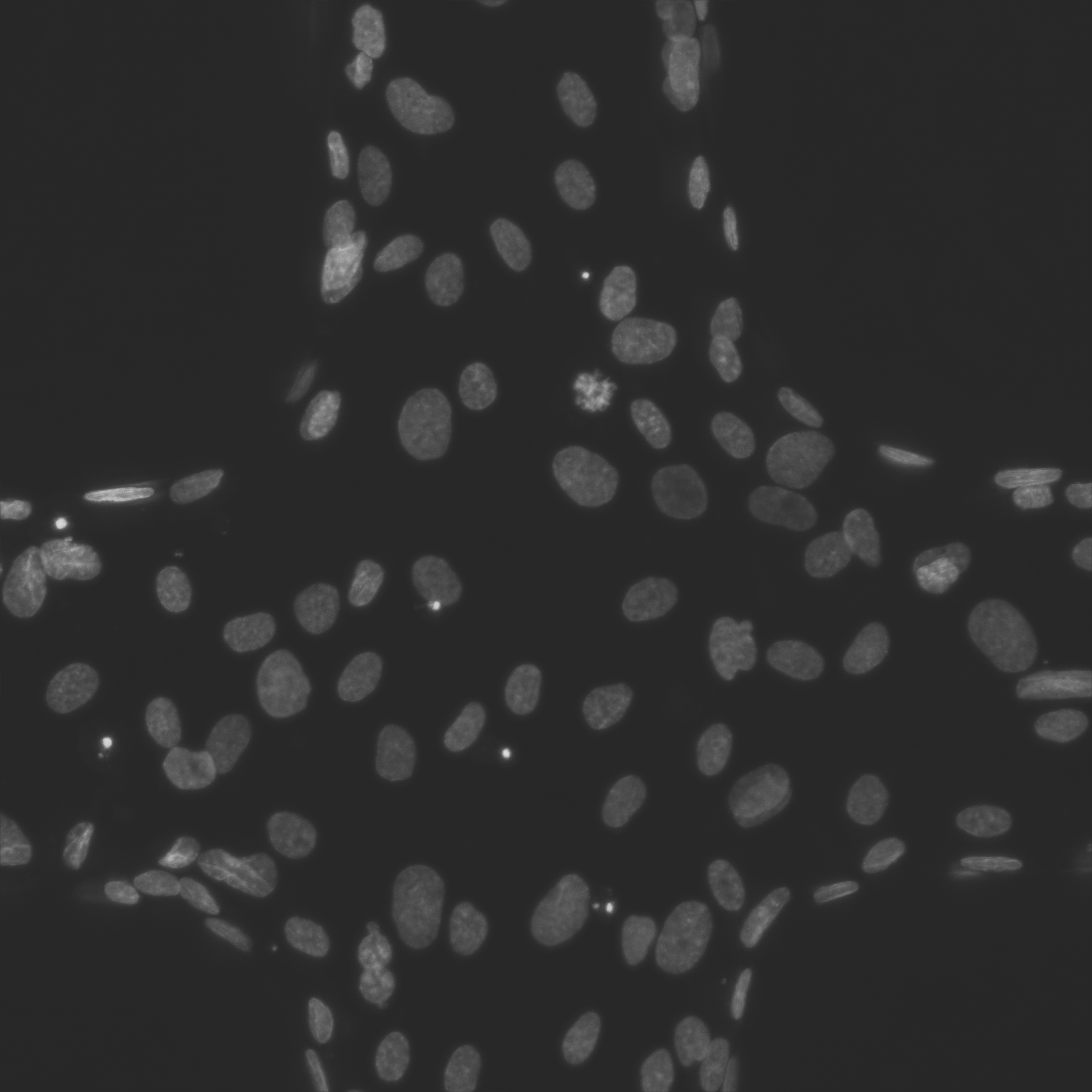

Supplement: Supplementary file 2 — Source data Fig. 1 [file 44321_2025_319_MOESM2_ESM.zip › Figure 1/Panel D/Figure 1_MAX_PC48_6 DAPI Bottom.tif]

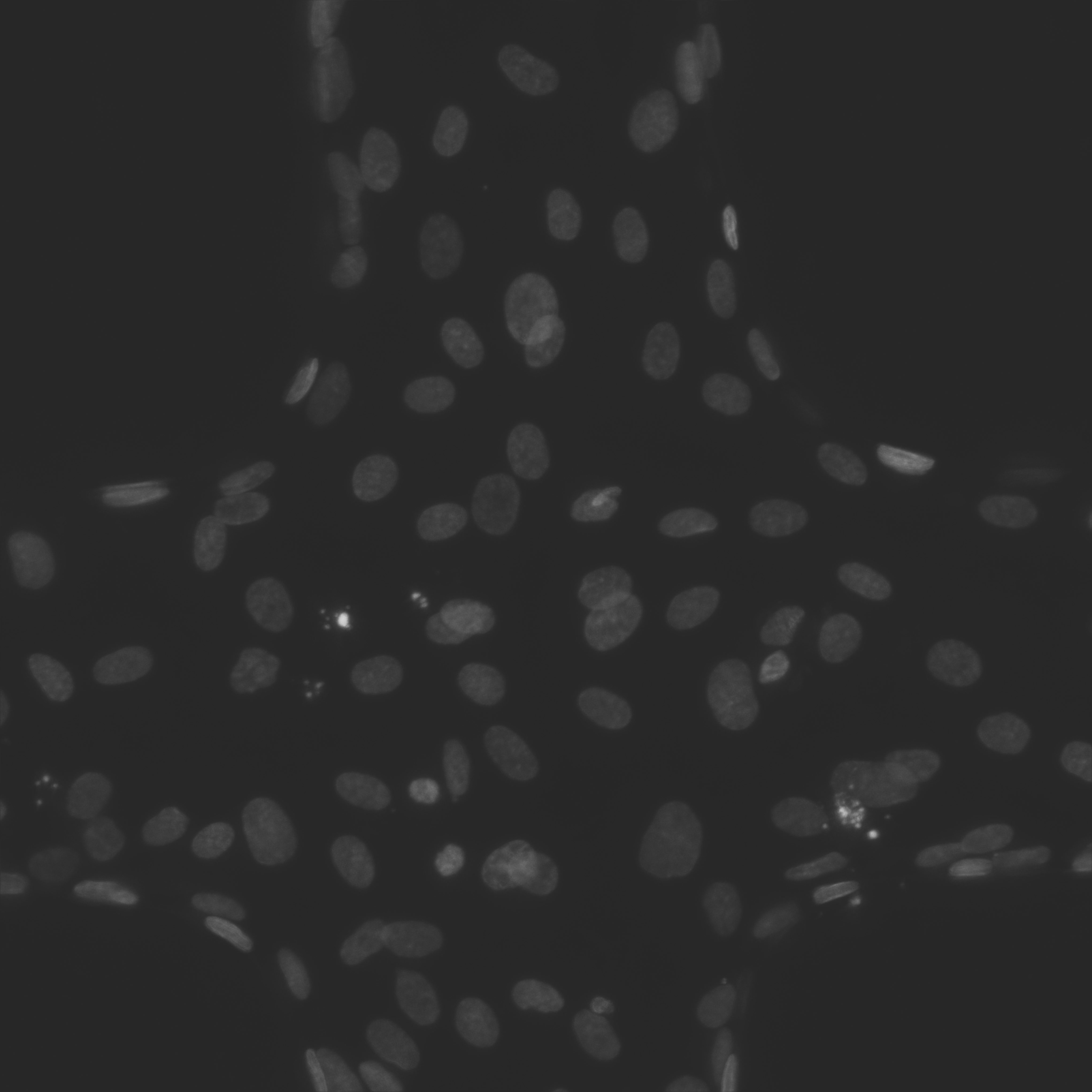

Supplement: Supplementary file 2 — Source data Fig. 1 [file 44321_2025_319_MOESM2_ESM.zip › Figure 1/Panel D/Figure 1_MAX_PC48_6 DAPI Top.tif]

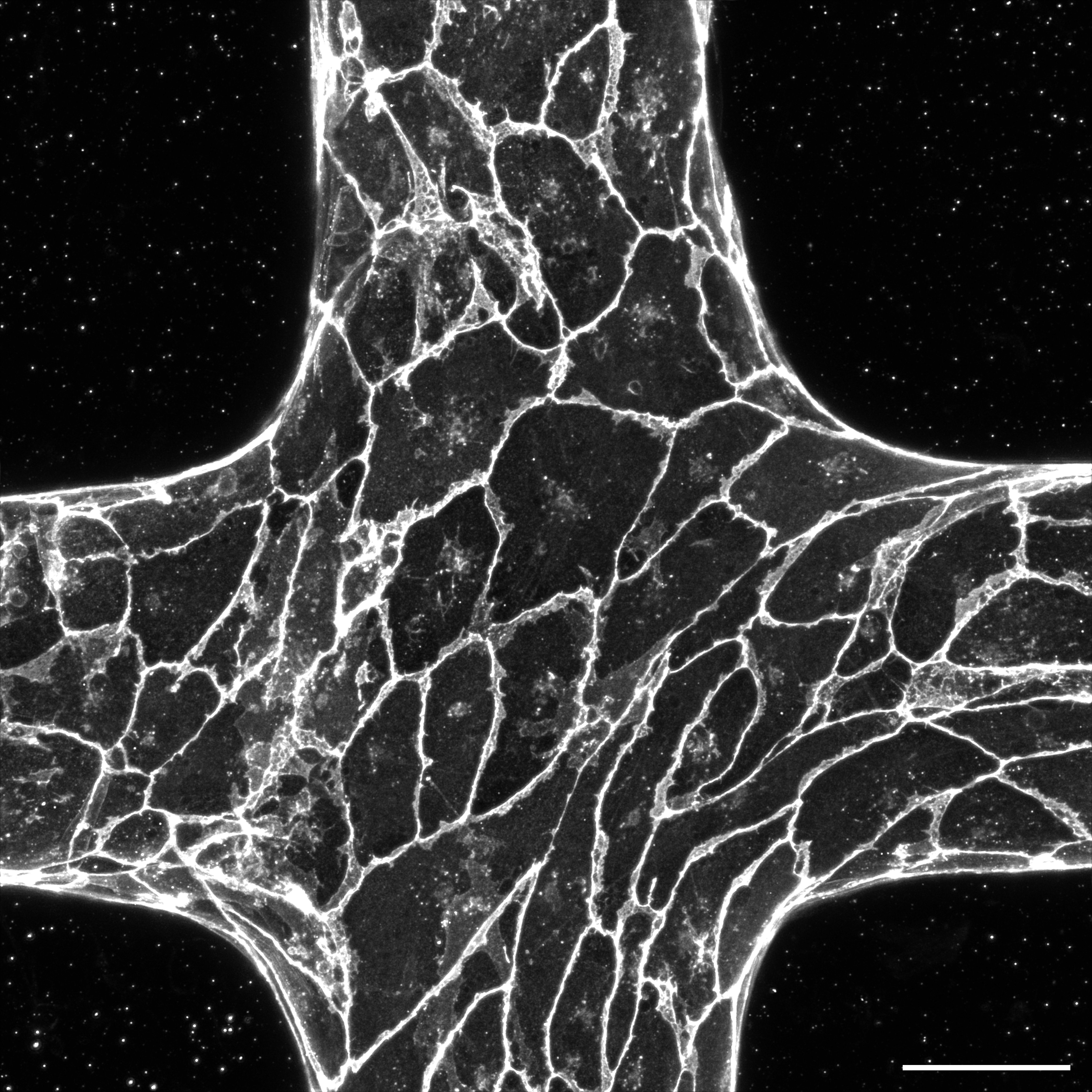

Supplement: Supplementary file 2 — Source data Fig. 1 [file 44321_2025_319_MOESM2_ESM.zip › Figure 1/Panel D/Figure1_MAX_PC48_6 VECAD Bottom.jpg]

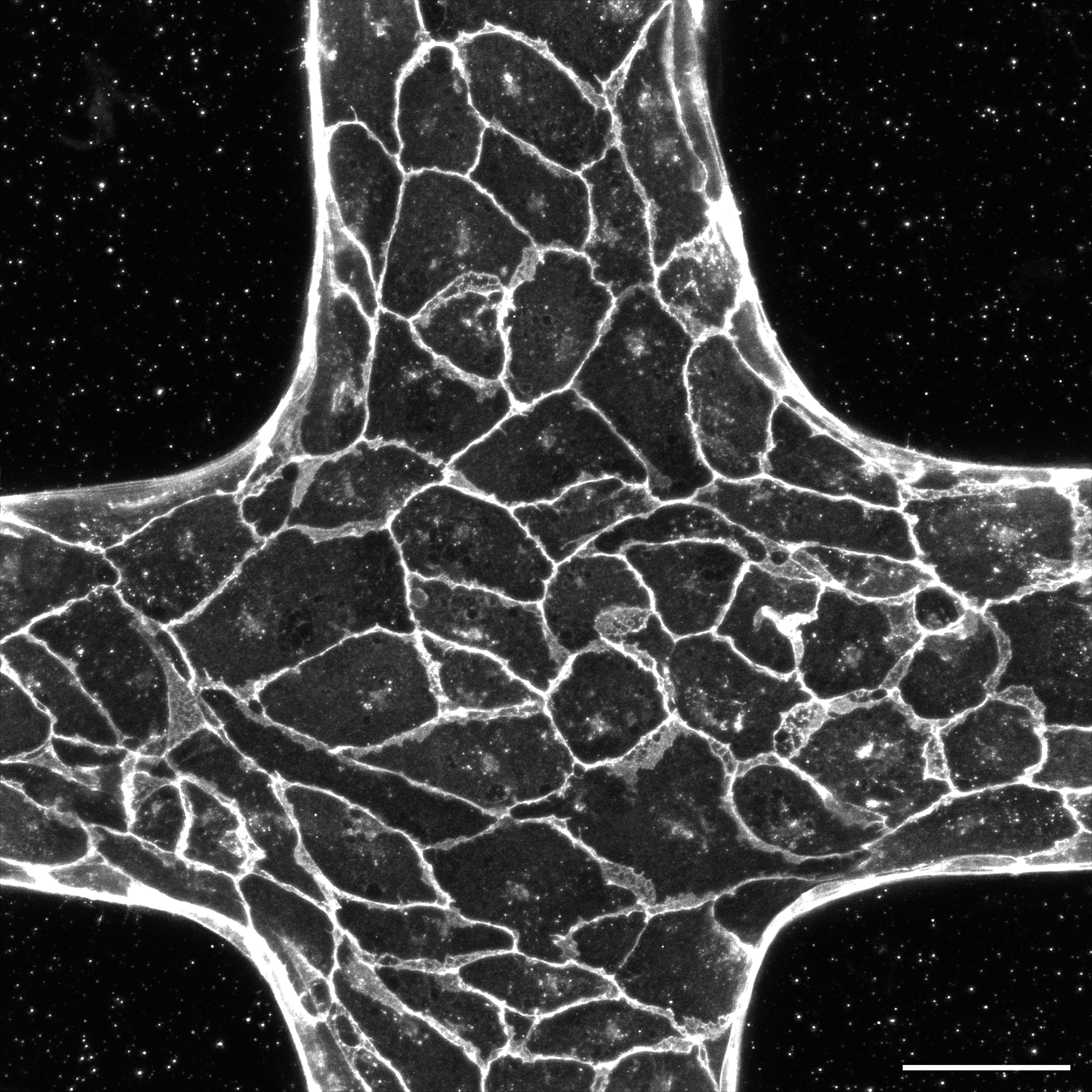

Supplement: Supplementary file 2 — Source data Fig. 1 [file 44321_2025_319_MOESM2_ESM.zip › Figure 1/Panel D/Figure1_MAX_PC48_6 VECAD Top.jpg]

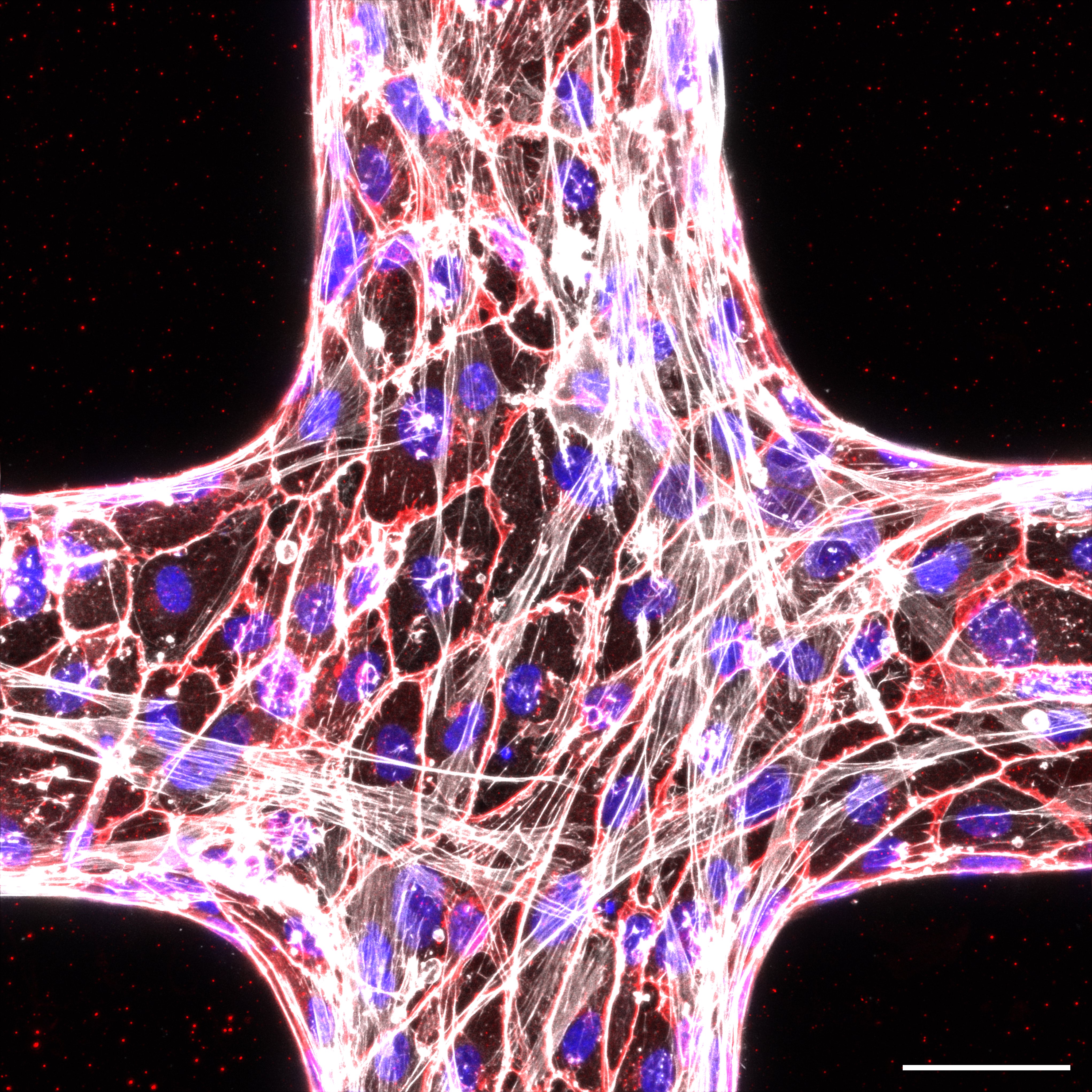

Supplement: Supplementary file 2 — Source data Fig. 1 [file 44321_2025_319_MOESM2_ESM.zip › Figure 1/Panel D/Figure1_MAX_PC48_6_Bottom Composite.jpg]

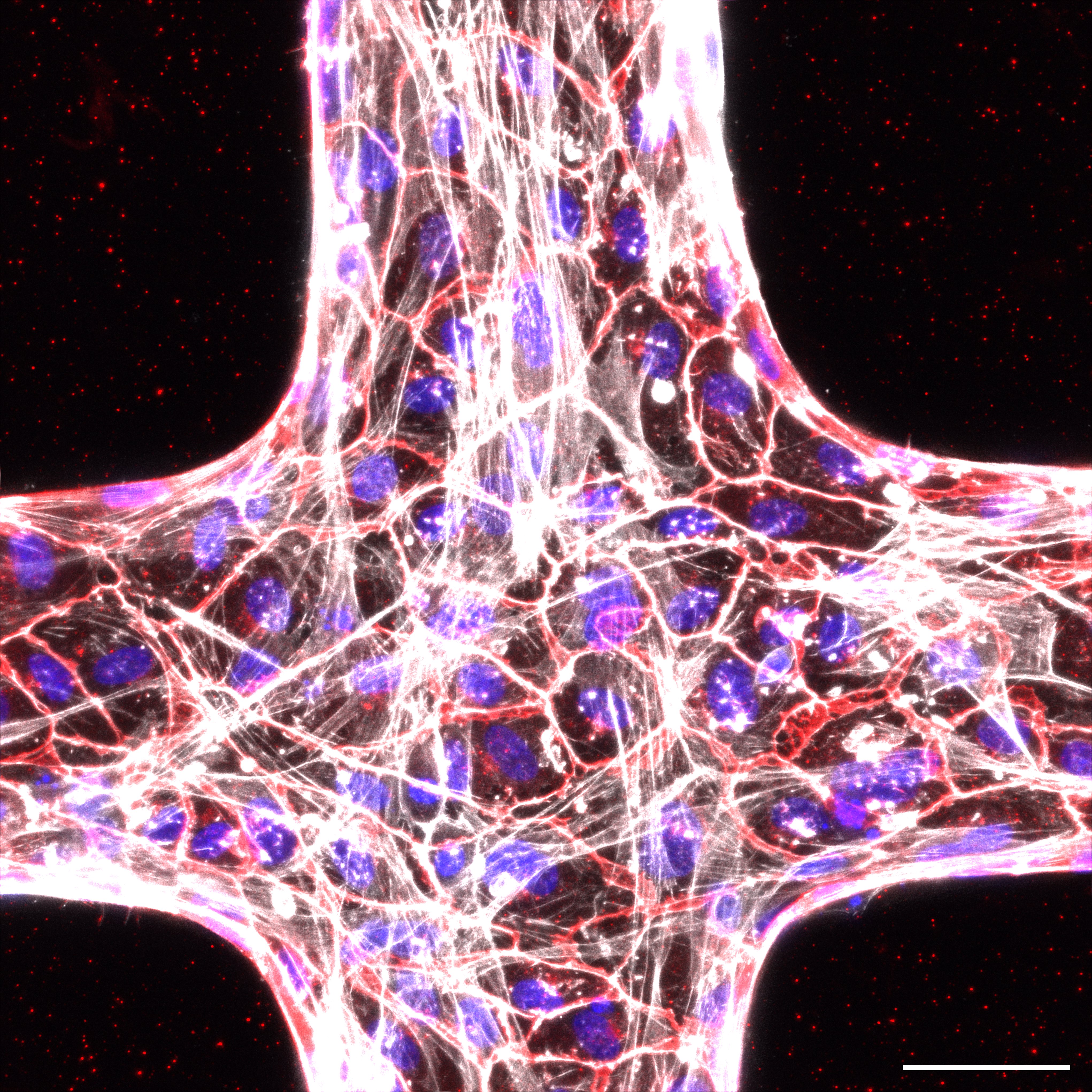

Supplement: Supplementary file 2 — Source data Fig. 1 [file 44321_2025_319_MOESM2_ESM.zip › Figure 1/Panel D/Figure1_MAX_PC48_6_Top_Composite.jpg]

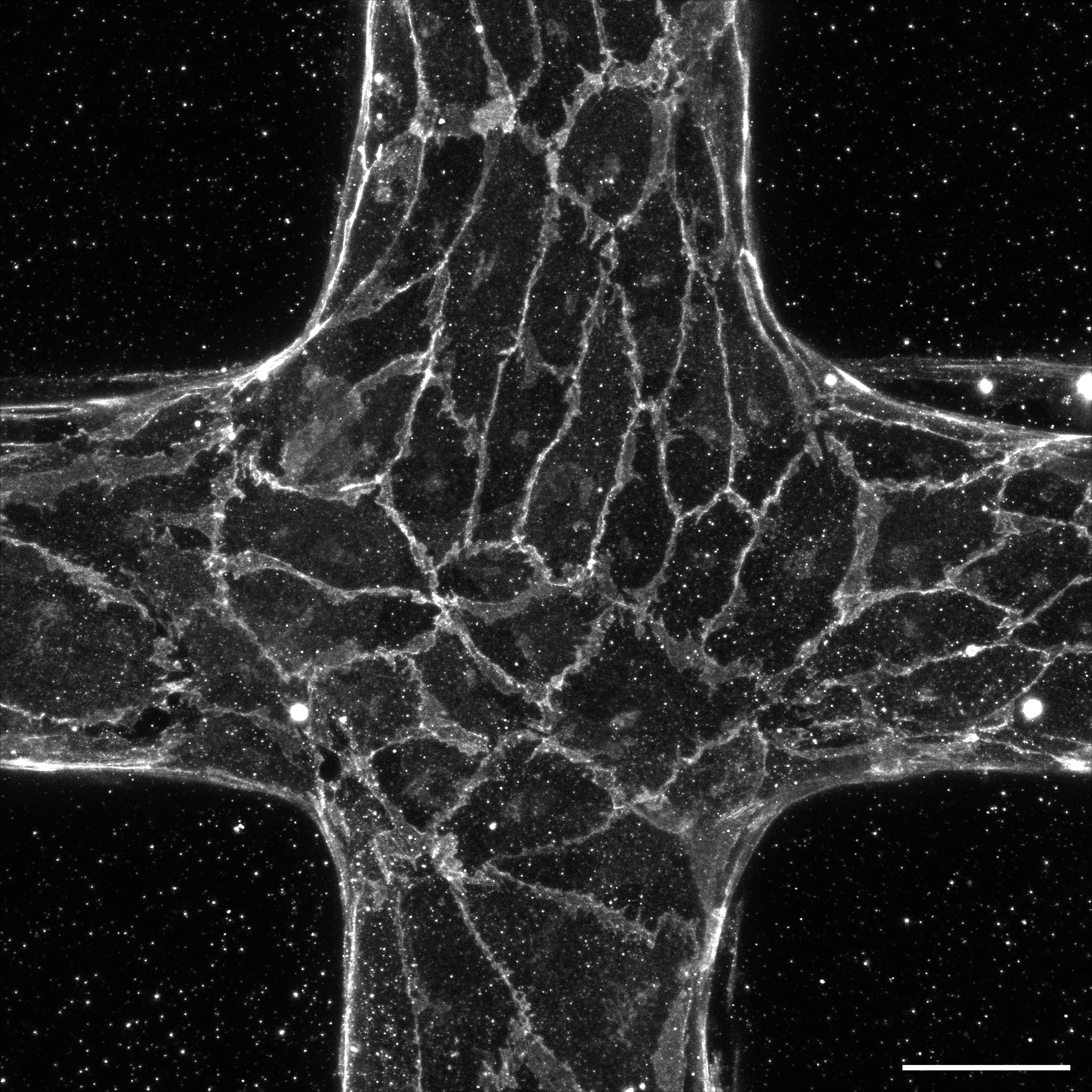

Supplement: Supplementary file 2 — Source data Fig. 1 [file 44321_2025_319_MOESM2_ESM.zip › Figure 1/Panel E/MAX_PC59_5_1_Device_media_only_Bcatenin.jpg]

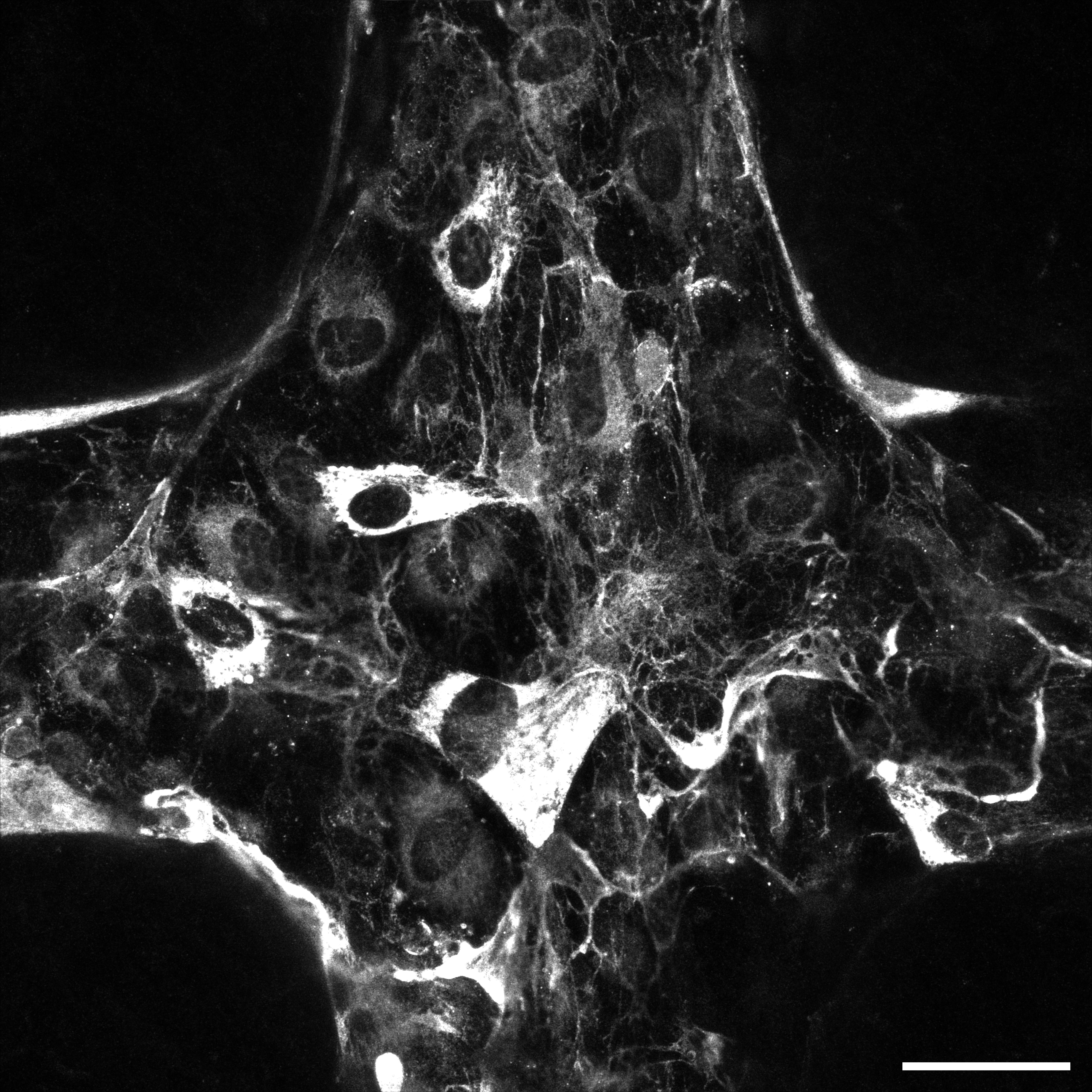

Supplement: Supplementary file 2 — Source data Fig. 1 [file 44321_2025_319_MOESM2_ESM.zip › Figure 1/Panel E/MAX_PC59_5_1_Device_media_only_laminin.jpg]

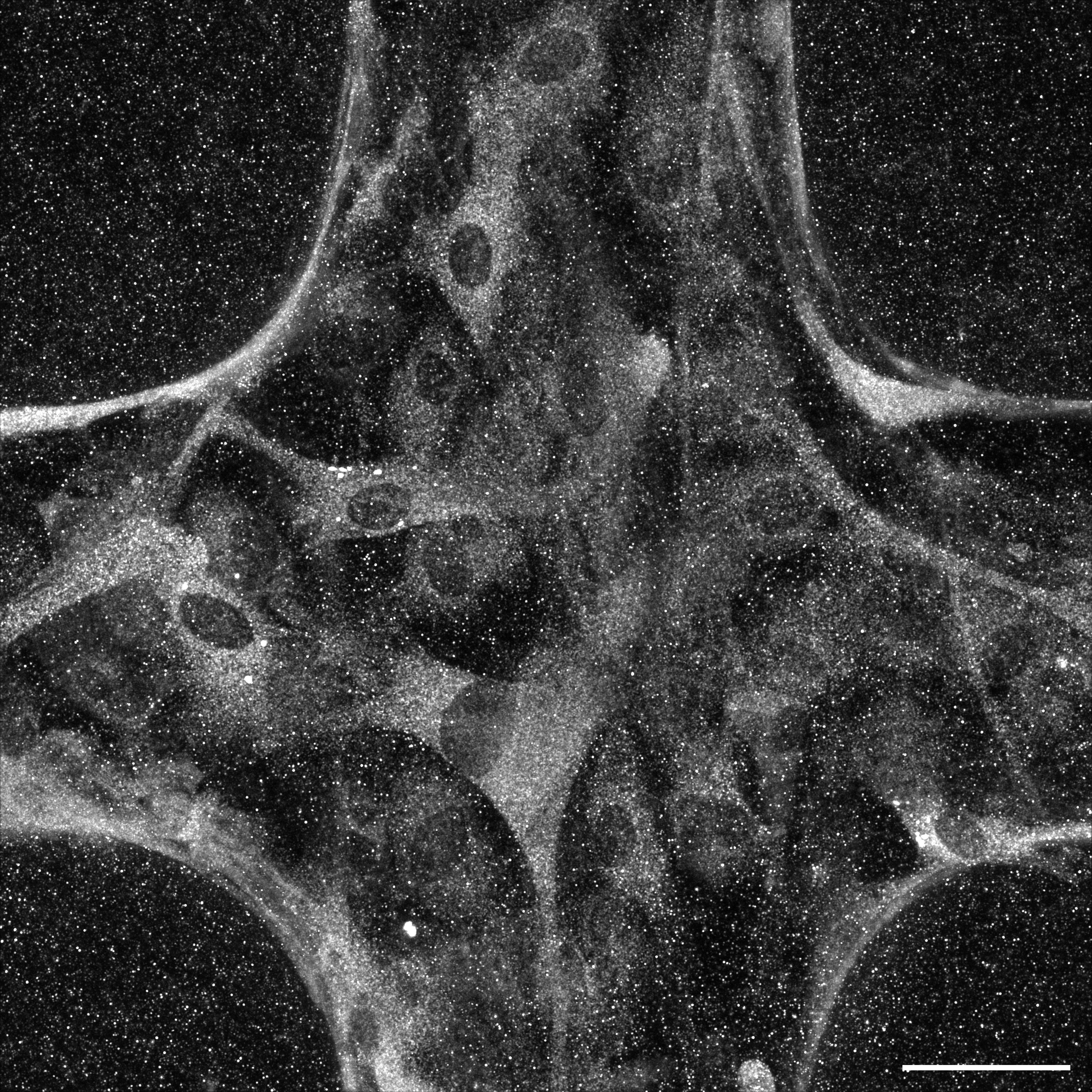

Supplement: Supplementary file 2 — Source data Fig. 1 [file 44321_2025_319_MOESM2_ESM.zip › Figure 1/Panel E/MAX_PC59_5_1_Device_media_only_PDGFRB.jpg]

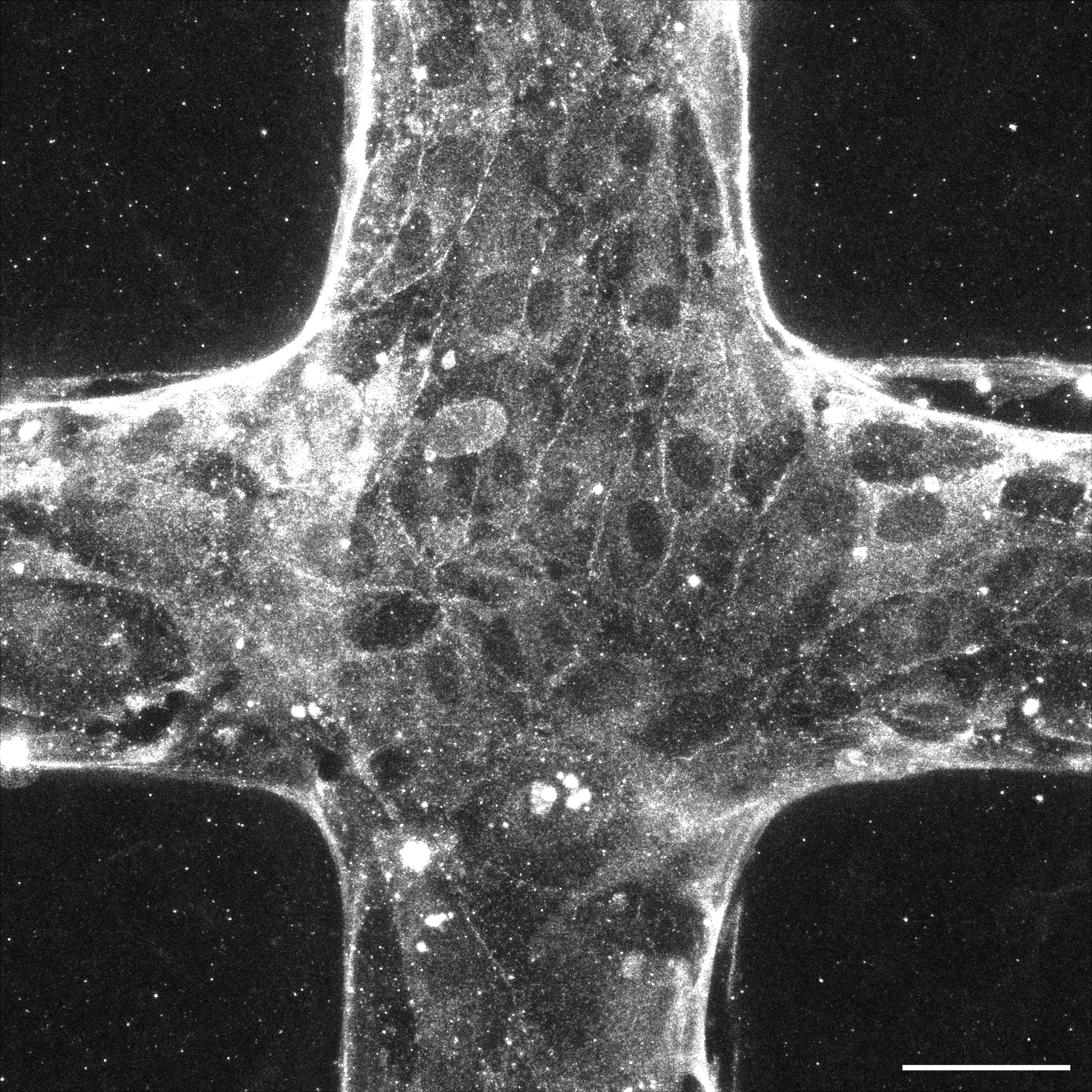

Supplement: Supplementary file 2 — Source data Fig. 1 [file 44321_2025_319_MOESM2_ESM.zip › Figure 1/Panel E/MAX_PC59_5_1_Device_media_only_ZO_1.jpg]

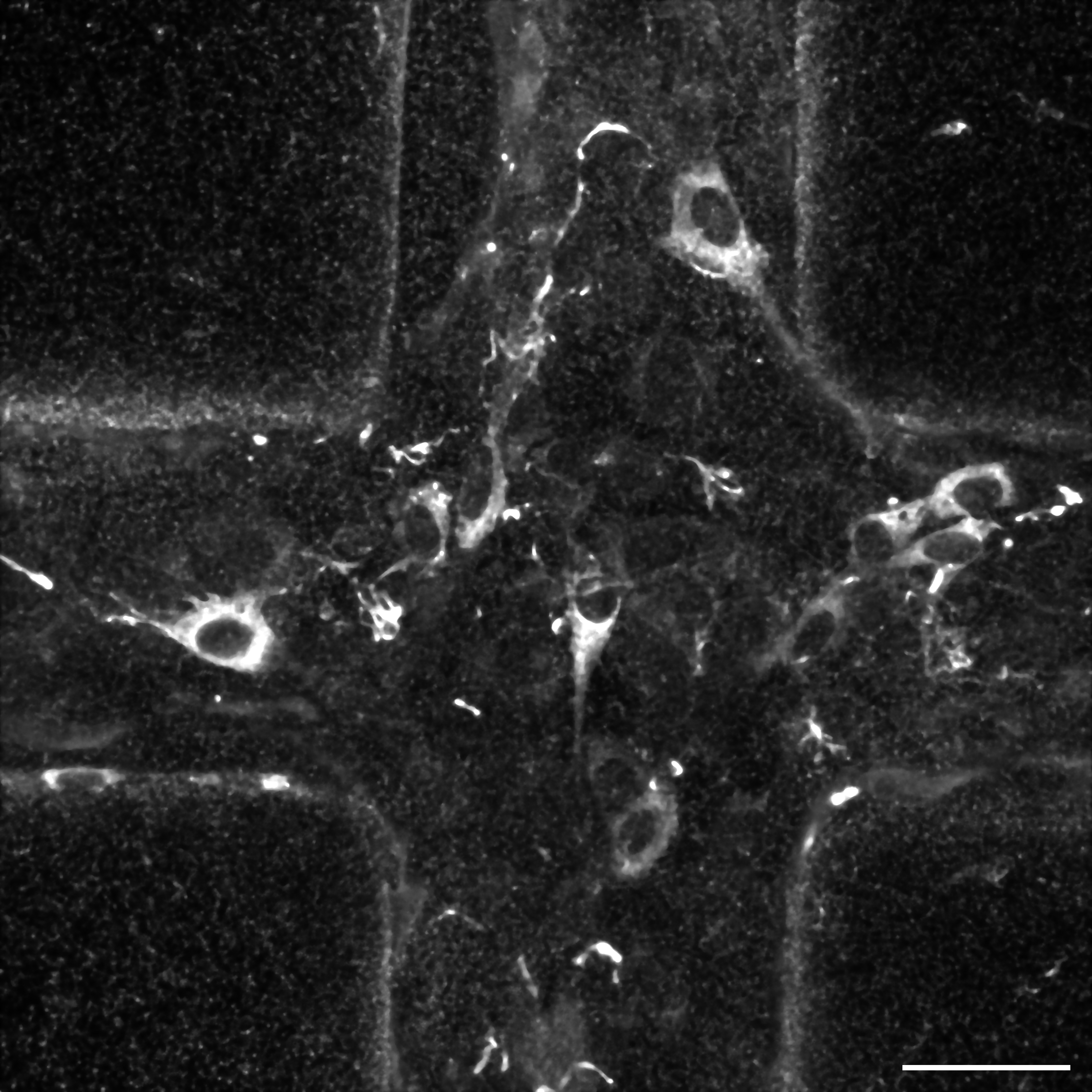

Supplement: Supplementary file 2 — Source data Fig. 1 [file 44321_2025_319_MOESM2_ESM.zip › Figure 1/Panel E/MAX_PC74_1_5_1_Collagen4.tif]

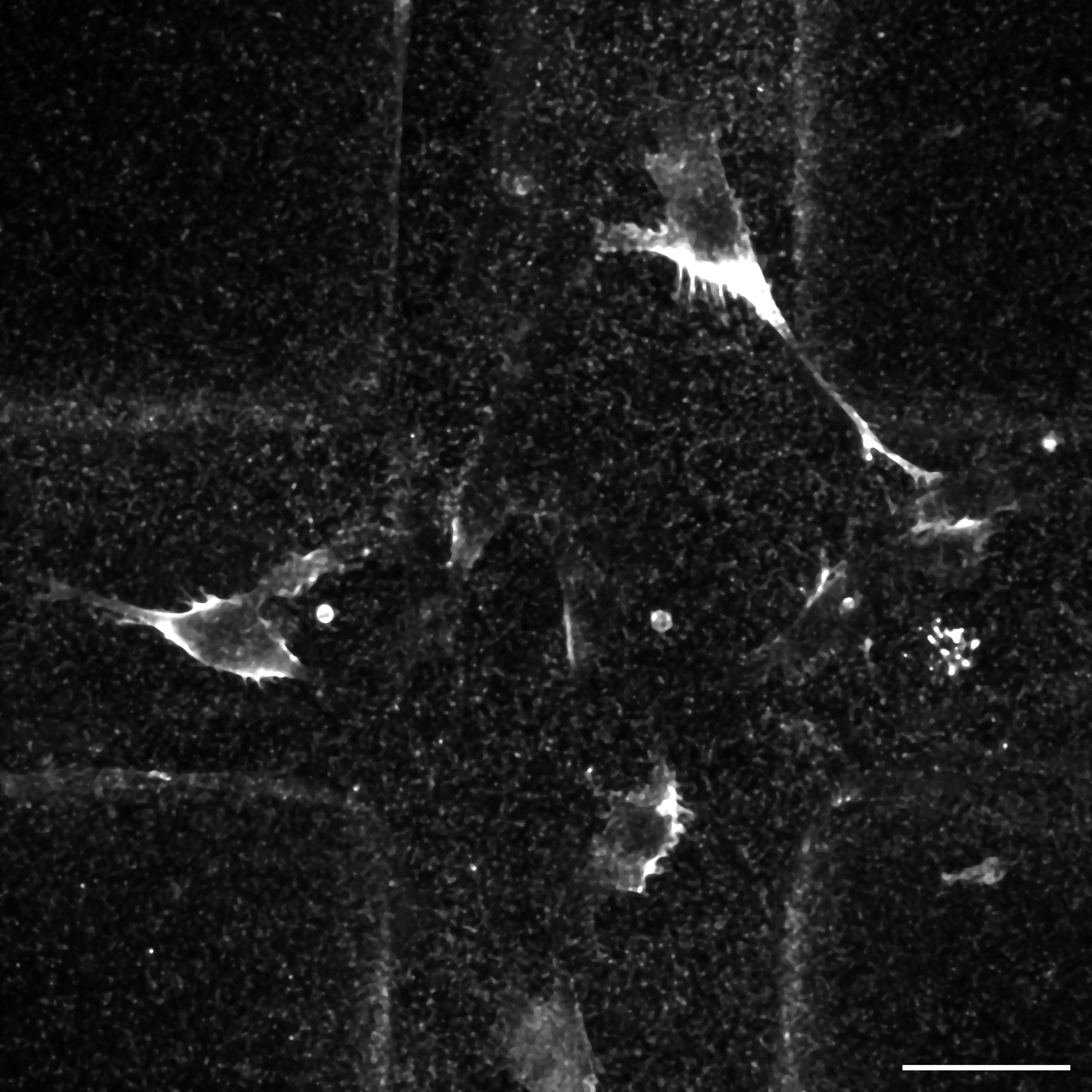

Supplement: Supplementary file 2 — Source data Fig. 1 [file 44321_2025_319_MOESM2_ESM.zip › Figure 1/Panel E/MAX_PC74_1_5_1NG2.tif]

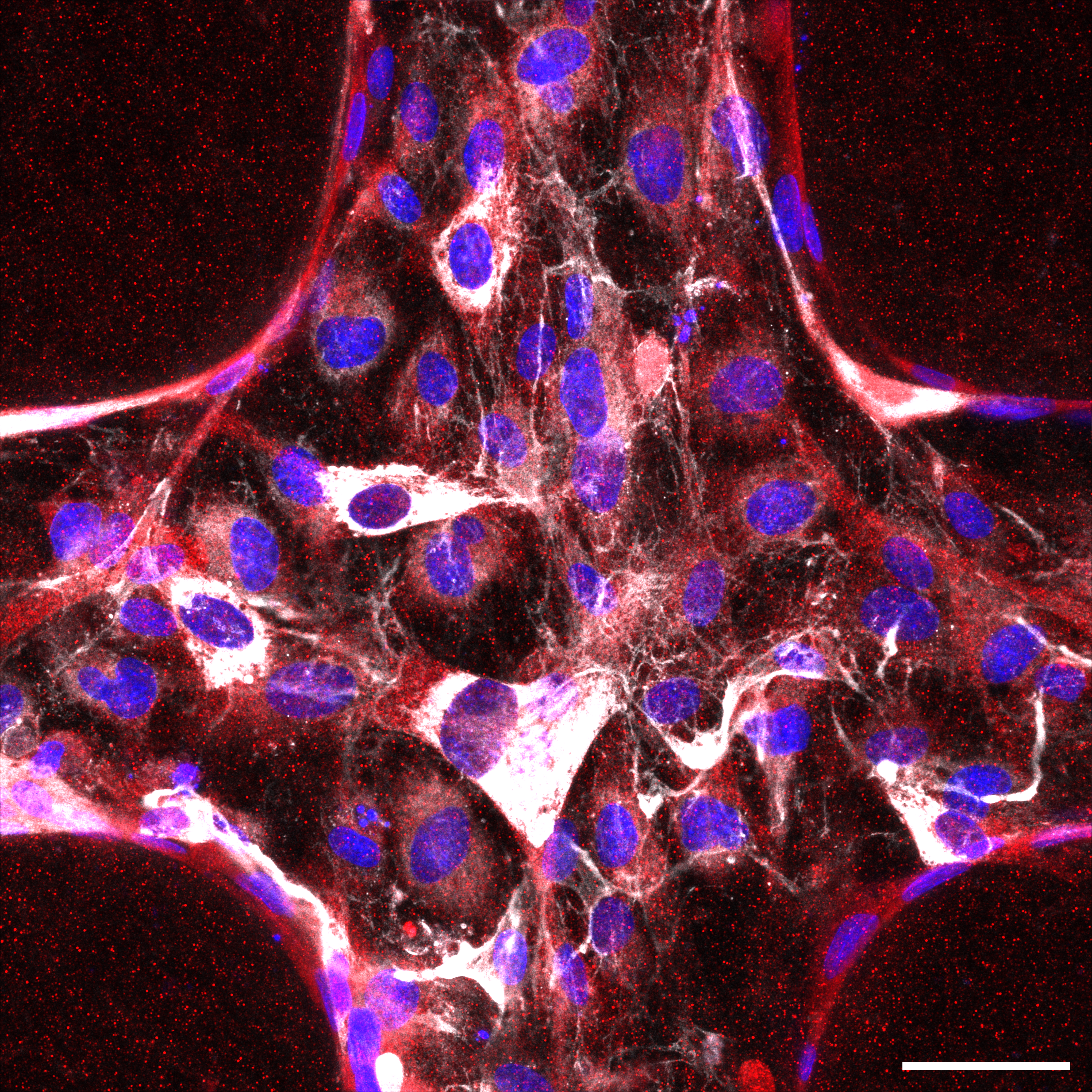

Supplement: Supplementary file 2 — Source data Fig. 1 [file 44321_2025_319_MOESM2_ESM.zip › Figure 1/Panel E/Merge_PDGFRB_Laminin_DAPI_Merge.tif]

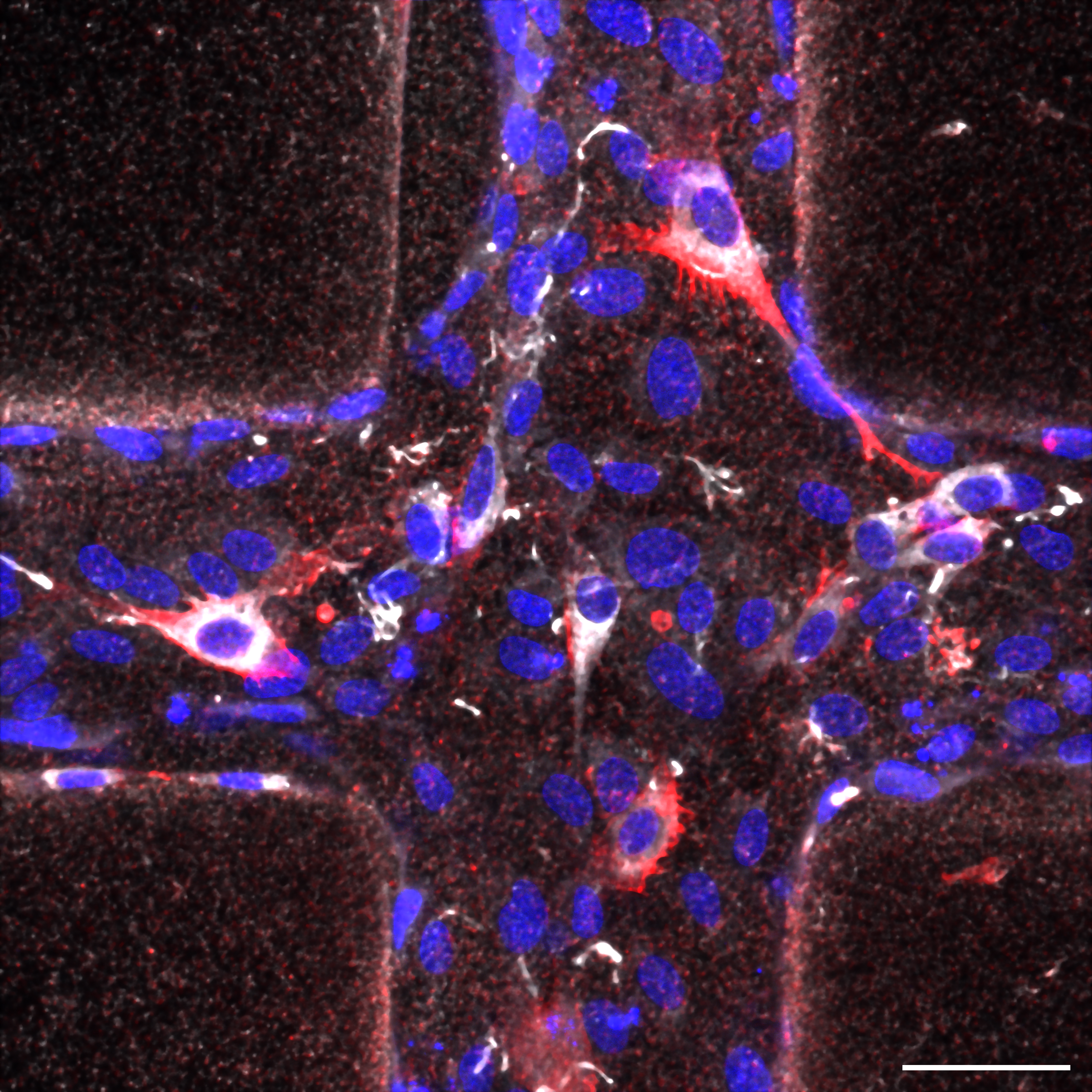

Supplement: Supplementary file 2 — Source data Fig. 1 [file 44321_2025_319_MOESM2_ESM.zip › Figure 1/Panel E/NG2_Collagen4_DAPI_Merge.tif]

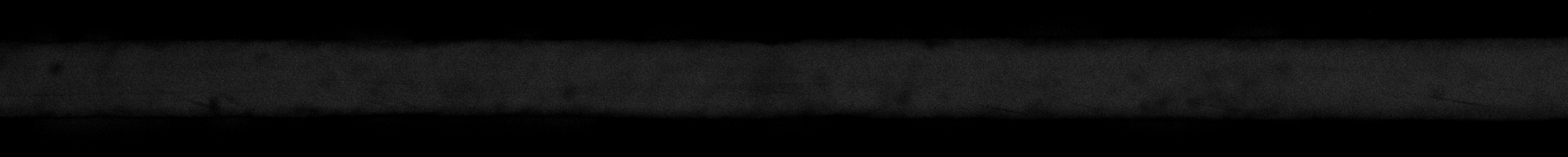

Supplement: Supplementary file 2 — Source data Fig. 1 [file 44321_2025_319_MOESM2_ESM.zip › Figure 1/Panel F/5_1 HBMEC_HBVP devices/PC62_3_Before_0min.tif]

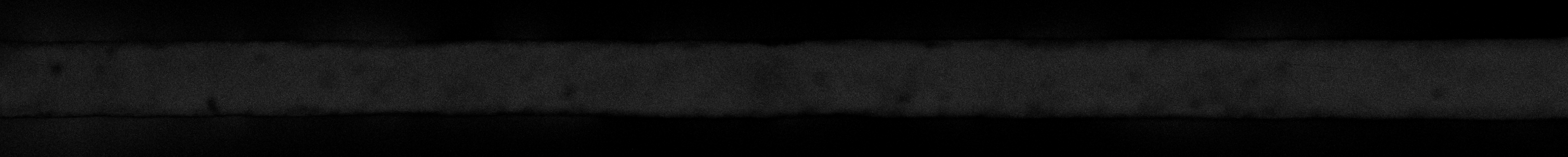

Supplement: Supplementary file 2 — Source data Fig. 1 [file 44321_2025_319_MOESM2_ESM.zip › Figure 1/Panel F/5_1 HBMEC_HBVP devices/PC62_3_Before_5min.tif]

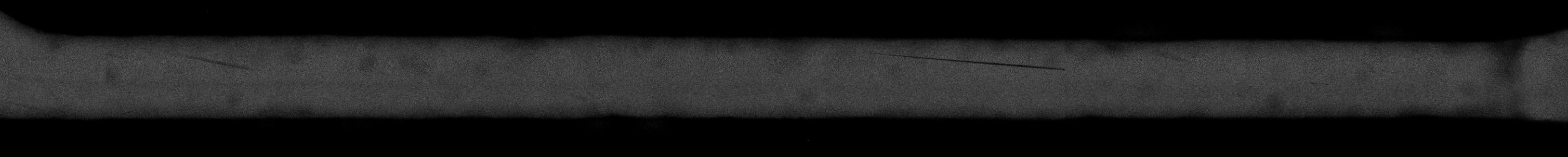

Supplement: Supplementary file 2 — Source data Fig. 1 [file 44321_2025_319_MOESM2_ESM.zip › Figure 1/Panel F/5_1 HBMEC_HBVP devices/PC62_4_Before_Time0.tif]

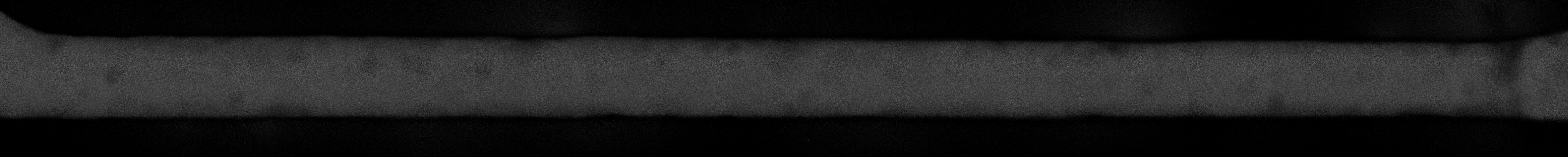

Supplement: Supplementary file 2 — Source data Fig. 1 [file 44321_2025_319_MOESM2_ESM.zip › Figure 1/Panel F/5_1 HBMEC_HBVP devices/PC62_4_Before_Time5.tif]

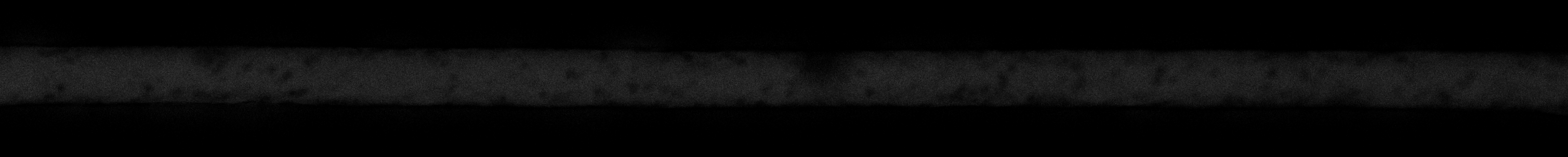

Supplement: Supplementary file 2 — Source data Fig. 1 [file 44321_2025_319_MOESM2_ESM.zip › Figure 1/Panel F/5_1 HBMEC_HBVP devices/PC63_10_Before_Time0.tif]

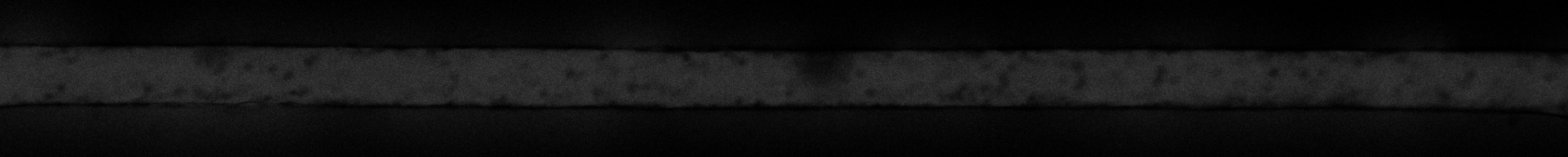

Supplement: Supplementary file 2 — Source data Fig. 1 [file 44321_2025_319_MOESM2_ESM.zip › Figure 1/Panel F/5_1 HBMEC_HBVP devices/PC63_10_Before_Time5minutes.tif]

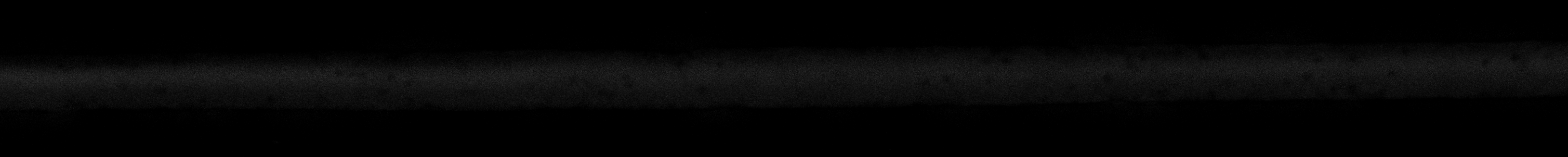

Supplement: Supplementary file 2 — Source data Fig. 1 [file 44321_2025_319_MOESM2_ESM.zip › Figure 1/Panel F/5_1 HBMEC_HBVP devices/PC63_12_Before_0minutes.tif]

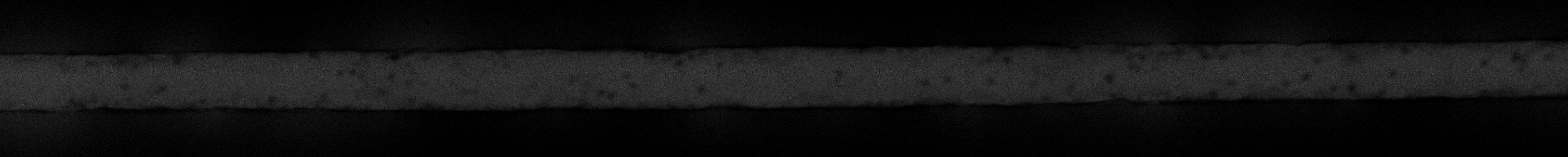

Supplement: Supplementary file 2 — Source data Fig. 1 [file 44321_2025_319_MOESM2_ESM.zip › Figure 1/Panel F/5_1 HBMEC_HBVP devices/PC63_12_Before_5minutes.tif]

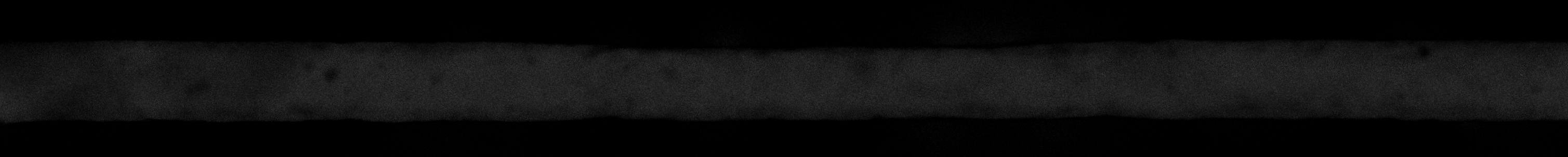

Supplement: Supplementary file 2 — Source data Fig. 1 [file 44321_2025_319_MOESM2_ESM.zip › Figure 1/Panel F/5_1 HBMEC_HBVP devices/PC63_3_Before_5min_channeloutline.tif]

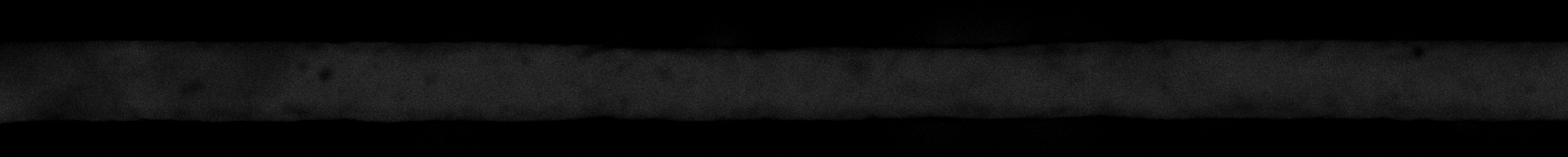

Supplement: Supplementary file 2 — Source data Fig. 1 [file 44321_2025_319_MOESM2_ESM.zip › Figure 1/Panel F/5_1 HBMEC_HBVP devices/PC63_3_Before_5minutes.tif]

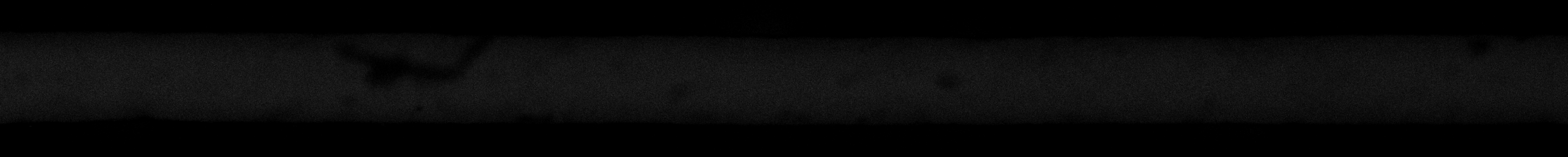

Supplement: Supplementary file 2 — Source data Fig. 1 [file 44321_2025_319_MOESM2_ESM.zip › Figure 1/Panel F/5_1 HBMEC_HBVP devices/PC63_5_Before_Time0.tif]

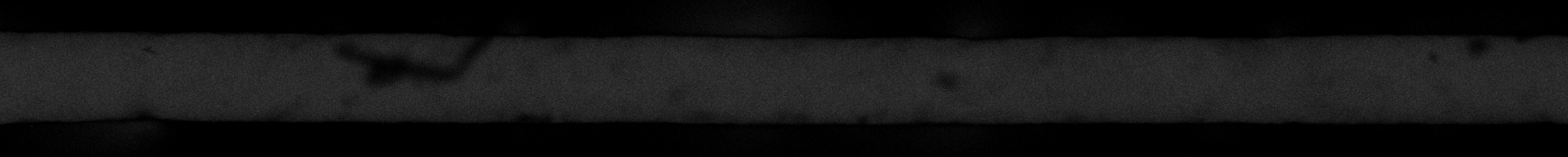

Supplement: Supplementary file 2 — Source data Fig. 1 [file 44321_2025_319_MOESM2_ESM.zip › Figure 1/Panel F/5_1 HBMEC_HBVP devices/PC63_5_Before_Time5.tif]

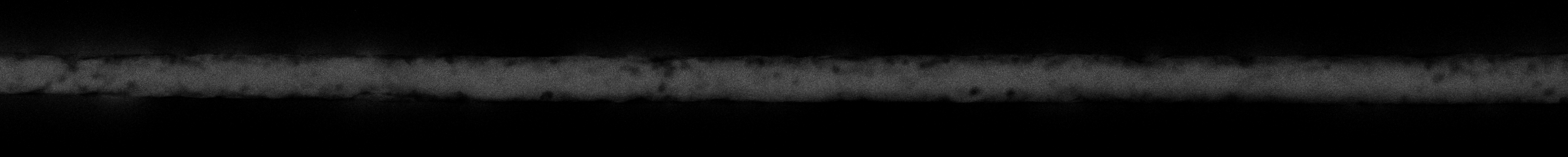

Supplement: Supplementary file 2 — Source data Fig. 1 [file 44321_2025_319_MOESM2_ESM.zip › Figure 1/Panel F/5_1 HBMEC_HBVP devices/PC64_1_Before_0min_channeloutline.tif]

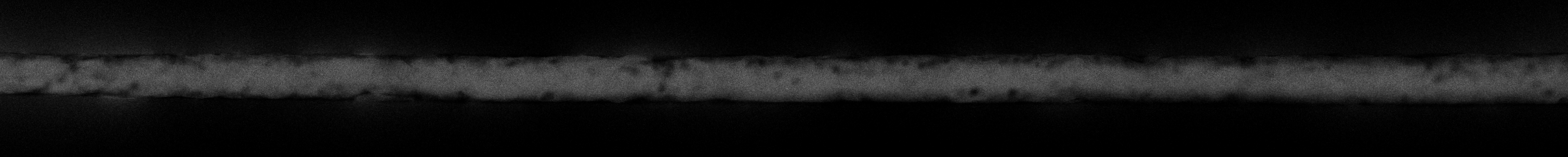

Supplement: Supplementary file 2 — Source data Fig. 1 [file 44321_2025_319_MOESM2_ESM.zip › Figure 1/Panel F/5_1 HBMEC_HBVP devices/PC64_1_Before_5min_channeloutline.tif]

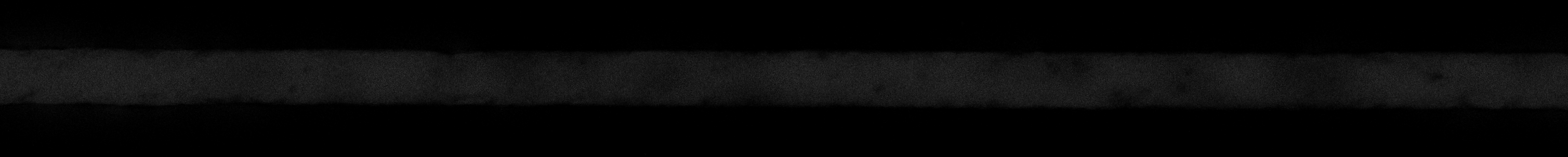

Supplement: Supplementary file 2 — Source data Fig. 1 [file 44321_2025_319_MOESM2_ESM.zip › Figure 1/Panel F/5_1 HBMEC_HBVP devices/PC64_10_Before_0min_Channeloutline.tif]

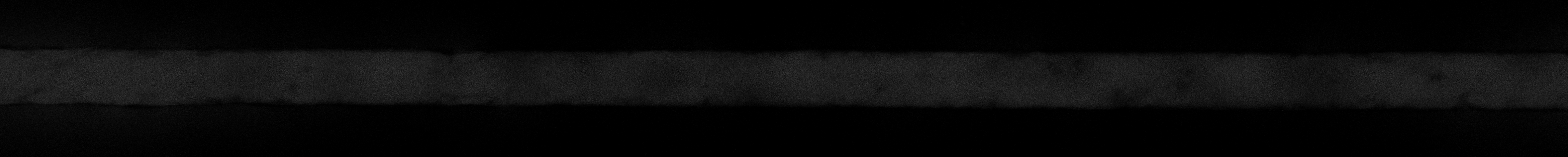

Supplement: Supplementary file 2 — Source data Fig. 1 [file 44321_2025_319_MOESM2_ESM.zip › Figure 1/Panel F/5_1 HBMEC_HBVP devices/PC64_10_Before_5min_Channeloutline.tif]

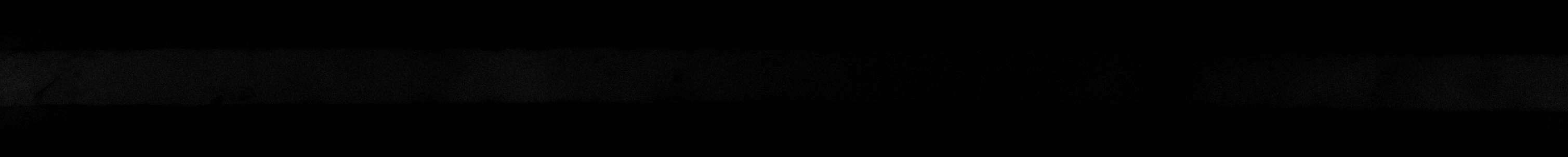

Supplement: Supplementary file 2 — Source data Fig. 1 [file 44321_2025_319_MOESM2_ESM.zip › Figure 1/Panel F/5_1 HBMEC_HBVP devices/PC64_8_Before_0min_Channeloutline.tif]

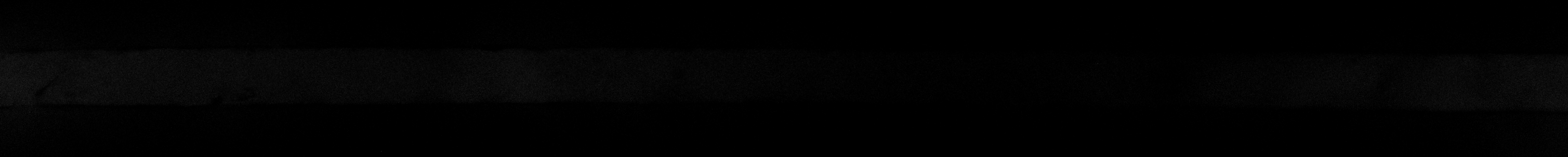

Supplement: Supplementary file 2 — Source data Fig. 1 [file 44321_2025_319_MOESM2_ESM.zip › Figure 1/Panel F/5_1 HBMEC_HBVP devices/PC64_8_Before_5min_Channeloutline.tif]

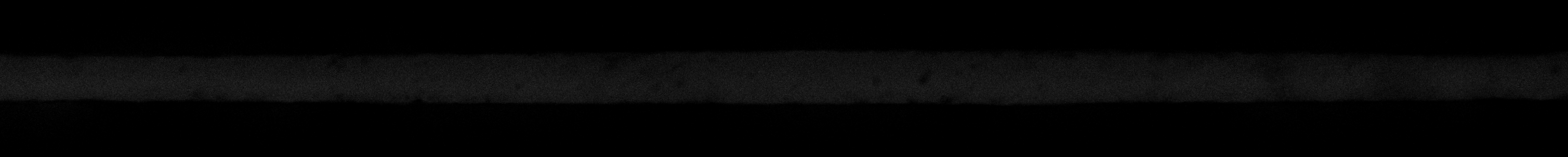

Supplement: Supplementary file 2 — Source data Fig. 1 [file 44321_2025_319_MOESM2_ESM.zip › Figure 1/Panel F/5_1 HBMEC_HBVP devices/PC64_9_Before_0min_channeloutline.tif]

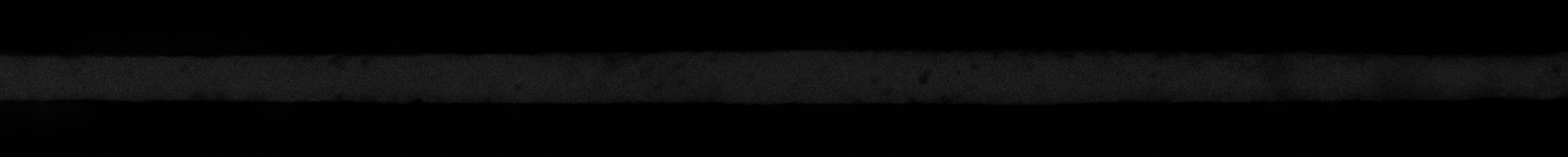

Supplement: Supplementary file 2 — Source data Fig. 1 [file 44321_2025_319_MOESM2_ESM.zip › Figure 1/Panel F/5_1 HBMEC_HBVP devices/PC64_9_Before_5min_Channeloutline.tif]

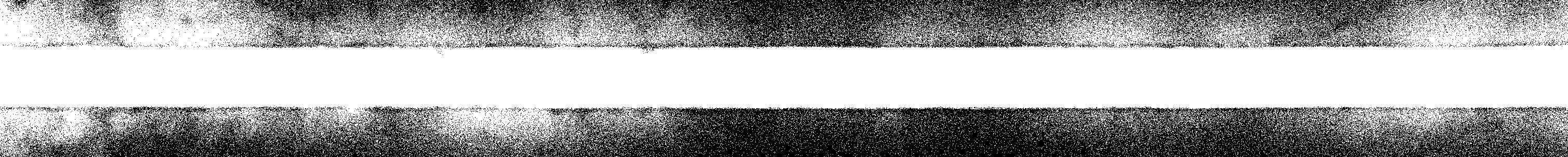

Supplement: Supplementary file 2 — Source data Fig. 1 [file 44321_2025_319_MOESM2_ESM.zip › Figure 1/Panel F/5_1 HBMEC_HBVP devices/PC79_1_Top_before_Jan292025_channel14.tif]

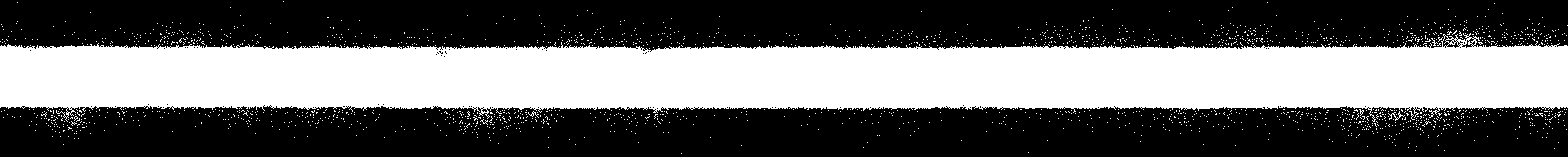

Supplement: Supplementary file 2 — Source data Fig. 1 [file 44321_2025_319_MOESM2_ESM.zip › Figure 1/Panel F/5_1 HBMEC_HBVP devices/PC79_1_Top_before_Jan292025_channel4.tif]

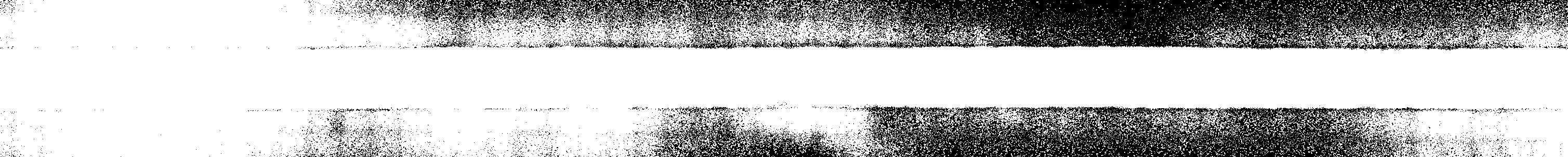

Supplement: Supplementary file 2 — Source data Fig. 1 [file 44321_2025_319_MOESM2_ESM.zip › Figure 1/Panel F/5_1 HBMEC_HBVP devices/PC79_10_Bottom_Before_Jan28_channel15.tif]

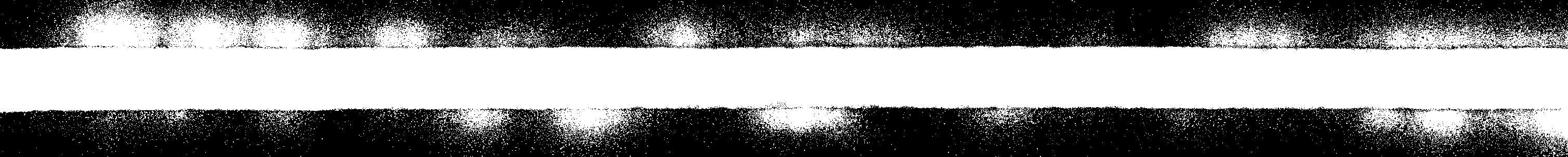

Supplement: Supplementary file 2 — Source data Fig. 1 [file 44321_2025_319_MOESM2_ESM.zip › Figure 1/Panel F/5_1 HBMEC_HBVP devices/PC79_10_Bottom_Before_Jan28_channel5.tif]

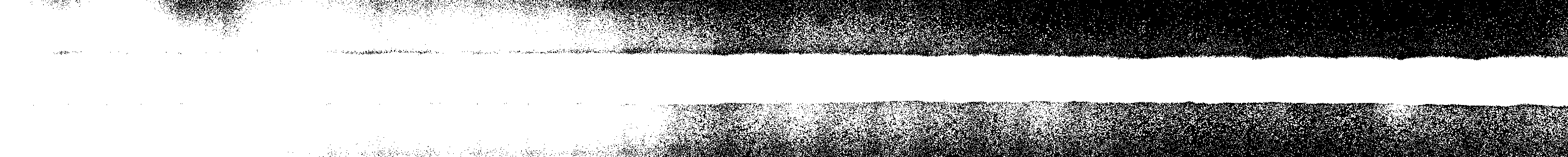

Supplement: Supplementary file 2 — Source data Fig. 1 [file 44321_2025_319_MOESM2_ESM.zip › Figure 1/Panel F/5_1 HBMEC_HBVP devices/PC79_11_Bottom_Before_Jan28_channel15.tif]

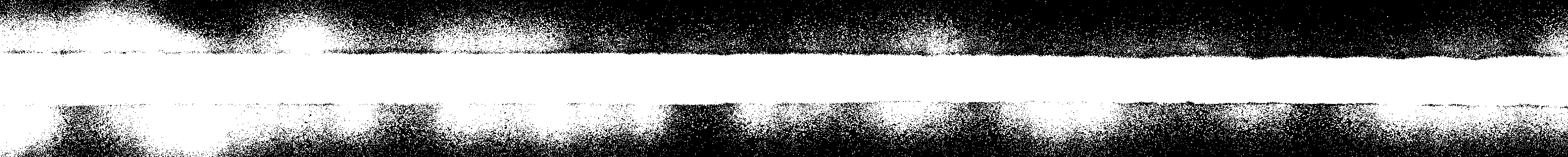

Supplement: Supplementary file 2 — Source data Fig. 1 [file 44321_2025_319_MOESM2_ESM.zip › Figure 1/Panel F/5_1 HBMEC_HBVP devices/PC79_11_Bottom_Before_Jan28_channel5.tif]

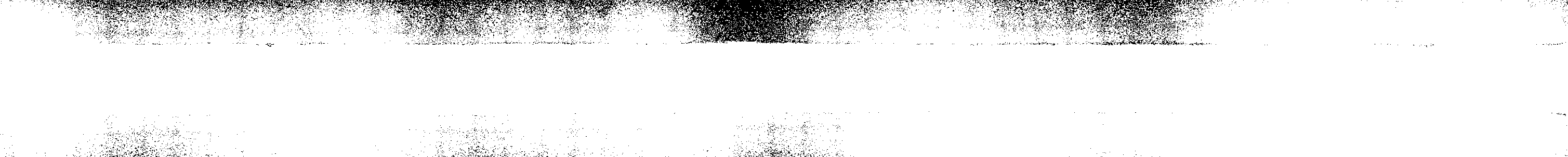

Supplement: Supplementary file 2 — Source data Fig. 1 [file 44321_2025_319_MOESM2_ESM.zip › Figure 1/Panel F/5_1 HBMEC_HBVP devices/PC79_12_Bottom_Before_Jan28_channel16.tif]

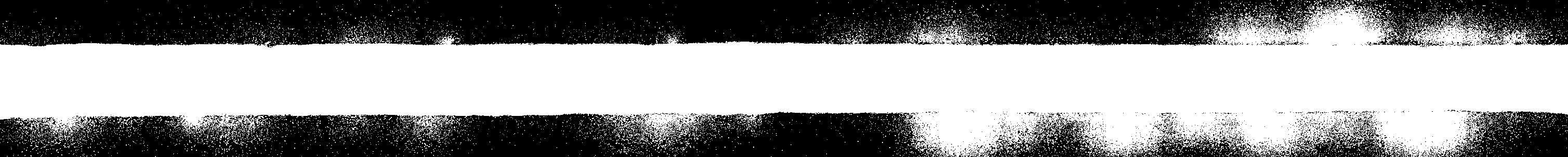

Supplement: Supplementary file 2 — Source data Fig. 1 [file 44321_2025_319_MOESM2_ESM.zip › Figure 1/Panel F/5_1 HBMEC_HBVP devices/PC79_12_Bottom_Before_Jan28_channel6.tif]

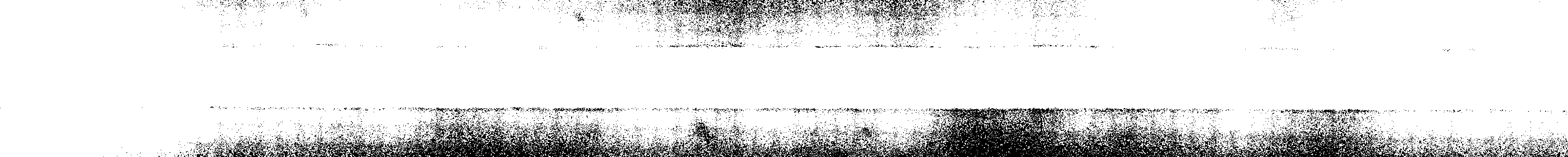

Supplement: Supplementary file 2 — Source data Fig. 1 [file 44321_2025_319_MOESM2_ESM.zip › Figure 1/Panel F/5_1 HBMEC_HBVP devices/PC79_13_Top_Before_Jan28_channel15.tif]

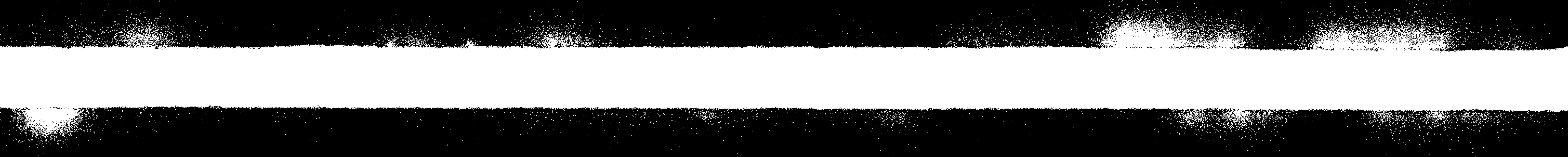

Supplement: Supplementary file 2 — Source data Fig. 1 [file 44321_2025_319_MOESM2_ESM.zip › Figure 1/Panel F/5_1 HBMEC_HBVP devices/PC79_13_Top_Before_Jan28_channel5.tif]

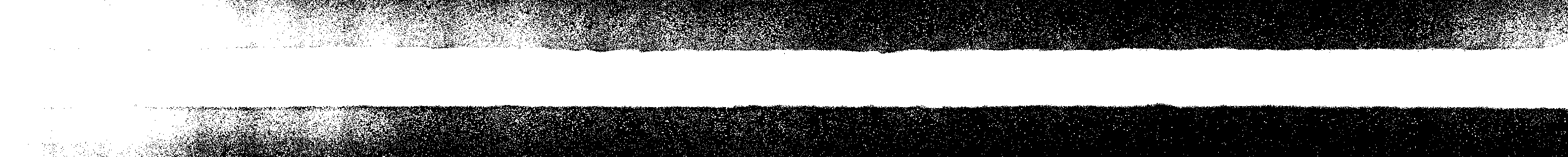

Supplement: Supplementary file 2 — Source data Fig. 1 [file 44321_2025_319_MOESM2_ESM.zip › Figure 1/Panel F/5_1 HBMEC_HBVP devices/PC79_14_Top_Before_Jan28_channel15.tif]

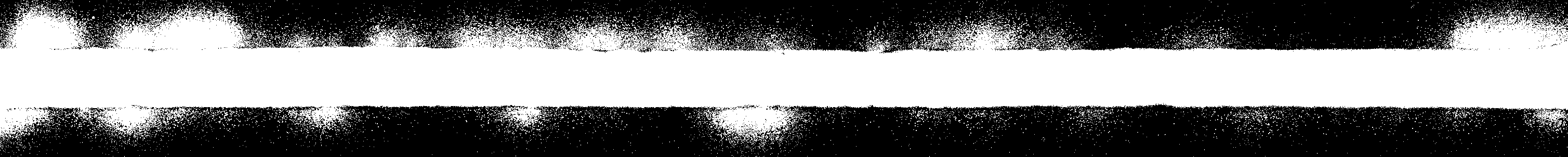

Supplement: Supplementary file 2 — Source data Fig. 1 [file 44321_2025_319_MOESM2_ESM.zip › Figure 1/Panel F/5_1 HBMEC_HBVP devices/PC79_14_Top_Before_Jan28_channel5.tif]

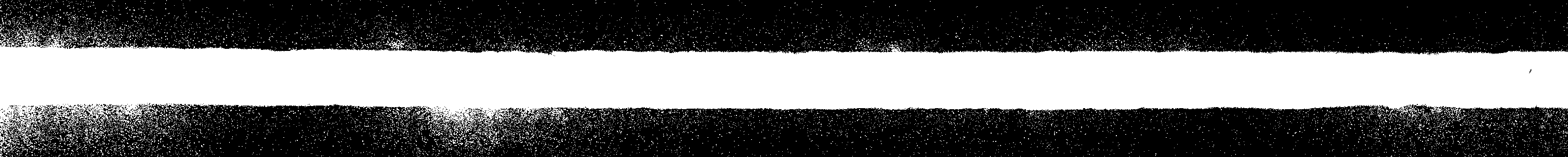

Supplement: Supplementary file 2 — Source data Fig. 1 [file 44321_2025_319_MOESM2_ESM.zip › Figure 1/Panel F/5_1 HBMEC_HBVP devices/PC79_15_Bottom_Before_Jan28_channel15.tif]

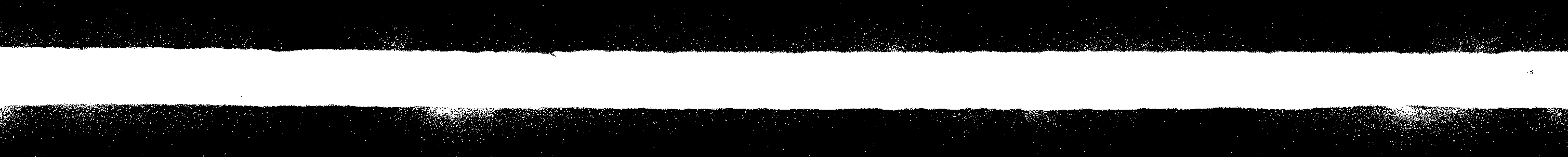

Supplement: Supplementary file 2 — Source data Fig. 1 [file 44321_2025_319_MOESM2_ESM.zip › Figure 1/Panel F/5_1 HBMEC_HBVP devices/PC79_15_Bottom_Before_Jan28_channel5.tif]

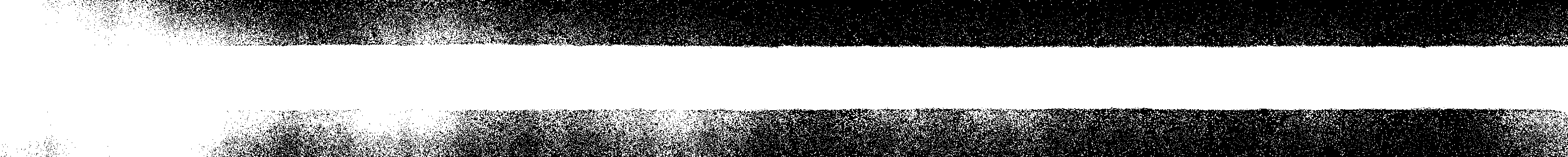

Supplement: Supplementary file 2 — Source data Fig. 1 [file 44321_2025_319_MOESM2_ESM.zip › Figure 1/Panel F/5_1 HBMEC_HBVP devices/PC79_16_Bottom_Before_Jan28_channel15.tif]

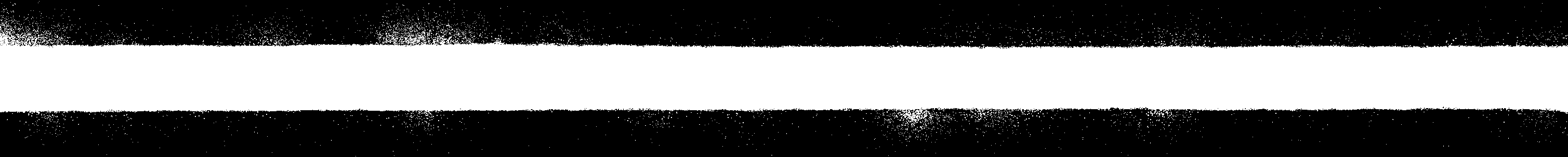

Supplement: Supplementary file 2 — Source data Fig. 1 [file 44321_2025_319_MOESM2_ESM.zip › Figure 1/Panel F/5_1 HBMEC_HBVP devices/PC79_16_Bottom_Before_Jan28_channel5.tif]

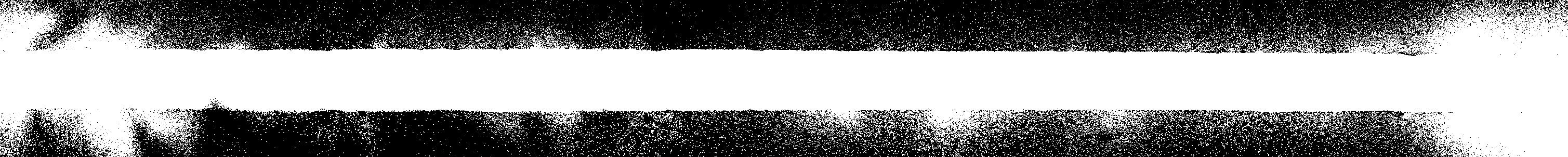

Supplement: Supplementary file 2 — Source data Fig. 1 [file 44321_2025_319_MOESM2_ESM.zip › Figure 1/Panel F/5_1 HBMEC_HBVP devices/PC79_2_Bottom_Before_AKB_Jan28_channel16.tif]

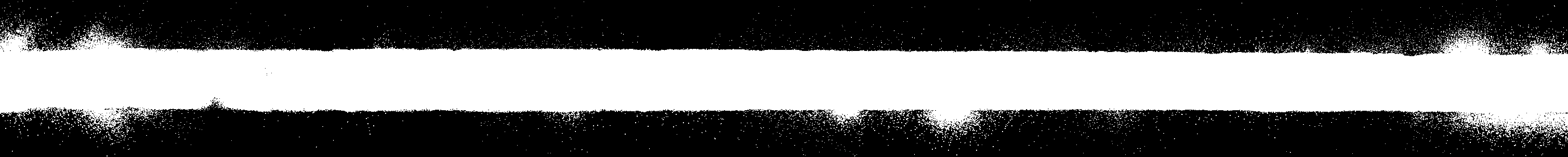

Supplement: Supplementary file 2 — Source data Fig. 1 [file 44321_2025_319_MOESM2_ESM.zip › Figure 1/Panel F/5_1 HBMEC_HBVP devices/PC79_2_Bottom_Before_AKB_Jan28_channel6.tif]

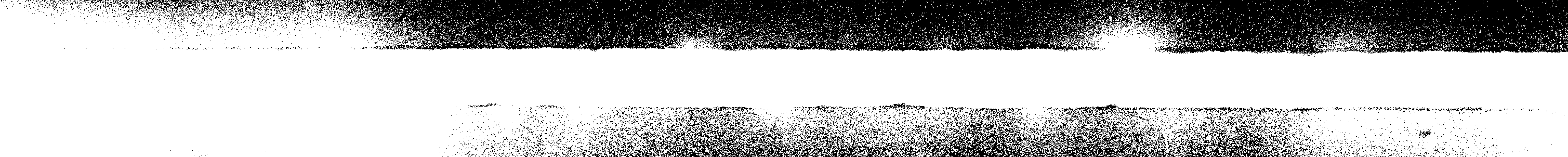

Supplement: Supplementary file 2 — Source data Fig. 1 [file 44321_2025_319_MOESM2_ESM.zip › Figure 1/Panel F/5_1 HBMEC_HBVP devices/PC79_5_Bottom_Before_Jan28_channel15.tif]

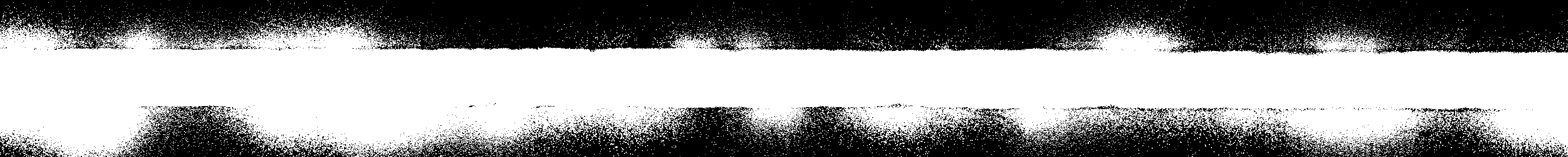

Supplement: Supplementary file 2 — Source data Fig. 1 [file 44321_2025_319_MOESM2_ESM.zip › Figure 1/Panel F/5_1 HBMEC_HBVP devices/PC79_5_Bottom_Before_Jan28_channel5.tif]

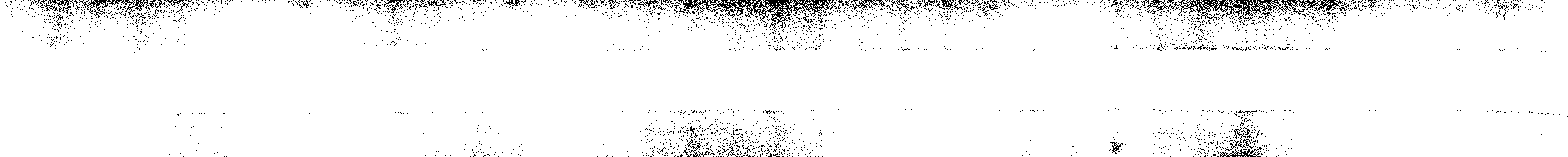

Supplement: Supplementary file 2 — Source data Fig. 1 [file 44321_2025_319_MOESM2_ESM.zip › Figure 1/Panel F/5_1 HBMEC_HBVP devices/PC79_6_Bottom_Before_Jan28_channel14.tif]

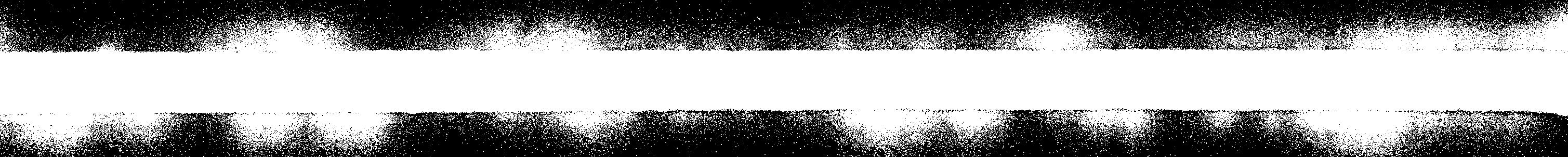

Supplement: Supplementary file 2 — Source data Fig. 1 [file 44321_2025_319_MOESM2_ESM.zip › Figure 1/Panel F/5_1 HBMEC_HBVP devices/PC79_6_Bottom_Before_Jan28_channel4.tif]

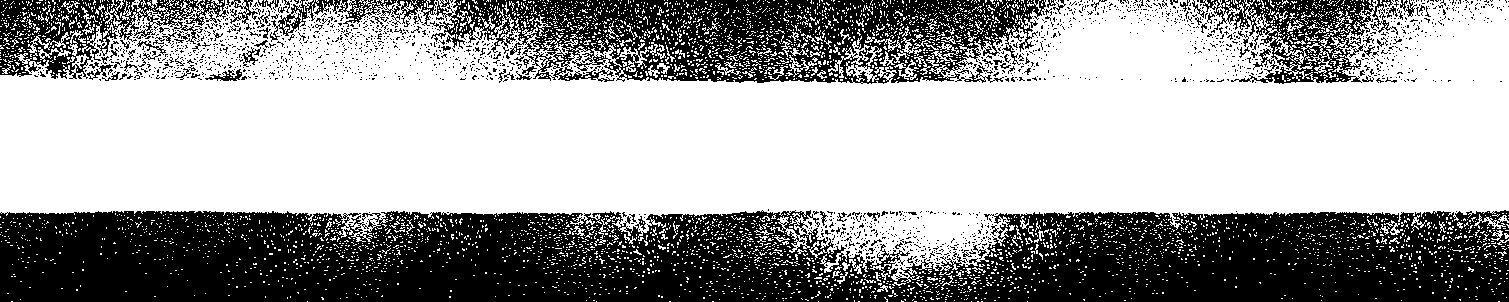

Supplement: Supplementary file 2 — Source data Fig. 1 [file 44321_2025_319_MOESM2_ESM.zip › Figure 1/Panel F/5_1 HBMEC_HBVP devices/PC79_8_Before_Jan28__only_left_channel16.tif]

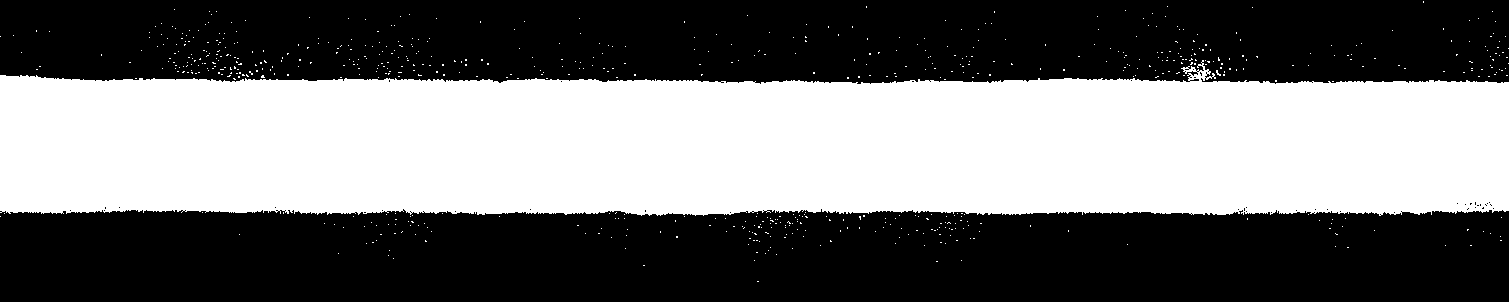

Supplement: Supplementary file 2 — Source data Fig. 1 [file 44321_2025_319_MOESM2_ESM.zip › Figure 1/Panel F/5_1 HBMEC_HBVP devices/PC79_8_Before_Jan28__only_left_channel6.tif]

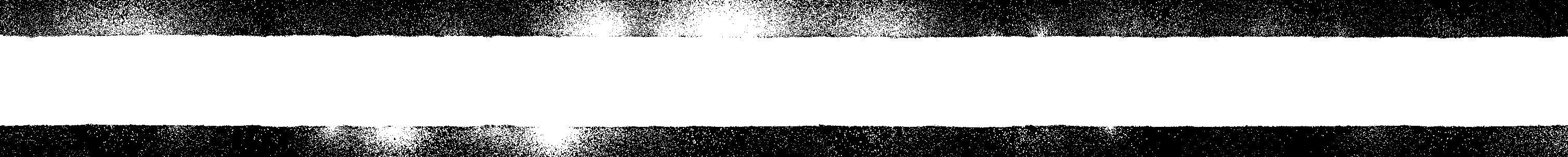

Supplement: Supplementary file 2 — Source data Fig. 1 [file 44321_2025_319_MOESM2_ESM.zip › Figure 1/Panel F/5_1 HBMEC_HBVP devices/PC79_9_Before_Jan28_channel15.tif]

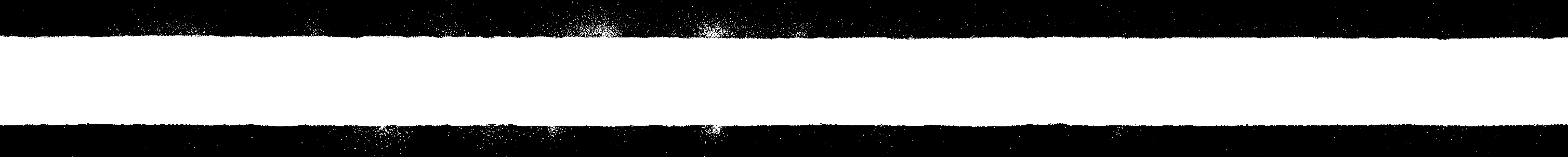

Supplement: Supplementary file 2 — Source data Fig. 1 [file 44321_2025_319_MOESM2_ESM.zip › Figure 1/Panel F/5_1 HBMEC_HBVP devices/PC79_9_Before_Jan28_channel5.tif]

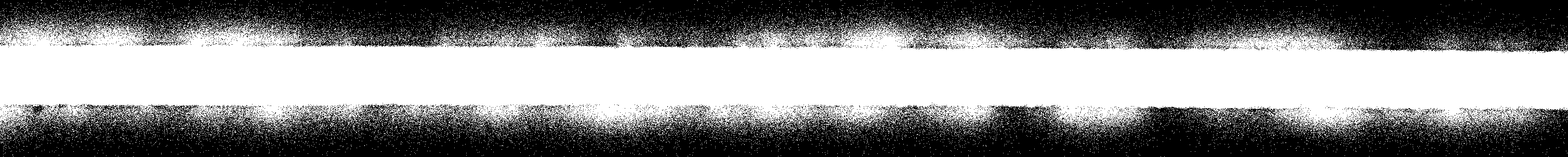

Supplement: Supplementary file 2 — Source data Fig. 1 [file 44321_2025_319_MOESM2_ESM.zip › Figure 1/Panel F/HBMEC only/PC77_11_HBMEC_only_Bottom_Jan82025_channel4.tif]

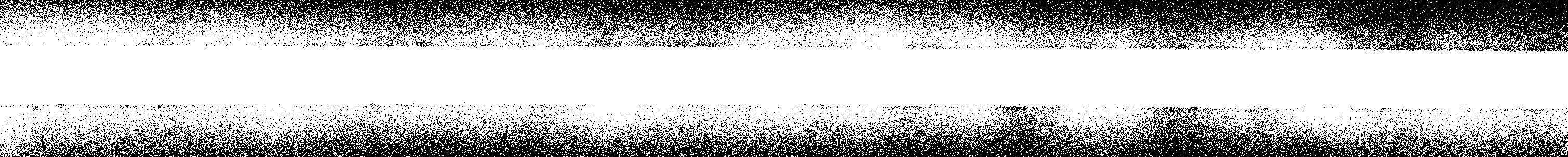

Supplement: Supplementary file 2 — Source data Fig. 1 [file 44321_2025_319_MOESM2_ESM.zip › Figure 1/Panel F/HBMEC only/PC77_11_HBMEC_only_Bottom_Jan82025_channel8.tif]

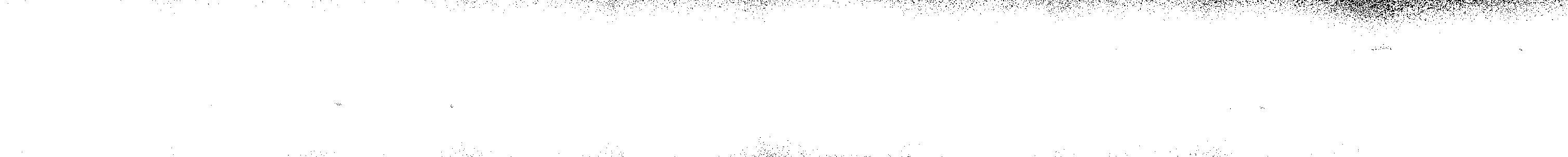

Supplement: Supplementary file 2 — Source data Fig. 1 [file 44321_2025_319_MOESM2_ESM.zip › Figure 1/Panel F/HBMEC only/PC77_12_HBMEC_only_Bottom_Jan82025_channel14.tif]

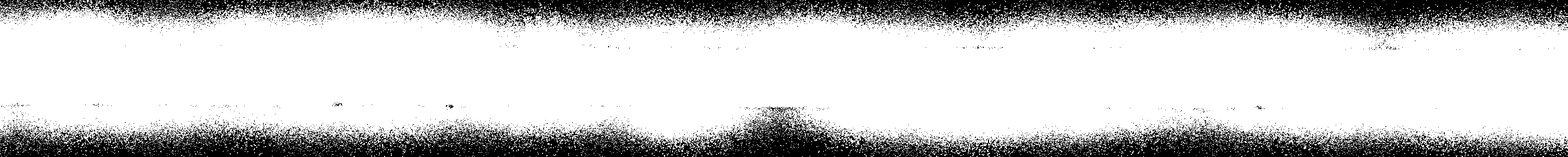

Supplement: Supplementary file 2 — Source data Fig. 1 [file 44321_2025_319_MOESM2_ESM.zip › Figure 1/Panel F/HBMEC only/PC77_12_HBMEC_only_Bottom_Jan82025_channel4.tif]

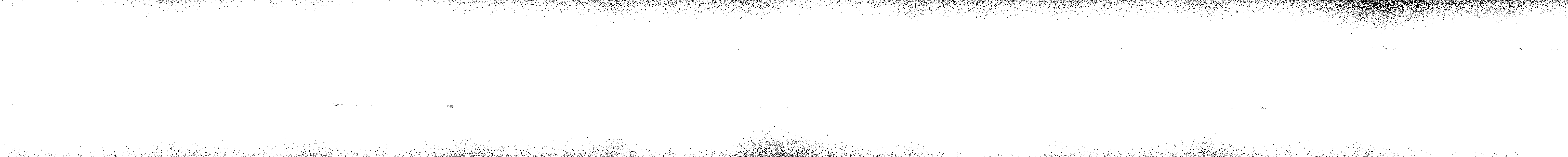

Supplement: Supplementary file 2 — Source data Fig. 1 [file 44321_2025_319_MOESM2_ESM.zip › Figure 1/Panel F/HBMEC only/PC77_12_HBMEC_only_Bottom_Jan82025_channel9.tif]

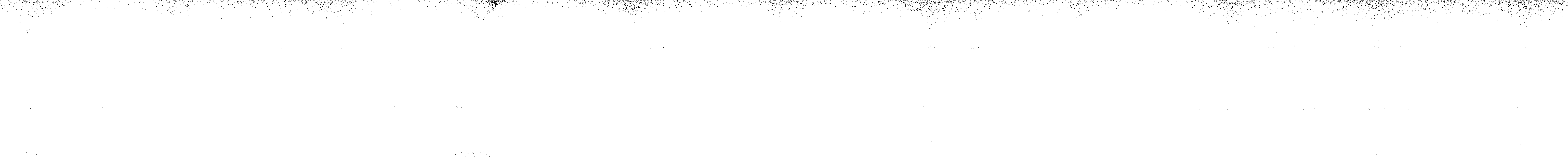

Supplement: Supplementary file 2 — Source data Fig. 1 [file 44321_2025_319_MOESM2_ESM.zip › Figure 1/Panel F/HBMEC only/PC77_2_HBMEC_only_Bottom_Jan82025_channel14.tif]

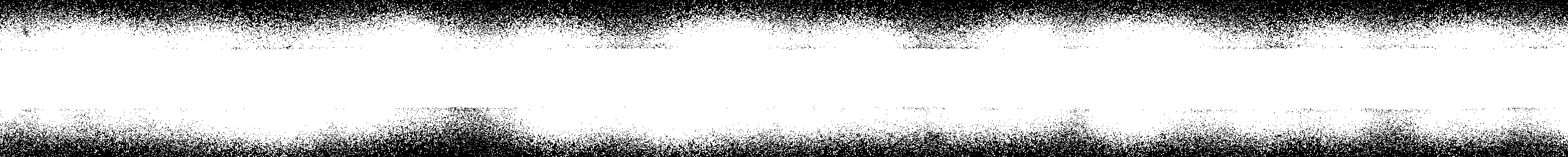

Supplement: Supplementary file 2 — Source data Fig. 1 [file 44321_2025_319_MOESM2_ESM.zip › Figure 1/Panel F/HBMEC only/PC77_2_HBMEC_only_Bottom_Jan82025_channel4.tif]

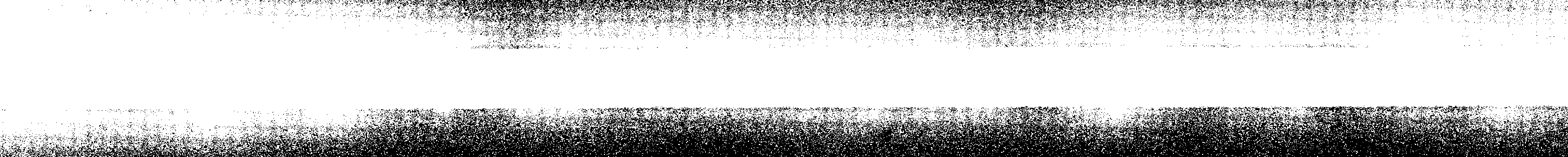

Supplement: Supplementary file 2 — Source data Fig. 1 [file 44321_2025_319_MOESM2_ESM.zip › Figure 1/Panel F/HBMEC only/PC77_3_HBMEC_only_Top_Jan82025_channel13.tif]

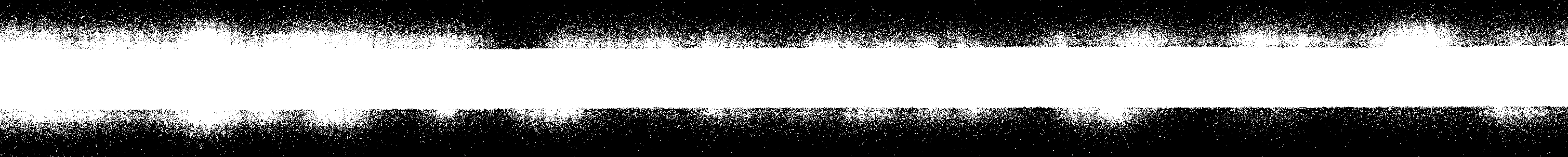

Supplement: Supplementary file 2 — Source data Fig. 1 [file 44321_2025_319_MOESM2_ESM.zip › Figure 1/Panel F/HBMEC only/PC77_3_HBMEC_only_Top_Jan82025_channel3.tif]

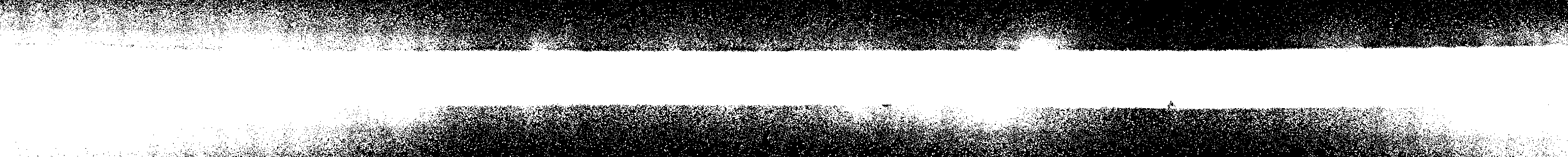

Supplement: Supplementary file 2 — Source data Fig. 1 [file 44321_2025_319_MOESM2_ESM.zip › Figure 1/Panel F/HBMEC only/PC77_4_HBMEC_only_Bottom_Jan82025_channel13.tif]

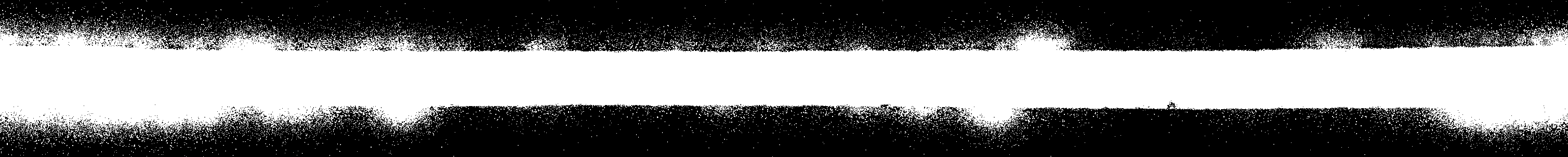

Supplement: Supplementary file 2 — Source data Fig. 1 [file 44321_2025_319_MOESM2_ESM.zip › Figure 1/Panel F/HBMEC only/PC77_4_HBMEC_only_Bottom_Jan82025_channel3.tif]

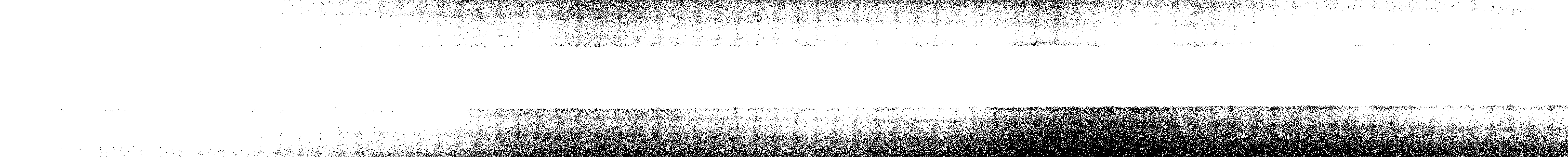

Supplement: Supplementary file 2 — Source data Fig. 1 [file 44321_2025_319_MOESM2_ESM.zip › Figure 1/Panel F/HBMEC only/PC77_5_HBMEC_only_Top_Jan82025_channel13.tif]

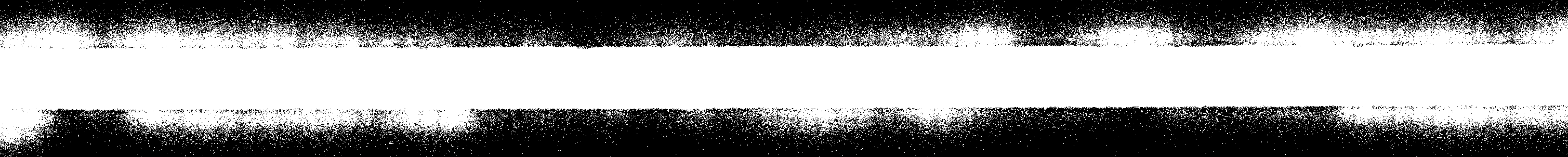

Supplement: Supplementary file 2 — Source data Fig. 1 [file 44321_2025_319_MOESM2_ESM.zip › Figure 1/Panel F/HBMEC only/PC77_5_HBMEC_only_Top_Jan82025_channel3.tif]

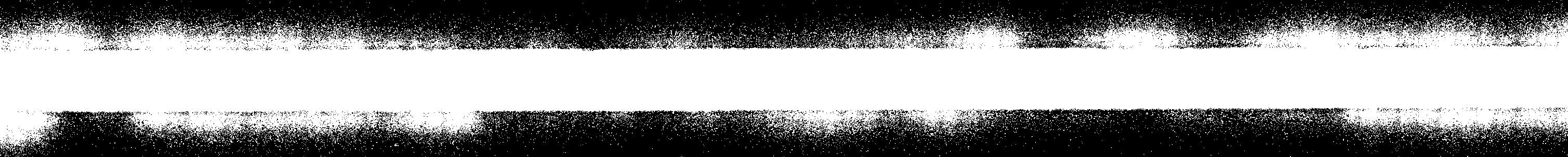

Supplement: Supplementary file 2 — Source data Fig. 1 [file 44321_2025_319_MOESM2_ESM.zip › Figure 1/Panel F/HBMEC only/PC77_5_HBMEC_only_Top_Jan82025_channel3_2.tif]

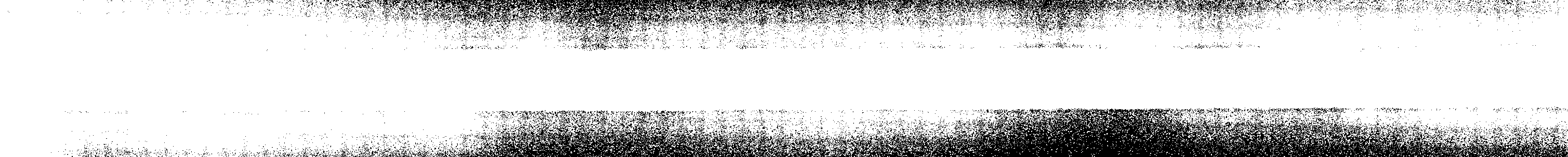

Supplement: Supplementary file 2 — Source data Fig. 1 [file 44321_2025_319_MOESM2_ESM.zip › Figure 1/Panel F/HBMEC only/PC77_5_HBMEC_only_Top_Jan82025_channel8_2.tif]

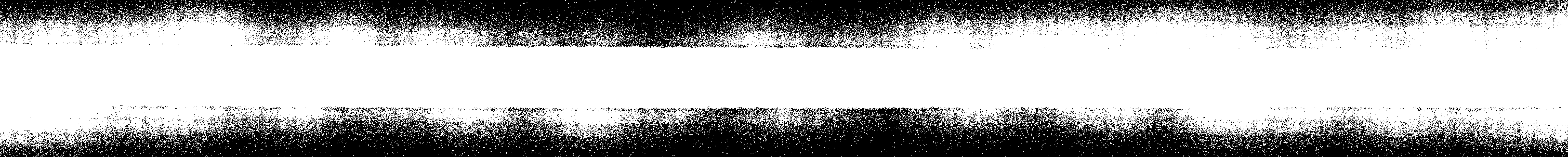

Supplement: Supplementary file 2 — Source data Fig. 1 [file 44321_2025_319_MOESM2_ESM.zip › Figure 1/Panel F/HBMEC only/PC77_6_HBMEC_only_Top_Jan82025_channel3.tif]

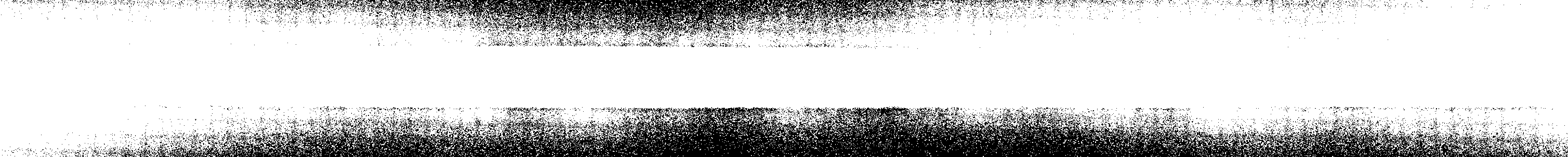

Supplement: Supplementary file 2 — Source data Fig. 1 [file 44321_2025_319_MOESM2_ESM.zip › Figure 1/Panel F/HBMEC only/PC77_6_HBMEC_only_Top_Jan82025_channel8.tif]

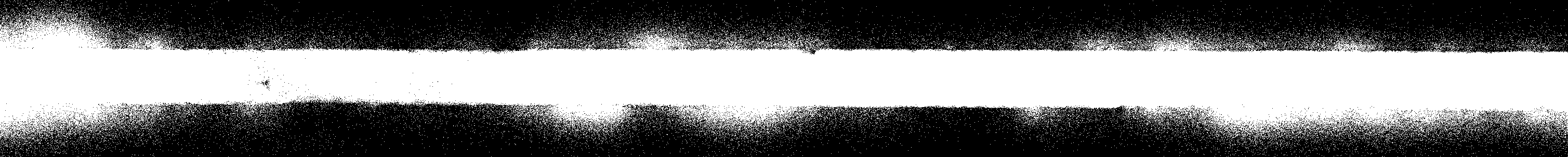

Supplement: Supplementary file 2 — Source data Fig. 1 [file 44321_2025_319_MOESM2_ESM.zip › Figure 1/Panel F/HBMEC only/PC77_7_HBMEC_only_Bottom_Jan82025_channel4.tif]

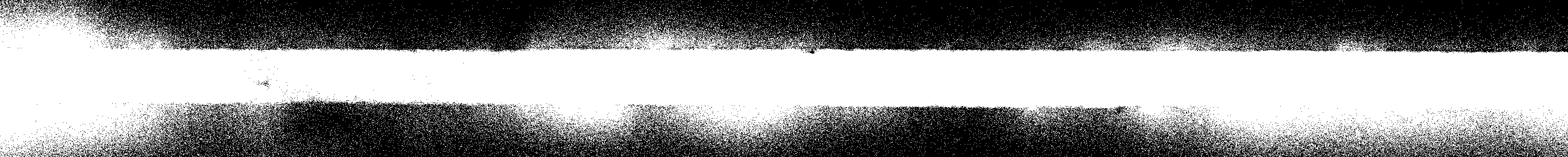

Supplement: Supplementary file 2 — Source data Fig. 1 [file 44321_2025_319_MOESM2_ESM.zip › Figure 1/Panel F/HBMEC only/PC77_7_HBMEC_only_Bottom_Jan82025_channel8.tif]

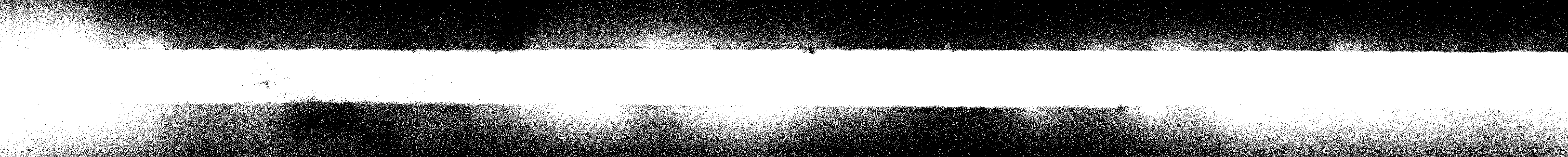

Supplement: Supplementary file 2 — Source data Fig. 1 [file 44321_2025_319_MOESM2_ESM.zip › Figure 1/Panel F/HBMEC only/PC77_7_HBMEC_only_Bottom_Jan82025_channel9.tif]

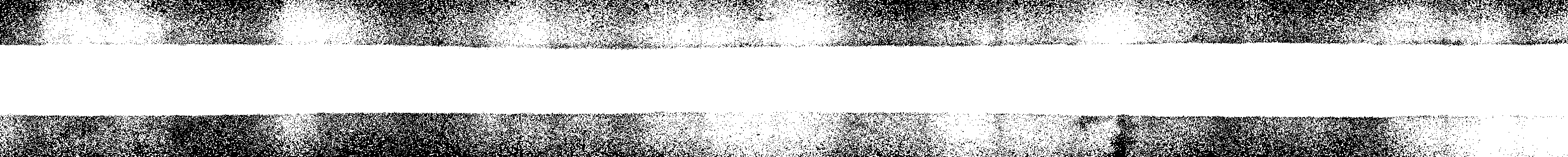

Supplement: Supplementary file 2 — Source data Fig. 1 [file 44321_2025_319_MOESM2_ESM.zip › Figure 1/Panel F/HBMEC only/PC79_17_HBMEC_only_Top_Jan282025_channel16.tif]

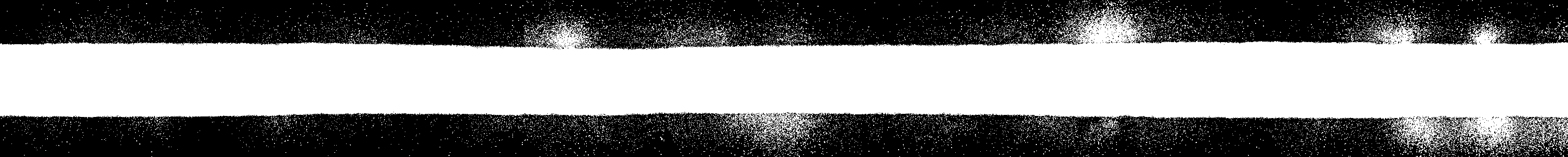

Supplement: Supplementary file 2 — Source data Fig. 1 [file 44321_2025_319_MOESM2_ESM.zip › Figure 1/Panel F/HBMEC only/PC79_17_HBMEC_only_Top_Jan282025_channel6.tif]

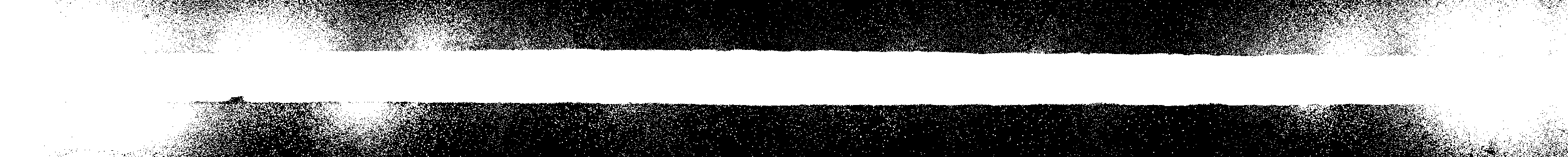

Supplement: Supplementary file 2 — Source data Fig. 1 [file 44321_2025_319_MOESM2_ESM.zip › Figure 1/Panel F/HBMEC only/PC79_18_HBMEC_only_Top_Jan282025_channel15.tif]

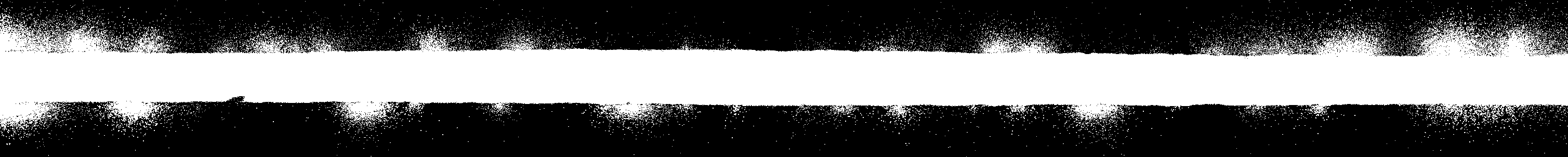

Supplement: Supplementary file 2 — Source data Fig. 1 [file 44321_2025_319_MOESM2_ESM.zip › Figure 1/Panel F/HBMEC only/PC79_18_HBMEC_only_Top_Jan282025_channel5.tif]

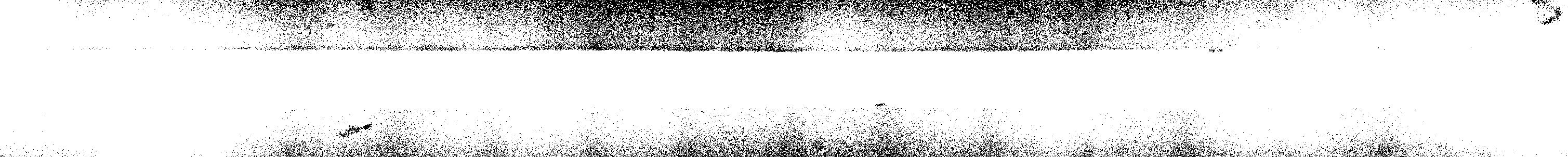

Supplement: Supplementary file 2 — Source data Fig. 1 [file 44321_2025_319_MOESM2_ESM.zip › Figure 1/Panel F/HBMEC only/PC79_19_HBMEC_only_Bottom_Jan282025_channel13.tif]

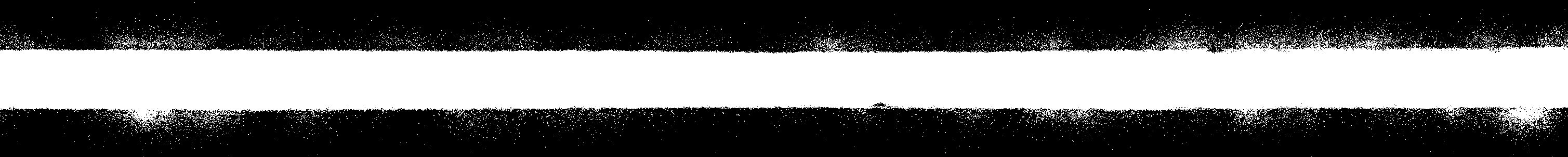

Supplement: Supplementary file 2 — Source data Fig. 1 [file 44321_2025_319_MOESM2_ESM.zip › Figure 1/Panel F/HBMEC only/PC79_19_HBMEC_only_Bottom_Jan282025_channel3.tif]

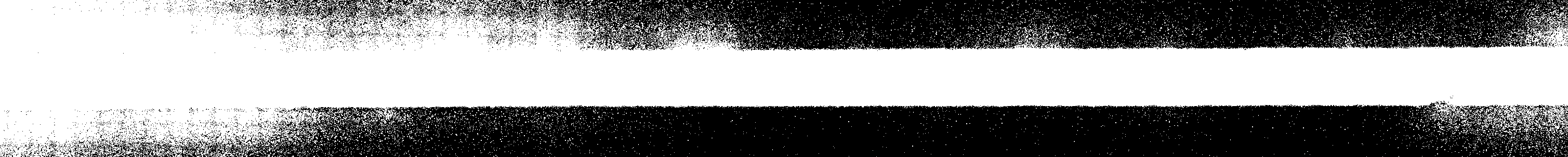

Supplement: Supplementary file 2 — Source data Fig. 1 [file 44321_2025_319_MOESM2_ESM.zip › Figure 1/Panel F/HBMEC only/PC79_20_HBMEC_only_Bottom_Jan282025_channel14.tif]

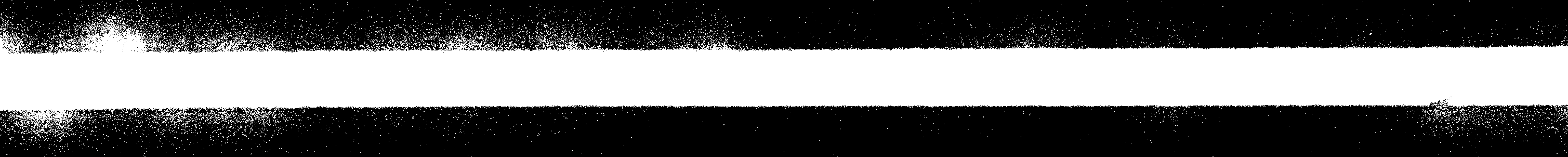

Supplement: Supplementary file 2 — Source data Fig. 1 [file 44321_2025_319_MOESM2_ESM.zip › Figure 1/Panel F/HBMEC only/PC79_20_HBMEC_only_Bottom_Jan282025_channel4.tif]

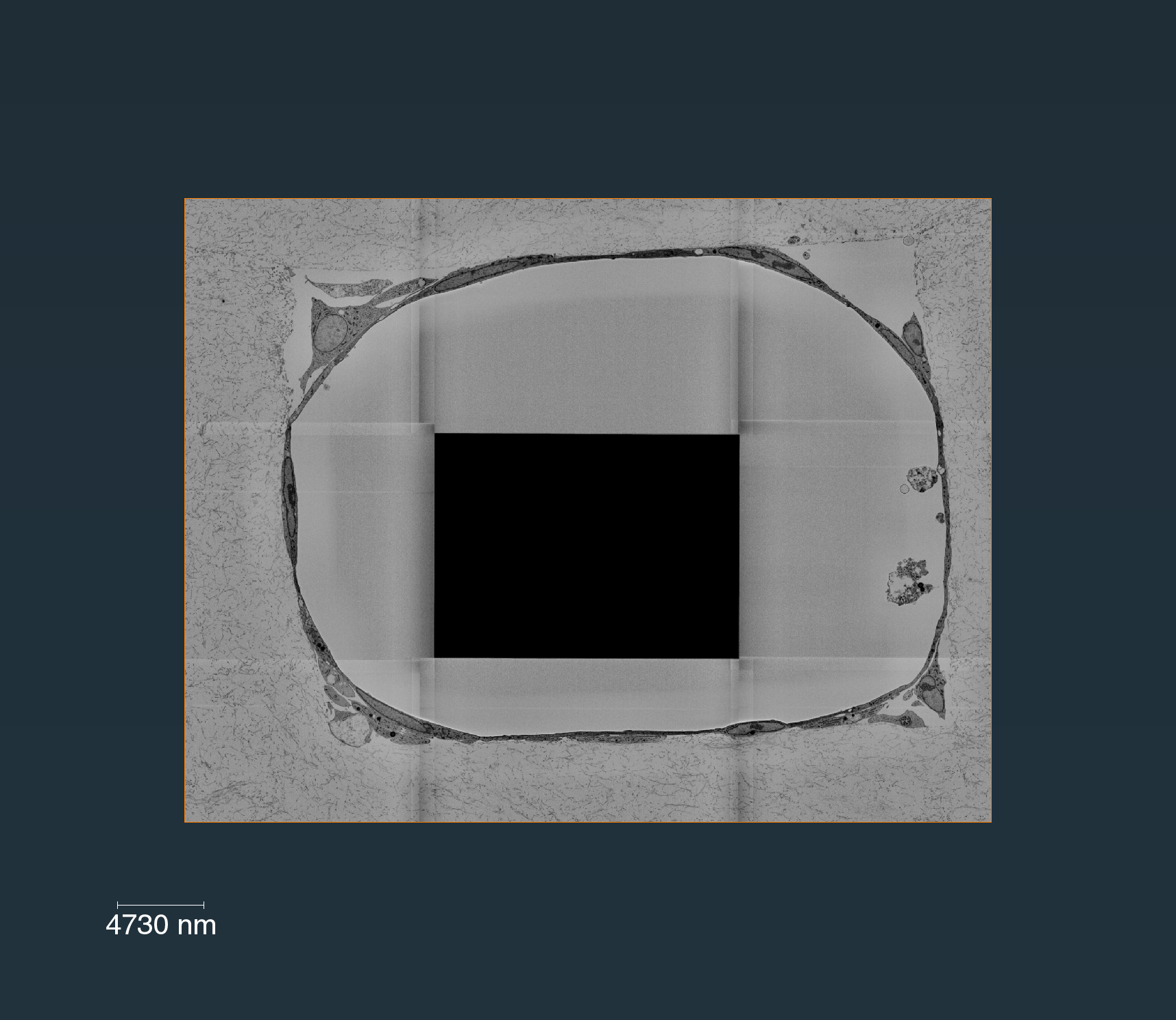

Supplement: Supplementary file 3 — Source data Fig. 2 [file 44321_2025_319_MOESM3_ESM.zip › Figure 2/Panel A and C/Amira_OrthoView_Slice228.png]

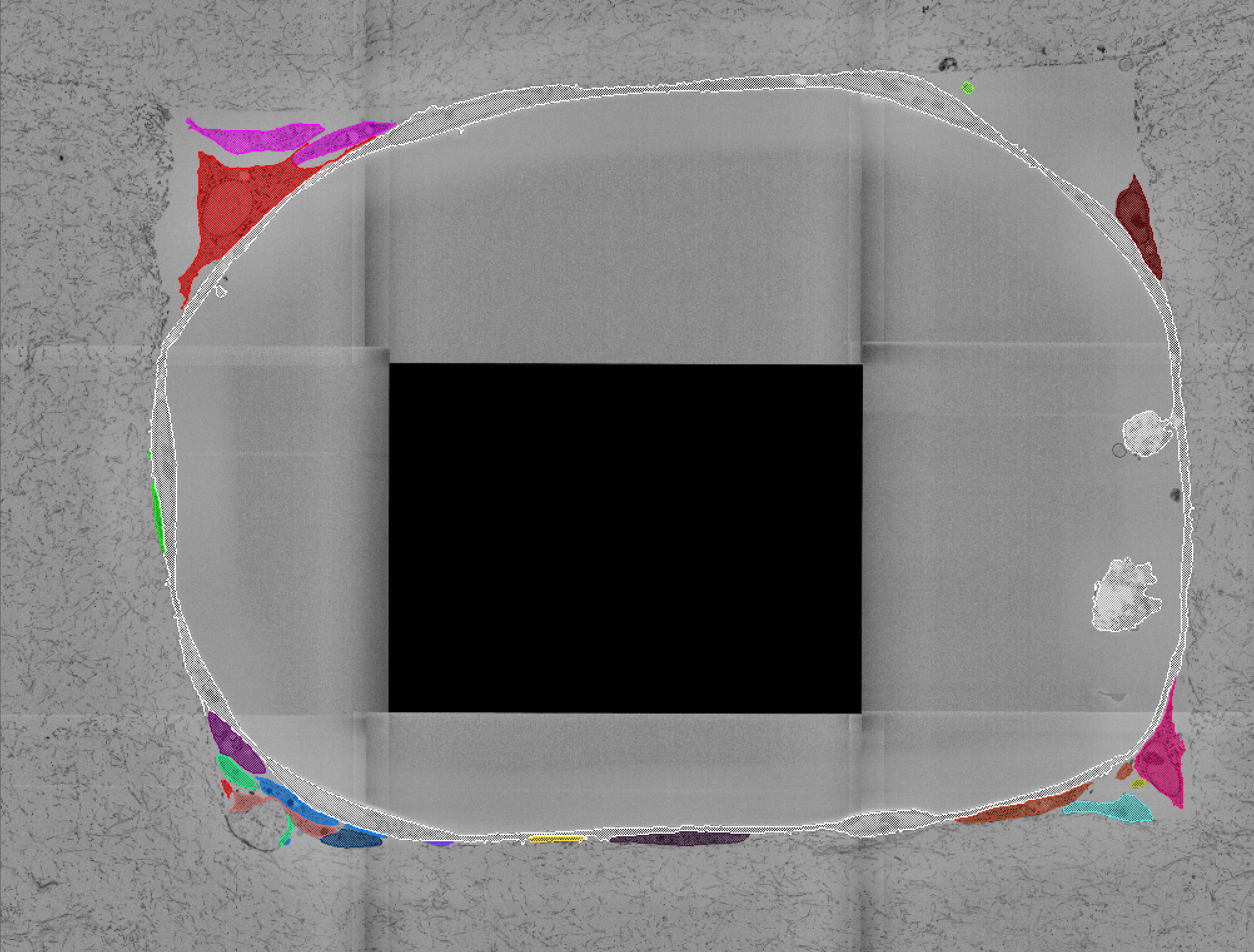

Supplement: Supplementary file 3 — Source data Fig. 2 [file 44321_2025_319_MOESM3_ESM.zip › Figure 2/Panel A and C/Amira_OrthoView_Slice228_Segmentation.PNG]

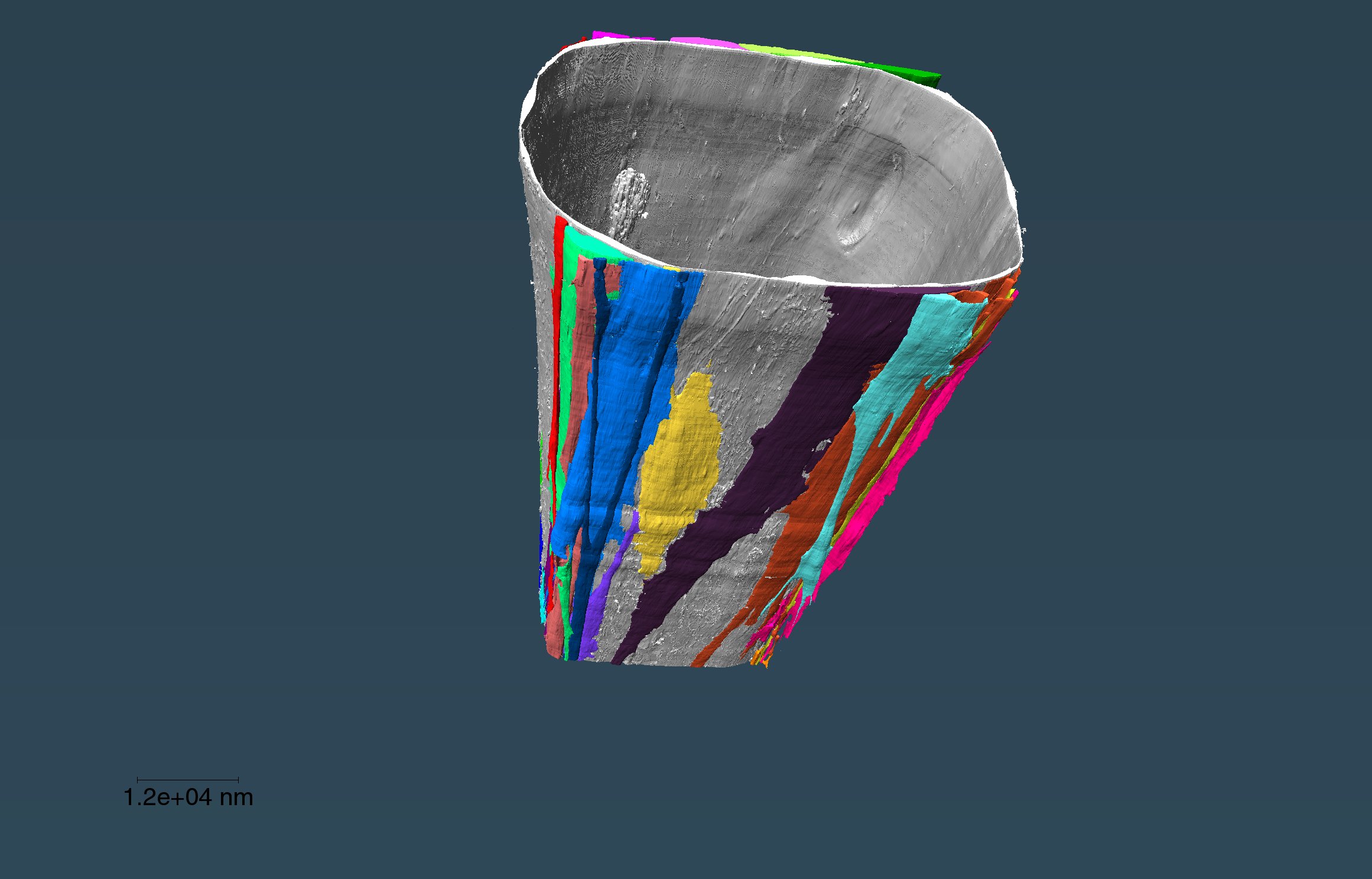

Supplement: Supplementary file 3 — Source data Fig. 2 [file 44321_2025_319_MOESM3_ESM.zip › Figure 2/Panel B/Vessel_Bottom_Surface_Complete_Endo_and_Pericytes_177.jpg]

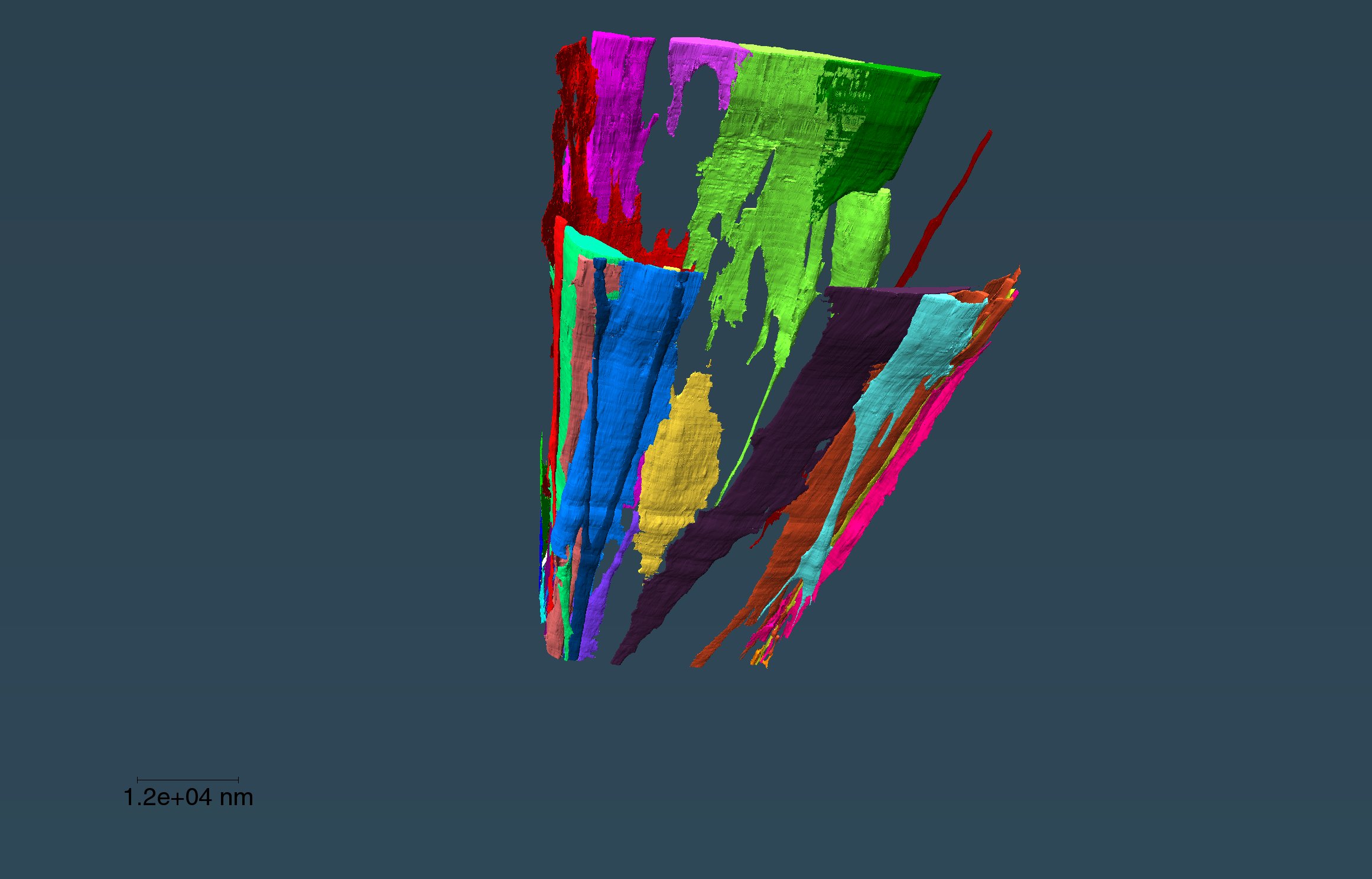

Supplement: Supplementary file 3 — Source data Fig. 2 [file 44321_2025_319_MOESM3_ESM.zip › Figure 2/Panel B/Vessel_Bottom_Surface_Complete_Pericytes_only_177.jpg]

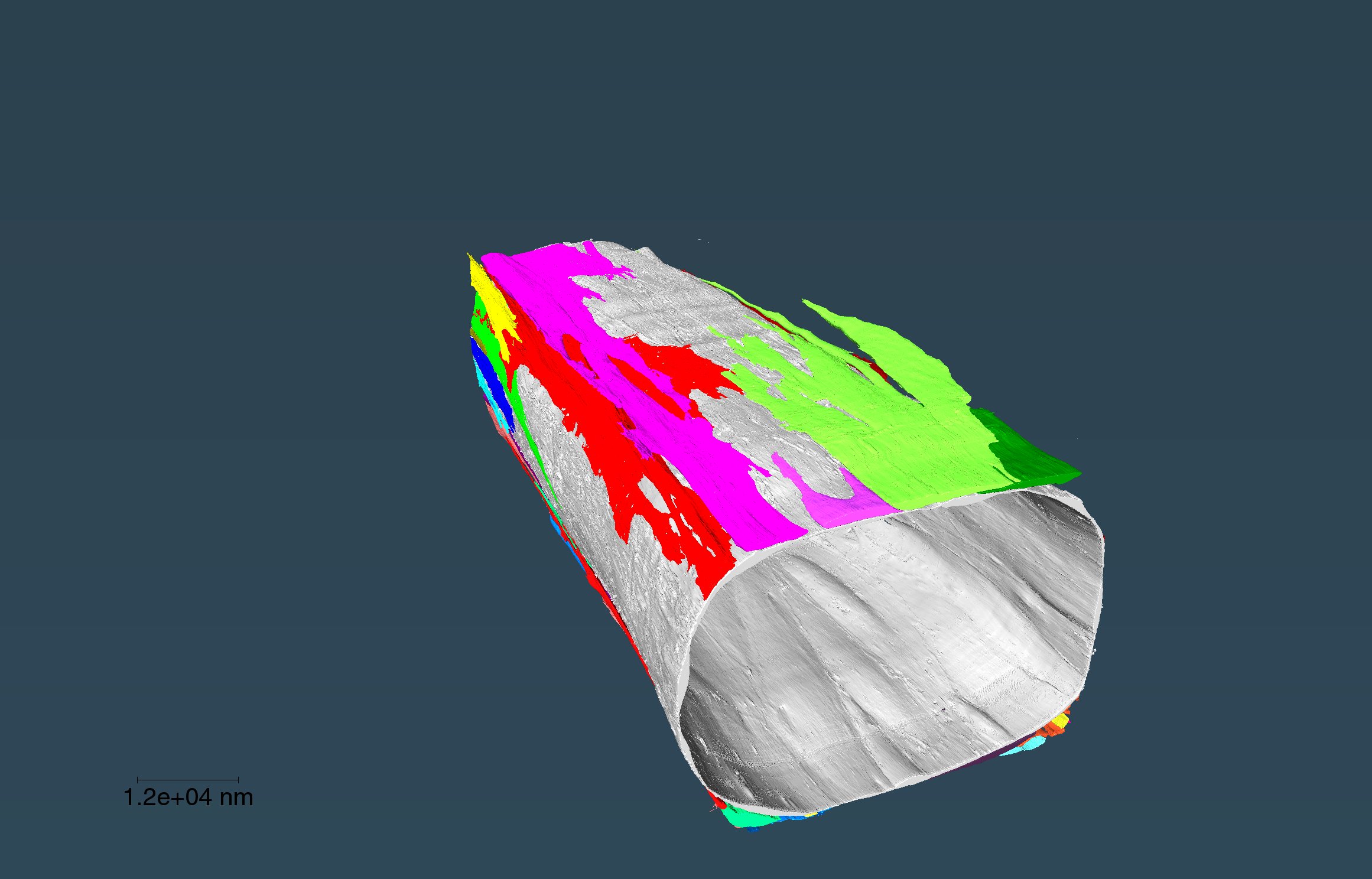

Supplement: Supplementary file 3 — Source data Fig. 2 [file 44321_2025_319_MOESM3_ESM.zip › Figure 2/Panel B/Vessel_Top_Surface_Complete_Endo_and_Pericytes_177.jpg]

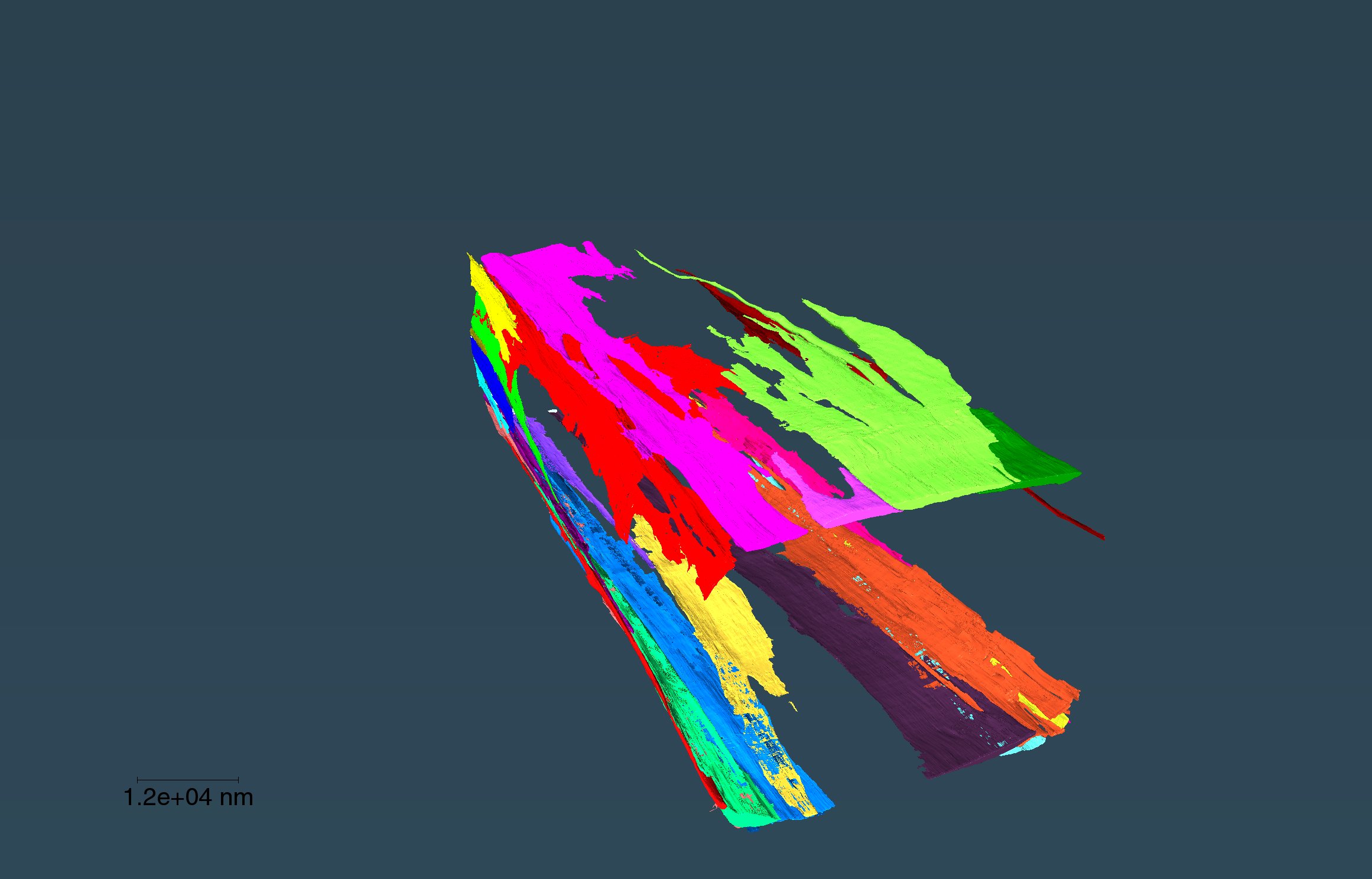

Supplement: Supplementary file 3 — Source data Fig. 2 [file 44321_2025_319_MOESM3_ESM.zip › Figure 2/Panel B/Vessel_Top_Surface_Complete_Pericytes_Only_177.jpg]

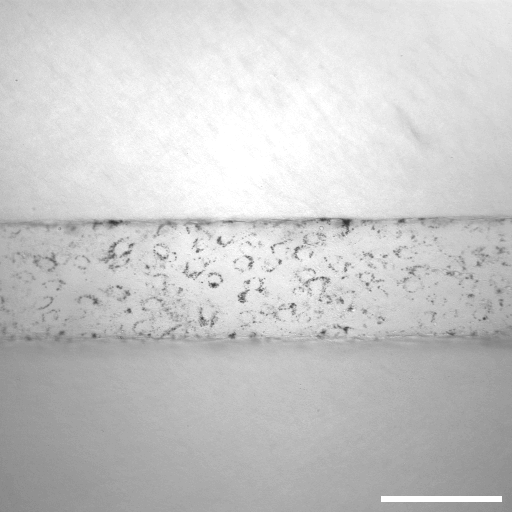

Supplement: Supplementary file 4 — Source data Fig. 3 [file 44321_2025_319_MOESM4_ESM.zip › Figure 3/Panel A/Brightfield_2Channel_Images/Bright_field_Microvessel_iRBC_Egress_Media.tif]

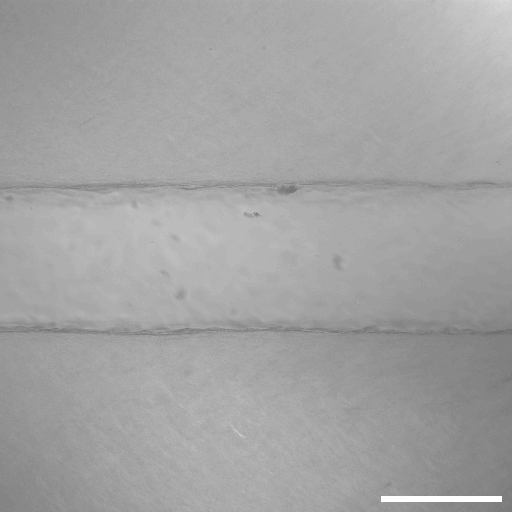

Supplement: Supplementary file 4 — Source data Fig. 3 [file 44321_2025_319_MOESM4_ESM.zip › Figure 3/Panel A/Brightfield_2Channel_Images/Bright_field_Microvessel_Media_only.tif]

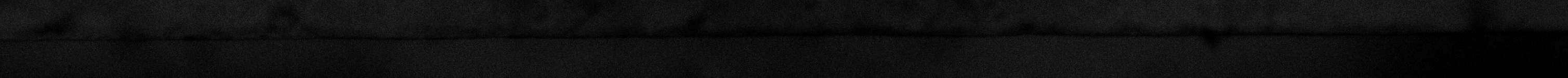

Supplement: Supplementary file 4 — Source data Fig. 3 [file 44321_2025_319_MOESM4_ESM.zip › Figure 3/Panel A/iRBC_Egress_Media_only_permeability_analysis_timepoint_masks/PC62_7_Bottom_RM_slice_12.tif]

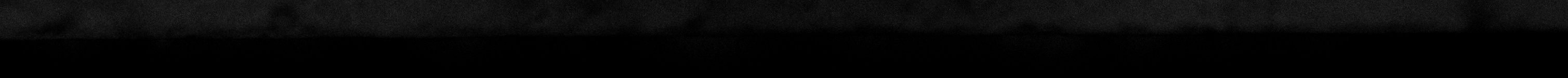

Supplement: Supplementary file 4 — Source data Fig. 3 [file 44321_2025_319_MOESM4_ESM.zip › Figure 3/Panel A/iRBC_Egress_Media_only_permeability_analysis_timepoint_masks/PC62_7_Bottom_RM_slice_2.tif]
