# Supplementary figures and images for: Plasmodium falciparum impairs Ang-1 secretion by pericytes in a 3D brain microvessel model (part 2 of 4)
Source: EMBO Mol Med. 2025 Oct 16;17(11):3110–38. doi: 10.1038/s44321-025-00319-y (PMC12603187; doi:10.1038/s44321-025-00319-y)

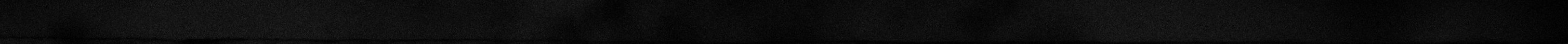

Supplement: Supplementary file 4 — Source data Fig. 3 [file 44321_2025_319_MOESM4_ESM.zip › Figure 3/Panel A/iRBC_Egress_Media_only_permeability_analysis_timepoint_masks/PC62_7_Top_RM_slice_12.tif]

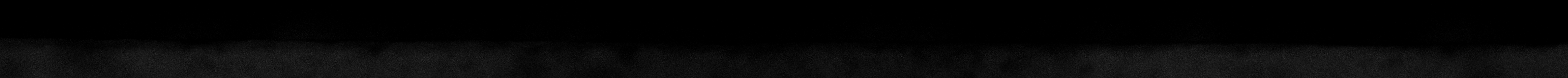

Supplement: Supplementary file 4 — Source data Fig. 3 [file 44321_2025_319_MOESM4_ESM.zip › Figure 3/Panel A/iRBC_Egress_Media_only_permeability_analysis_timepoint_masks/PC62_7_Top_RM_slice_2.tif]

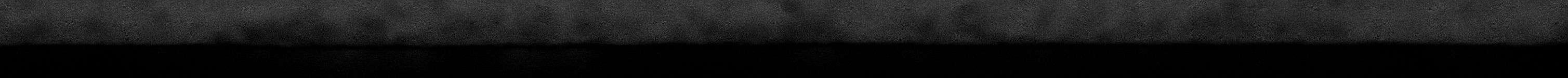

Supplement: Supplementary file 4 — Source data Fig. 3 [file 44321_2025_319_MOESM4_ESM.zip › Figure 3/Panel A/iRBC_Egress_Media_only_permeability_analysis_timepoint_masks/PC63_11_Bottom_RM_slice_13.tif]

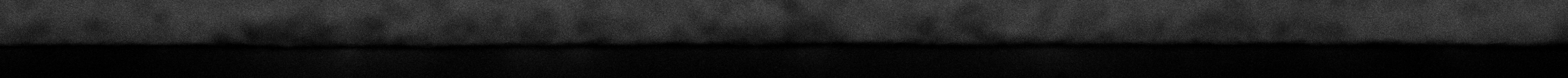

Supplement: Supplementary file 4 — Source data Fig. 3 [file 44321_2025_319_MOESM4_ESM.zip › Figure 3/Panel A/iRBC_Egress_Media_only_permeability_analysis_timepoint_masks/PC63_11_Bottom_RM_slice_23.tif]

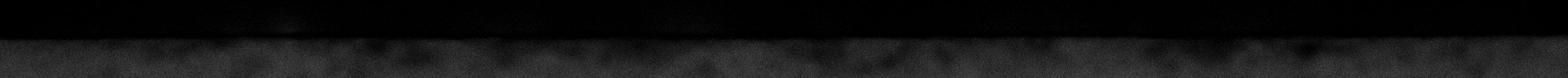

Supplement: Supplementary file 4 — Source data Fig. 3 [file 44321_2025_319_MOESM4_ESM.zip › Figure 3/Panel A/iRBC_Egress_Media_only_permeability_analysis_timepoint_masks/PC63_11_Top_RM_slice_13.tif]

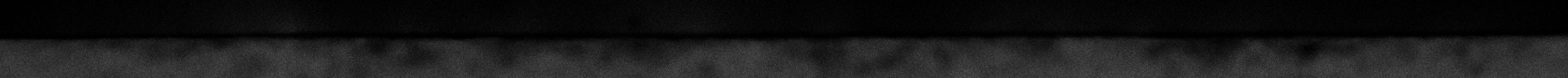

Supplement: Supplementary file 4 — Source data Fig. 3 [file 44321_2025_319_MOESM4_ESM.zip › Figure 3/Panel A/iRBC_Egress_Media_only_permeability_analysis_timepoint_masks/PC63_11_Top_RM_slice_23.tif]

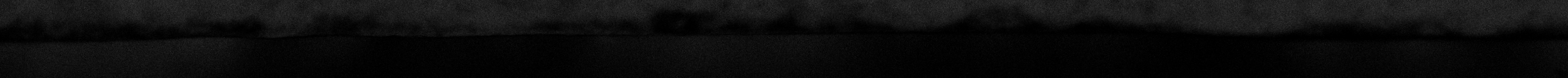

Supplement: Supplementary file 4 — Source data Fig. 3 [file 44321_2025_319_MOESM4_ESM.zip › Figure 3/Panel A/iRBC_Egress_Media_only_permeability_analysis_timepoint_masks/PC63_2_Bottom_RM_slice_18.tif]

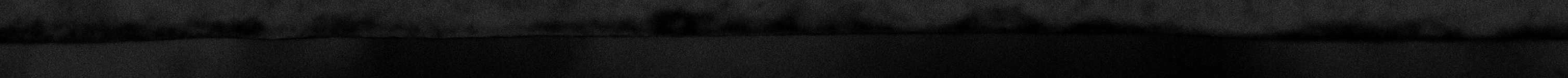

Supplement: Supplementary file 4 — Source data Fig. 3 [file 44321_2025_319_MOESM4_ESM.zip › Figure 3/Panel A/iRBC_Egress_Media_only_permeability_analysis_timepoint_masks/PC63_2_Bottom_RM_slice_28.tif]

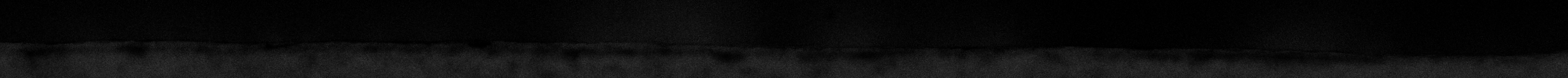

Supplement: Supplementary file 4 — Source data Fig. 3 [file 44321_2025_319_MOESM4_ESM.zip › Figure 3/Panel A/iRBC_Egress_Media_only_permeability_analysis_timepoint_masks/PC63_2_Top_RM_slice_18.tif]

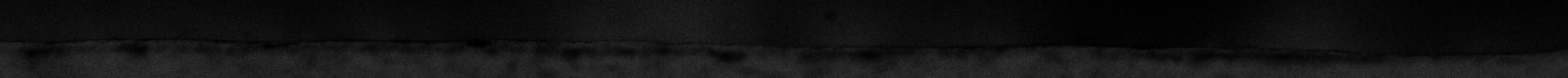

Supplement: Supplementary file 4 — Source data Fig. 3 [file 44321_2025_319_MOESM4_ESM.zip › Figure 3/Panel A/iRBC_Egress_Media_only_permeability_analysis_timepoint_masks/PC63_2_Top_RM_slice_28.tif]

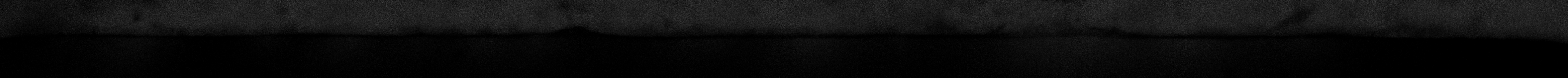

Supplement: Supplementary file 4 — Source data Fig. 3 [file 44321_2025_319_MOESM4_ESM.zip › Figure 3/Panel A/iRBC_Egress_Media_only_permeability_analysis_timepoint_masks/PC63_3_Bottom_SM_slice_12.tif]

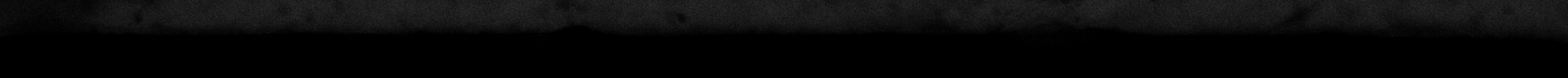

Supplement: Supplementary file 4 — Source data Fig. 3 [file 44321_2025_319_MOESM4_ESM.zip › Figure 3/Panel A/iRBC_Egress_Media_only_permeability_analysis_timepoint_masks/PC63_3_Bottom_SM_slice_2.tif]

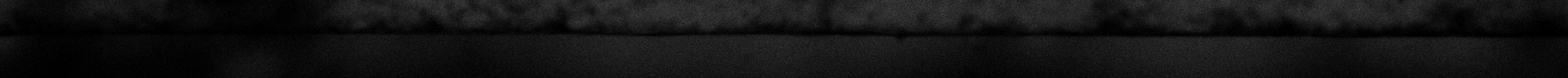

Supplement: Supplementary file 4 — Source data Fig. 3 [file 44321_2025_319_MOESM4_ESM.zip › Figure 3/Panel A/iRBC_Egress_Media_only_permeability_analysis_timepoint_masks/PC63_7_Bottom_RM_slice_11.tif]

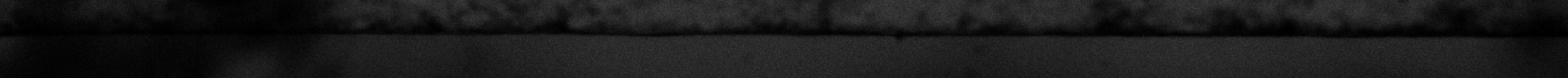

Supplement: Supplementary file 4 — Source data Fig. 3 [file 44321_2025_319_MOESM4_ESM.zip › Figure 3/Panel A/iRBC_Egress_Media_only_permeability_analysis_timepoint_masks/PC63_7_Bottom_RM_slice_21.tif]

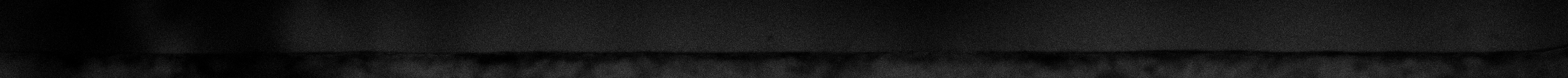

Supplement: Supplementary file 4 — Source data Fig. 3 [file 44321_2025_319_MOESM4_ESM.zip › Figure 3/Panel A/iRBC_Egress_Media_only_permeability_analysis_timepoint_masks/PC63_7_Top_RM_slice_11.tif]

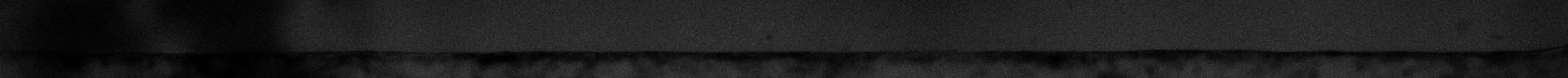

Supplement: Supplementary file 4 — Source data Fig. 3 [file 44321_2025_319_MOESM4_ESM.zip › Figure 3/Panel A/iRBC_Egress_Media_only_permeability_analysis_timepoint_masks/PC63_7_Top_RM_slice_21.tif]

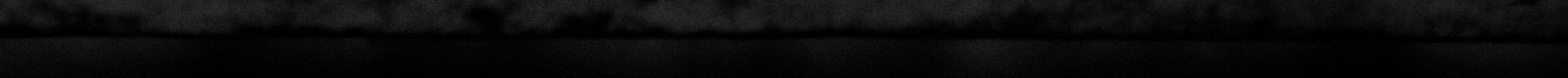

Supplement: Supplementary file 4 — Source data Fig. 3 [file 44321_2025_319_MOESM4_ESM.zip › Figure 3/Panel A/iRBC_Egress_Media_only_permeability_analysis_timepoint_masks/PC63_8_Bottom_RM_slice_12.tif]

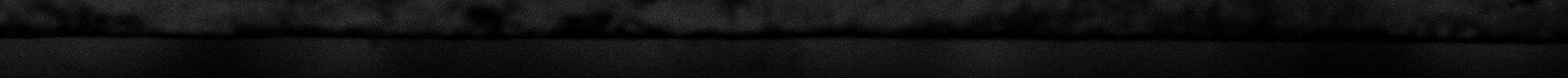

Supplement: Supplementary file 4 — Source data Fig. 3 [file 44321_2025_319_MOESM4_ESM.zip › Figure 3/Panel A/iRBC_Egress_Media_only_permeability_analysis_timepoint_masks/PC63_8_Bottom_RM_slice_22.tif]

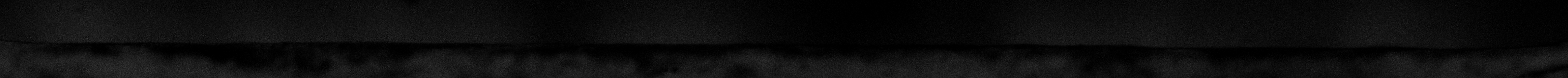

Supplement: Supplementary file 4 — Source data Fig. 3 [file 44321_2025_319_MOESM4_ESM.zip › Figure 3/Panel A/iRBC_Egress_Media_only_permeability_analysis_timepoint_masks/PC63_8_Top_RM_slice_12.tif]

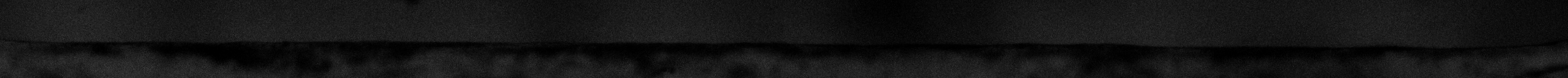

Supplement: Supplementary file 4 — Source data Fig. 3 [file 44321_2025_319_MOESM4_ESM.zip › Figure 3/Panel A/iRBC_Egress_Media_only_permeability_analysis_timepoint_masks/PC63_8_Top_RM_slice_22.tif]

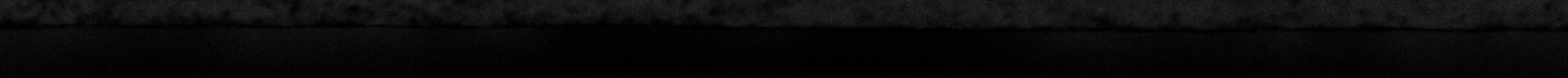

Supplement: Supplementary file 4 — Source data Fig. 3 [file 44321_2025_319_MOESM4_ESM.zip › Figure 3/Panel A/iRBC_Egress_Media_only_permeability_analysis_timepoint_masks/PC64_11_Bottom_RM_slice_19.tif]

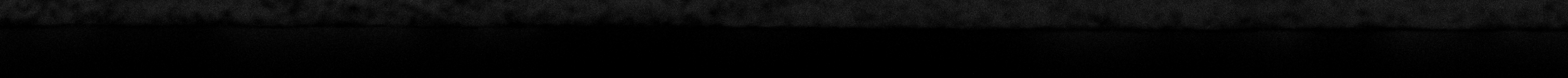

Supplement: Supplementary file 4 — Source data Fig. 3 [file 44321_2025_319_MOESM4_ESM.zip › Figure 3/Panel A/iRBC_Egress_Media_only_permeability_analysis_timepoint_masks/PC64_11_Bottom_RM_slice_9.tif]

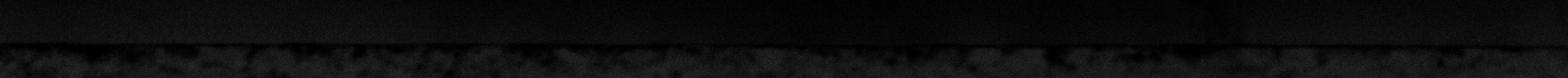

Supplement: Supplementary file 4 — Source data Fig. 3 [file 44321_2025_319_MOESM4_ESM.zip › Figure 3/Panel A/iRBC_Egress_Media_only_permeability_analysis_timepoint_masks/PC64_11_Top_RM_slice_19.tif]

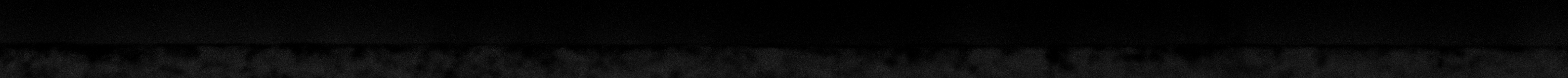

Supplement: Supplementary file 4 — Source data Fig. 3 [file 44321_2025_319_MOESM4_ESM.zip › Figure 3/Panel A/iRBC_Egress_Media_only_permeability_analysis_timepoint_masks/PC64_11_Top_RM_slice_9.tif]

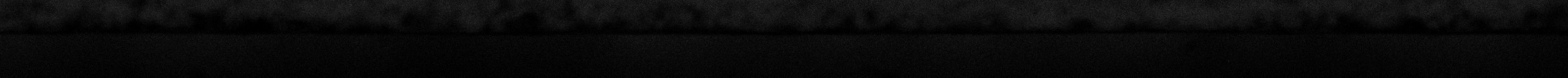

Supplement: Supplementary file 4 — Source data Fig. 3 [file 44321_2025_319_MOESM4_ESM.zip › Figure 3/Panel A/iRBC_Egress_Media_only_permeability_analysis_timepoint_masks/PC64_12_Bottom_RM_slice_19.tif]

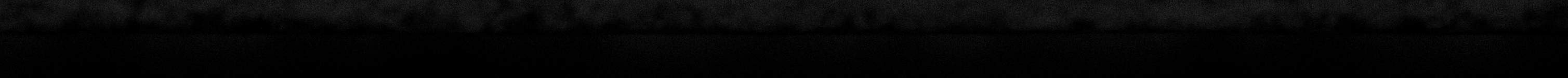

Supplement: Supplementary file 4 — Source data Fig. 3 [file 44321_2025_319_MOESM4_ESM.zip › Figure 3/Panel A/iRBC_Egress_Media_only_permeability_analysis_timepoint_masks/PC64_12_Bottom_RM_slice_9.tif]

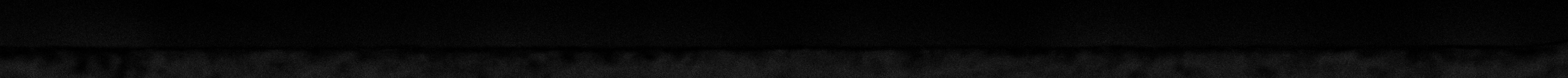

Supplement: Supplementary file 4 — Source data Fig. 3 [file 44321_2025_319_MOESM4_ESM.zip › Figure 3/Panel A/iRBC_Egress_Media_only_permeability_analysis_timepoint_masks/PC64_12_Top_RM_slice_19.tif]

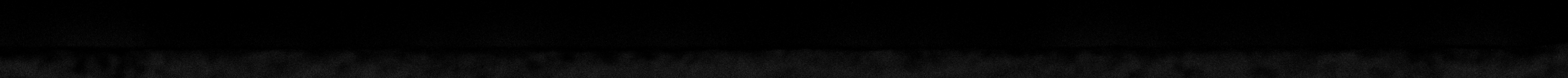

Supplement: Supplementary file 4 — Source data Fig. 3 [file 44321_2025_319_MOESM4_ESM.zip › Figure 3/Panel A/iRBC_Egress_Media_only_permeability_analysis_timepoint_masks/PC64_12_Top_RM_slice_9.tif]

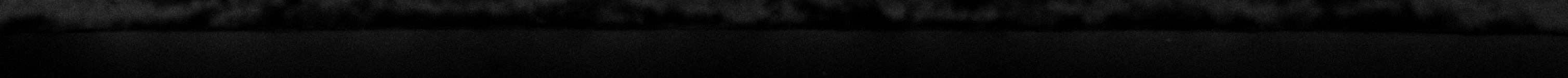

Supplement: Supplementary file 4 — Source data Fig. 3 [file 44321_2025_319_MOESM4_ESM.zip › Figure 3/Panel A/iRBC_Egress_Media_only_permeability_analysis_timepoint_masks/PC64_5_Bottom_RM_slice_18.tif]

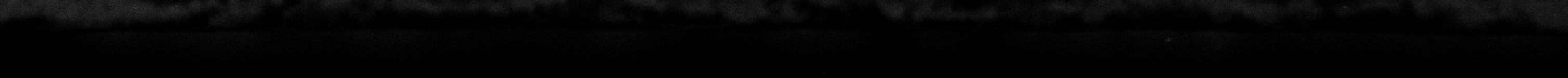

Supplement: Supplementary file 4 — Source data Fig. 3 [file 44321_2025_319_MOESM4_ESM.zip › Figure 3/Panel A/iRBC_Egress_Media_only_permeability_analysis_timepoint_masks/PC64_5_Bottom_RM_slice_8.tif]

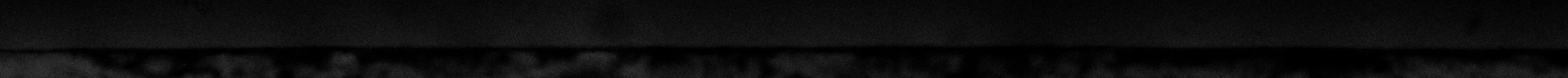

Supplement: Supplementary file 4 — Source data Fig. 3 [file 44321_2025_319_MOESM4_ESM.zip › Figure 3/Panel A/iRBC_Egress_Media_only_permeability_analysis_timepoint_masks/PC64_5_Top_RM_slice_18.tif]

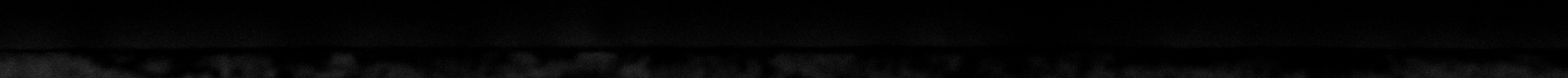

Supplement: Supplementary file 4 — Source data Fig. 3 [file 44321_2025_319_MOESM4_ESM.zip › Figure 3/Panel A/iRBC_Egress_Media_only_permeability_analysis_timepoint_masks/PC64_5_Top_RM_slice_8.tif]

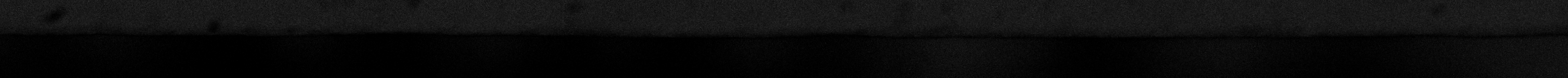

Supplement: Supplementary file 4 — Source data Fig. 3 [file 44321_2025_319_MOESM4_ESM.zip › Figure 3/Panel A/Media_only_permeability_analysis_timepoint_masks/PC62_3_Bottom_SM_slice_12.tif]

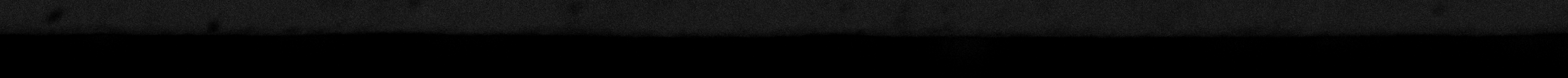

Supplement: Supplementary file 4 — Source data Fig. 3 [file 44321_2025_319_MOESM4_ESM.zip › Figure 3/Panel A/Media_only_permeability_analysis_timepoint_masks/PC62_3_Bottom_SM_slice_2.tif]

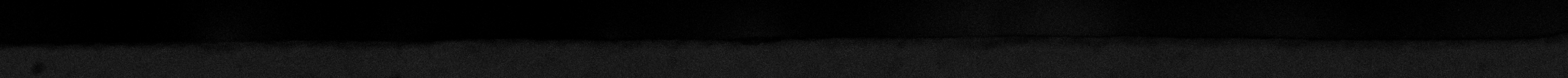

Supplement: Supplementary file 4 — Source data Fig. 3 [file 44321_2025_319_MOESM4_ESM.zip › Figure 3/Panel A/Media_only_permeability_analysis_timepoint_masks/PC62_3_Top_SM_slice_12.tif]

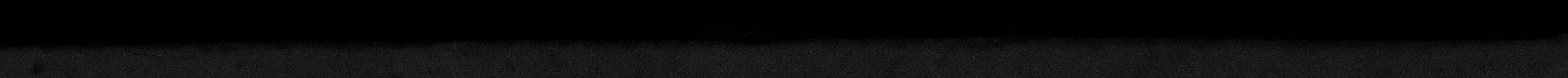

Supplement: Supplementary file 4 — Source data Fig. 3 [file 44321_2025_319_MOESM4_ESM.zip › Figure 3/Panel A/Media_only_permeability_analysis_timepoint_masks/PC62_3_Top_SM_slice_2.tif]

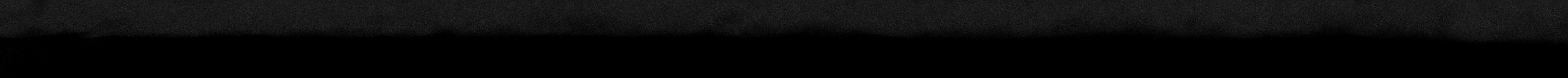

Supplement: Supplementary file 4 — Source data Fig. 3 [file 44321_2025_319_MOESM4_ESM.zip › Figure 3/Panel A/Media_only_permeability_analysis_timepoint_masks/PC62_4_Bottom_SM_slice_12.tif]

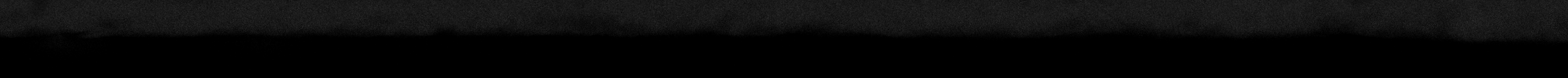

Supplement: Supplementary file 4 — Source data Fig. 3 [file 44321_2025_319_MOESM4_ESM.zip › Figure 3/Panel A/Media_only_permeability_analysis_timepoint_masks/PC62_4_Bottom_SM_slice_2.tif]

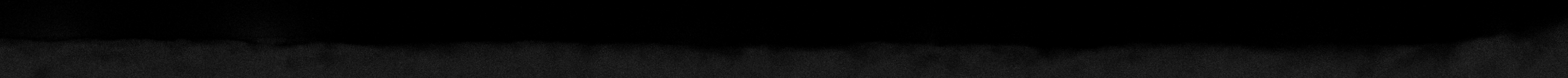

Supplement: Supplementary file 4 — Source data Fig. 3 [file 44321_2025_319_MOESM4_ESM.zip › Figure 3/Panel A/Media_only_permeability_analysis_timepoint_masks/PC62_4_Top_SM_slice_12.tif]

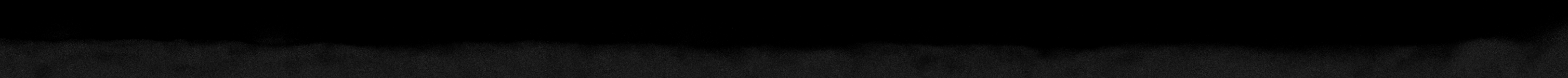

Supplement: Supplementary file 4 — Source data Fig. 3 [file 44321_2025_319_MOESM4_ESM.zip › Figure 3/Panel A/Media_only_permeability_analysis_timepoint_masks/PC62_4_Top_SM_slice_2.tif]

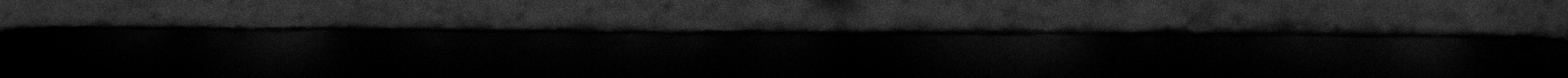

Supplement: Supplementary file 4 — Source data Fig. 3 [file 44321_2025_319_MOESM4_ESM.zip › Figure 3/Panel A/Media_only_permeability_analysis_timepoint_masks/PC63_10_Bottom_SM_slice_10.tif]

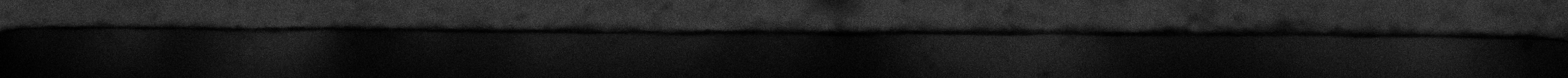

Supplement: Supplementary file 4 — Source data Fig. 3 [file 44321_2025_319_MOESM4_ESM.zip › Figure 3/Panel A/Media_only_permeability_analysis_timepoint_masks/PC63_10_Bottom_SM_slice_20.tif]

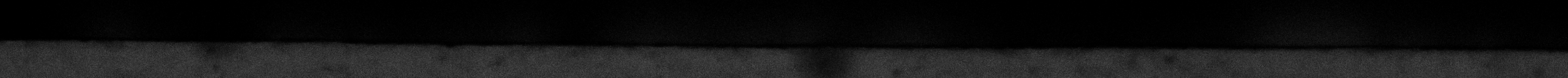

Supplement: Supplementary file 4 — Source data Fig. 3 [file 44321_2025_319_MOESM4_ESM.zip › Figure 3/Panel A/Media_only_permeability_analysis_timepoint_masks/PC63_10_Top_SM_slice_10.tif]

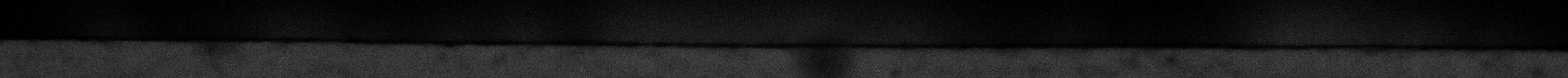

Supplement: Supplementary file 4 — Source data Fig. 3 [file 44321_2025_319_MOESM4_ESM.zip › Figure 3/Panel A/Media_only_permeability_analysis_timepoint_masks/PC63_10_Top_SM_slice_20.tif]

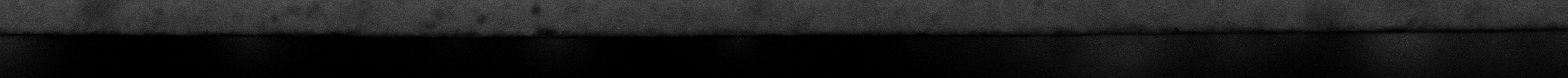

Supplement: Supplementary file 4 — Source data Fig. 3 [file 44321_2025_319_MOESM4_ESM.zip › Figure 3/Panel A/Media_only_permeability_analysis_timepoint_masks/PC63_12_Bottom_SM_slice_10.tif]

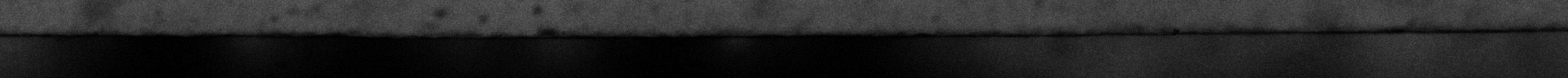

Supplement: Supplementary file 4 — Source data Fig. 3 [file 44321_2025_319_MOESM4_ESM.zip › Figure 3/Panel A/Media_only_permeability_analysis_timepoint_masks/PC63_12_Bottom_SM_slice_20.tif]

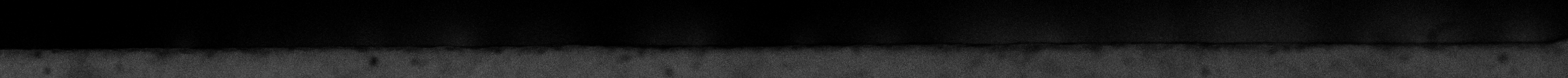

Supplement: Supplementary file 4 — Source data Fig. 3 [file 44321_2025_319_MOESM4_ESM.zip › Figure 3/Panel A/Media_only_permeability_analysis_timepoint_masks/PC63_12_Top_SM_slice_10.tif]

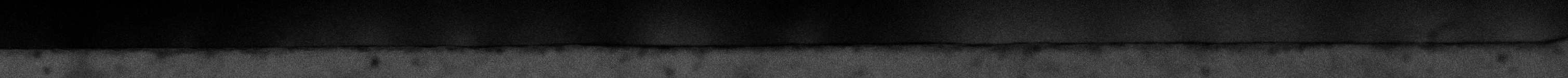

Supplement: Supplementary file 4 — Source data Fig. 3 [file 44321_2025_319_MOESM4_ESM.zip › Figure 3/Panel A/Media_only_permeability_analysis_timepoint_masks/PC63_12_Top_SM_slice_20.tif]

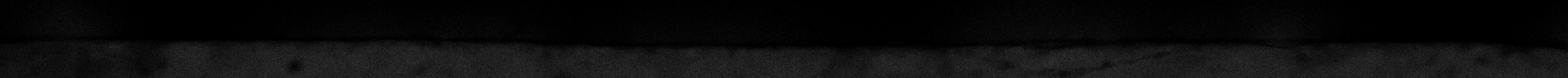

Supplement: Supplementary file 4 — Source data Fig. 3 [file 44321_2025_319_MOESM4_ESM.zip › Figure 3/Panel A/Media_only_permeability_analysis_timepoint_masks/PC63_3_Top_SM_slice_12.tif]

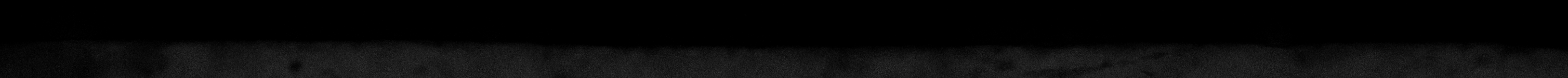

Supplement: Supplementary file 4 — Source data Fig. 3 [file 44321_2025_319_MOESM4_ESM.zip › Figure 3/Panel A/Media_only_permeability_analysis_timepoint_masks/PC63_3_Top_SM_slice_2.tif]

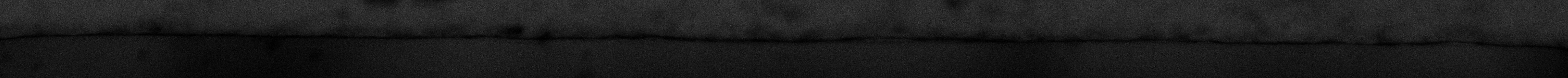

Supplement: Supplementary file 4 — Source data Fig. 3 [file 44321_2025_319_MOESM4_ESM.zip › Figure 3/Panel A/Media_only_permeability_analysis_timepoint_masks/PC63_5_Bottom_SM_slice_10.tif]

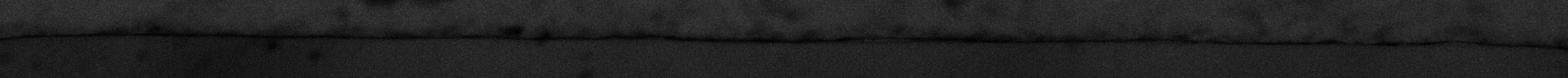

Supplement: Supplementary file 4 — Source data Fig. 3 [file 44321_2025_319_MOESM4_ESM.zip › Figure 3/Panel A/Media_only_permeability_analysis_timepoint_masks/PC63_5_Bottom_SM_slice_20.tif]

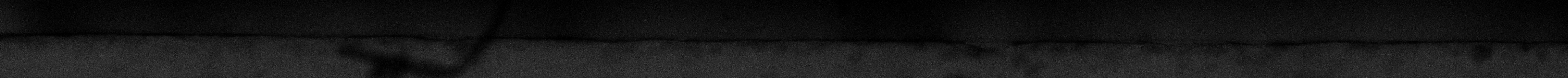

Supplement: Supplementary file 4 — Source data Fig. 3 [file 44321_2025_319_MOESM4_ESM.zip › Figure 3/Panel A/Media_only_permeability_analysis_timepoint_masks/PC63_5_Top_SM_slice_10.tif]

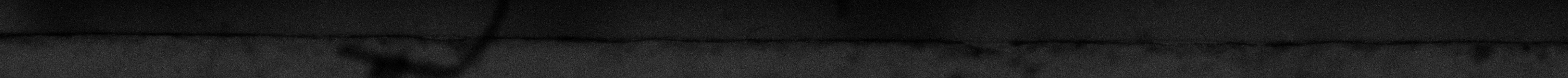

Supplement: Supplementary file 4 — Source data Fig. 3 [file 44321_2025_319_MOESM4_ESM.zip › Figure 3/Panel A/Media_only_permeability_analysis_timepoint_masks/PC63_5_Top_SM_slice_20.tif]

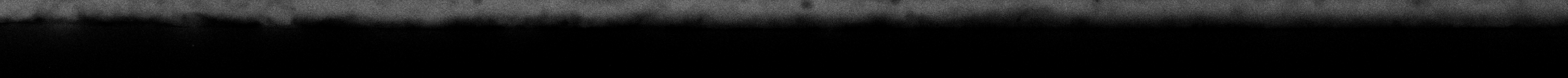

Supplement: Supplementary file 4 — Source data Fig. 3 [file 44321_2025_319_MOESM4_ESM.zip › Figure 3/Panel A/Media_only_permeability_analysis_timepoint_masks/PC64_1_Bottom_SM_slice_17.tif]

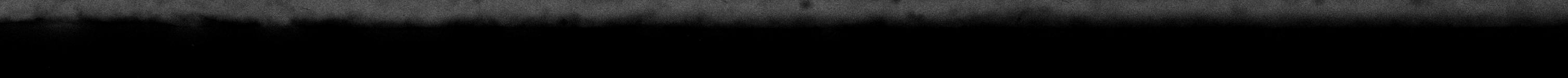

Supplement: Supplementary file 4 — Source data Fig. 3 [file 44321_2025_319_MOESM4_ESM.zip › Figure 3/Panel A/Media_only_permeability_analysis_timepoint_masks/PC64_1_Bottom_SM_slice_7.tif]

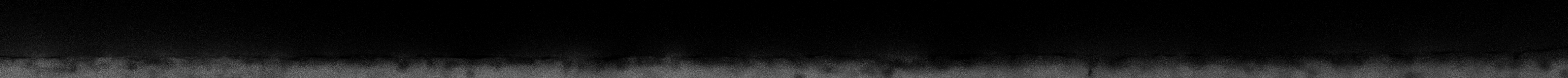

Supplement: Supplementary file 4 — Source data Fig. 3 [file 44321_2025_319_MOESM4_ESM.zip › Figure 3/Panel A/Media_only_permeability_analysis_timepoint_masks/PC64_1_Top_SM_slice_17.tif]

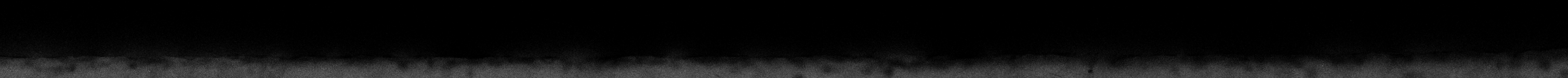

Supplement: Supplementary file 4 — Source data Fig. 3 [file 44321_2025_319_MOESM4_ESM.zip › Figure 3/Panel A/Media_only_permeability_analysis_timepoint_masks/PC64_1_Top_SM_slice_7.tif]

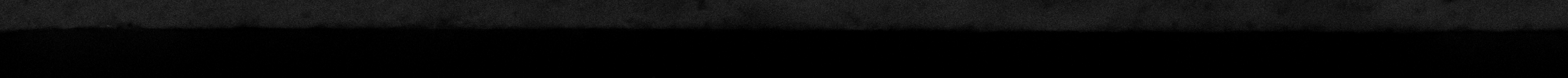

Supplement: Supplementary file 4 — Source data Fig. 3 [file 44321_2025_319_MOESM4_ESM.zip › Figure 3/Panel A/Media_only_permeability_analysis_timepoint_masks/PC64_10_Bottom_SM_slice_14.tif]

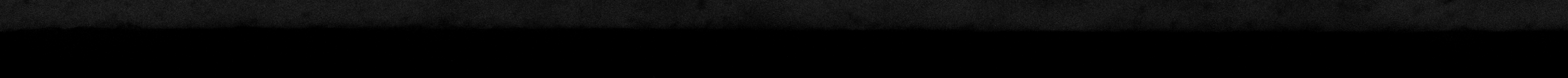

Supplement: Supplementary file 4 — Source data Fig. 3 [file 44321_2025_319_MOESM4_ESM.zip › Figure 3/Panel A/Media_only_permeability_analysis_timepoint_masks/PC64_10_Bottom_SM_slice_4.tif]

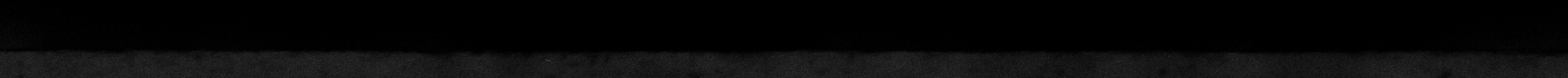

Supplement: Supplementary file 4 — Source data Fig. 3 [file 44321_2025_319_MOESM4_ESM.zip › Figure 3/Panel A/Media_only_permeability_analysis_timepoint_masks/PC64_10_Top_SM_slice_14.tif]

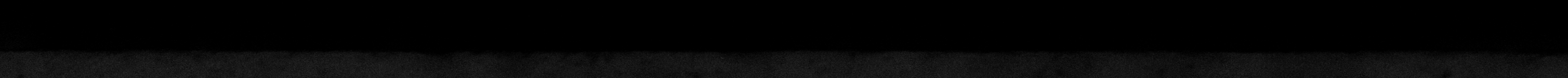

Supplement: Supplementary file 4 — Source data Fig. 3 [file 44321_2025_319_MOESM4_ESM.zip › Figure 3/Panel A/Media_only_permeability_analysis_timepoint_masks/PC64_10_Top_SM_slice_4.tif]

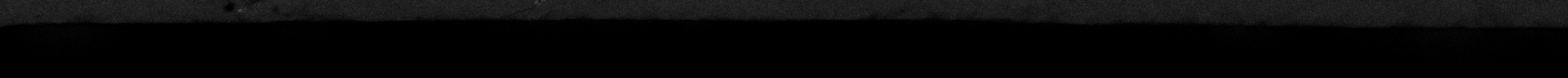

Supplement: Supplementary file 4 — Source data Fig. 3 [file 44321_2025_319_MOESM4_ESM.zip › Figure 3/Panel A/Media_only_permeability_analysis_timepoint_masks/PC64_8_Bottom_SM_slice_13.tif]

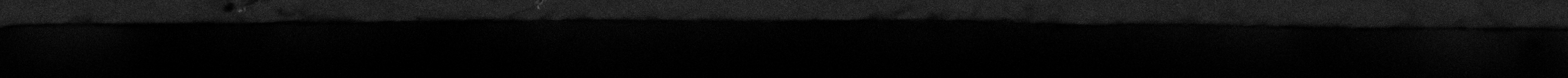

Supplement: Supplementary file 4 — Source data Fig. 3 [file 44321_2025_319_MOESM4_ESM.zip › Figure 3/Panel A/Media_only_permeability_analysis_timepoint_masks/PC64_8_Bottom_SM_slice_23.tif]

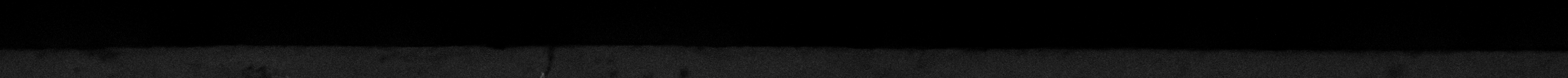

Supplement: Supplementary file 4 — Source data Fig. 3 [file 44321_2025_319_MOESM4_ESM.zip › Figure 3/Panel A/Media_only_permeability_analysis_timepoint_masks/PC64_8_Top_SM_slice_13.tif]

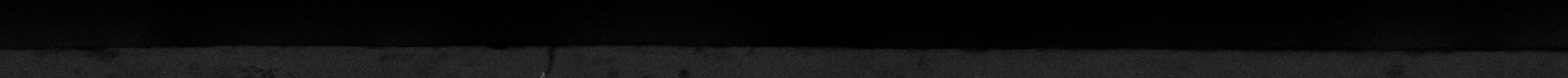

Supplement: Supplementary file 4 — Source data Fig. 3 [file 44321_2025_319_MOESM4_ESM.zip › Figure 3/Panel A/Media_only_permeability_analysis_timepoint_masks/PC64_8_Top_SM_slice_23.tif]

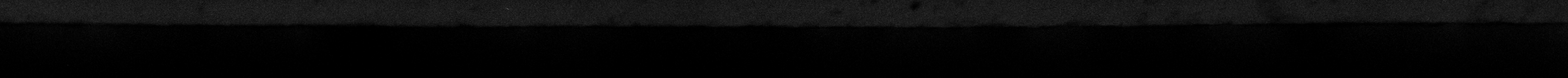

Supplement: Supplementary file 4 — Source data Fig. 3 [file 44321_2025_319_MOESM4_ESM.zip › Figure 3/Panel A/Media_only_permeability_analysis_timepoint_masks/PC64_9_Bottom_SM_slice_10.tif]

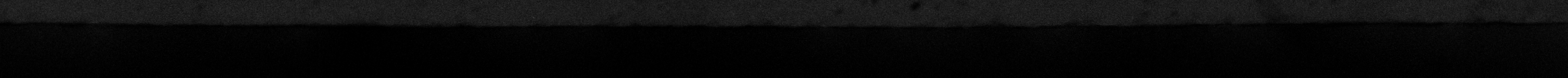

Supplement: Supplementary file 4 — Source data Fig. 3 [file 44321_2025_319_MOESM4_ESM.zip › Figure 3/Panel A/Media_only_permeability_analysis_timepoint_masks/PC64_9_Bottom_SM_slice_20.tif]

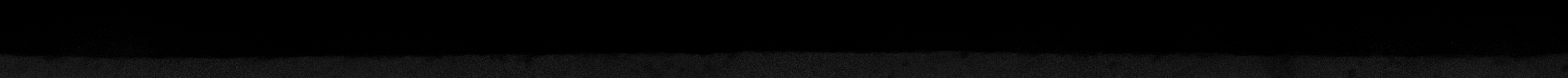

Supplement: Supplementary file 4 — Source data Fig. 3 [file 44321_2025_319_MOESM4_ESM.zip › Figure 3/Panel A/Media_only_permeability_analysis_timepoint_masks/PC64_9_Top_SM_slice_10.tif]

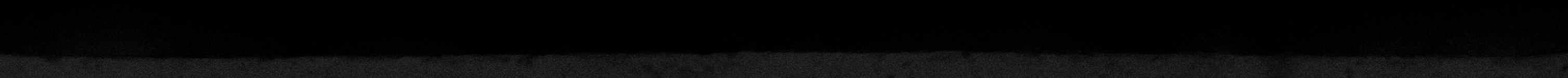

Supplement: Supplementary file 4 — Source data Fig. 3 [file 44321_2025_319_MOESM4_ESM.zip › Figure 3/Panel A/Media_only_permeability_analysis_timepoint_masks/PC64_9_Top_SM_slice_20.tif]

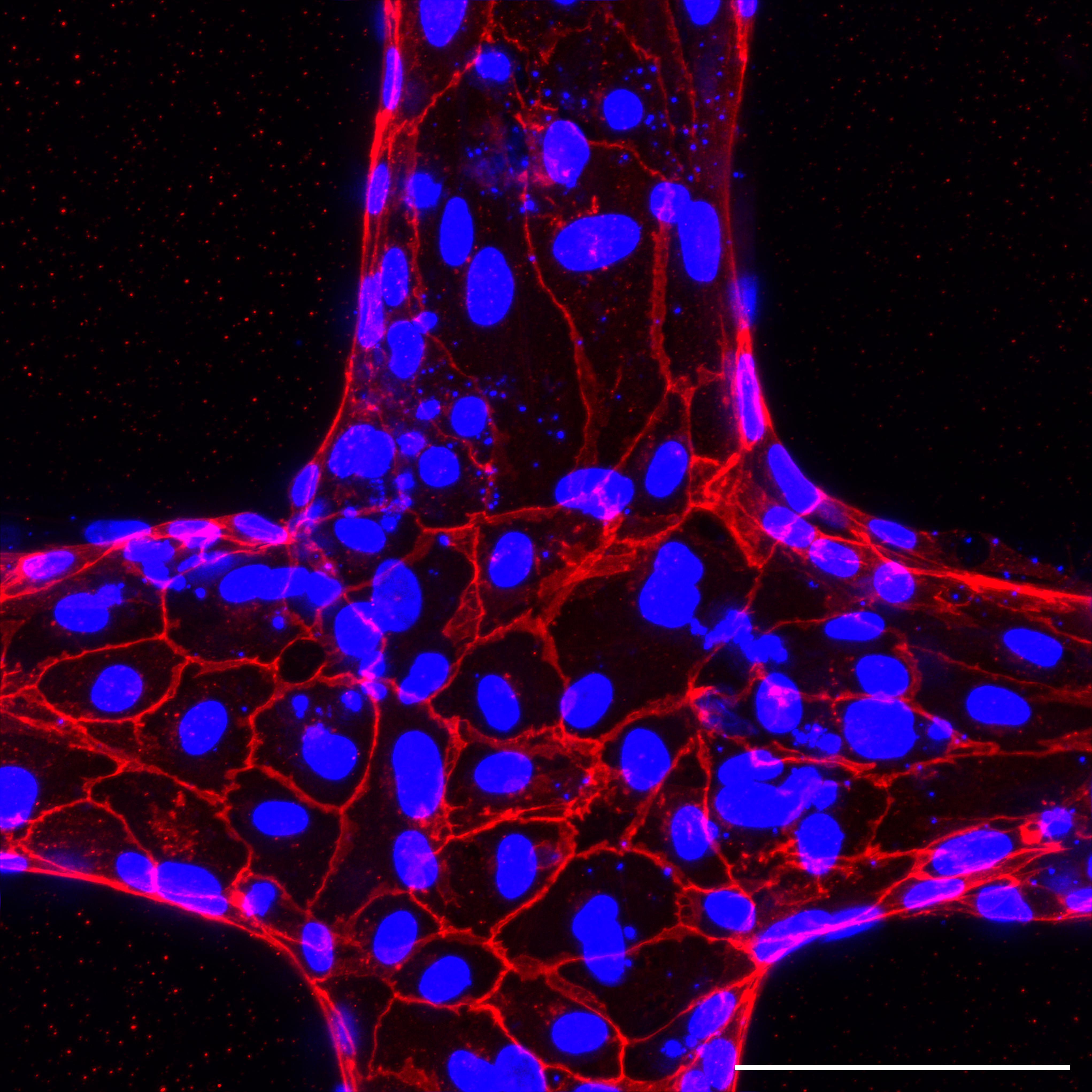

Supplement: Supplementary file 4 — Source data Fig. 3 [file 44321_2025_319_MOESM4_ESM.zip › Figure 3/Panel C/MAX_PC48_10_5_1_HBVP_HBMEC_iRBC_egress_media_VECAD_20550_DAPI._17500.tif]

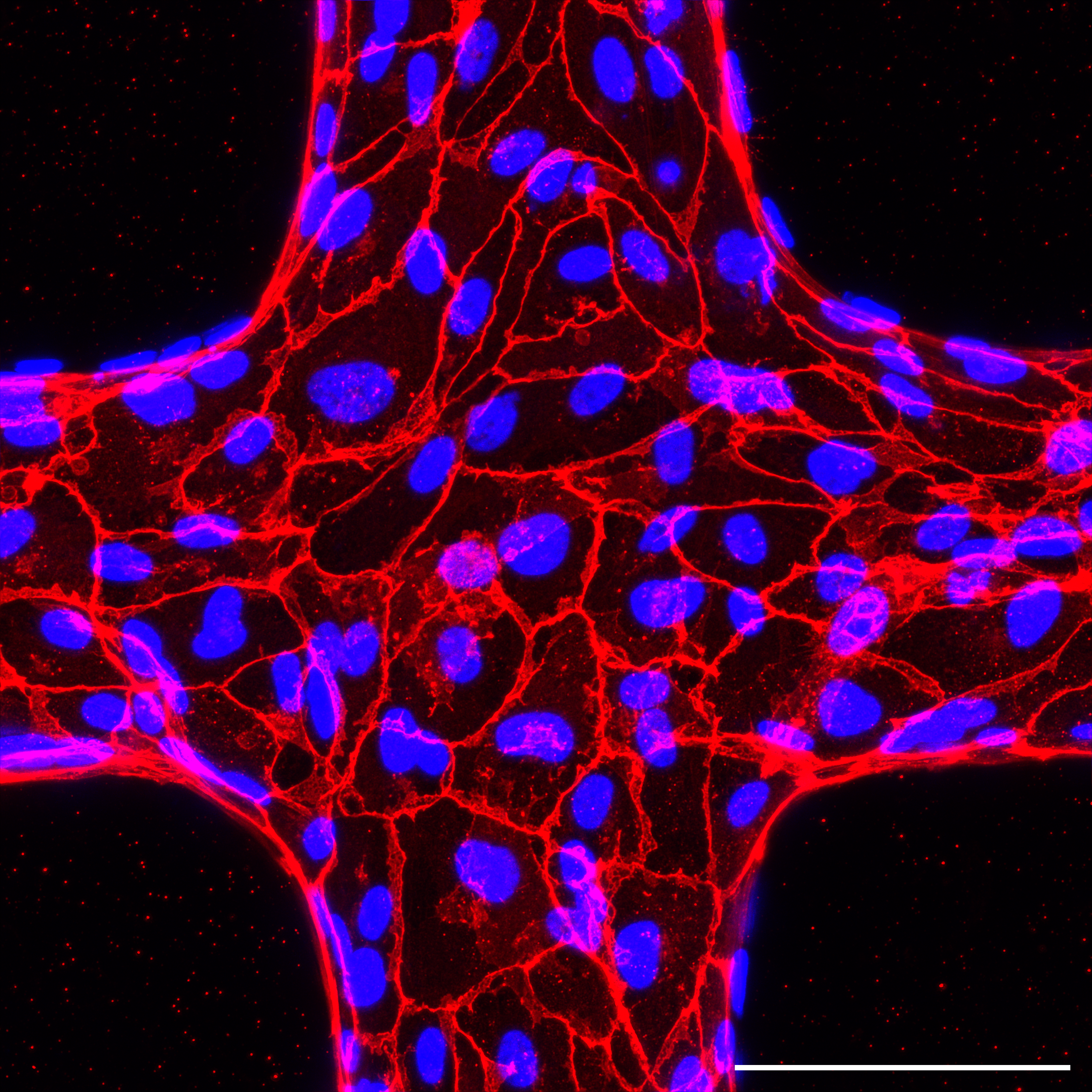

Supplement: Supplementary file 4 — Source data Fig. 3 [file 44321_2025_319_MOESM4_ESM.zip › Figure 3/Panel C/MAX_PC48_6_5_1_HBVP_HBMEC_media_only_VECAD_20550_DAPI_17500.tif]

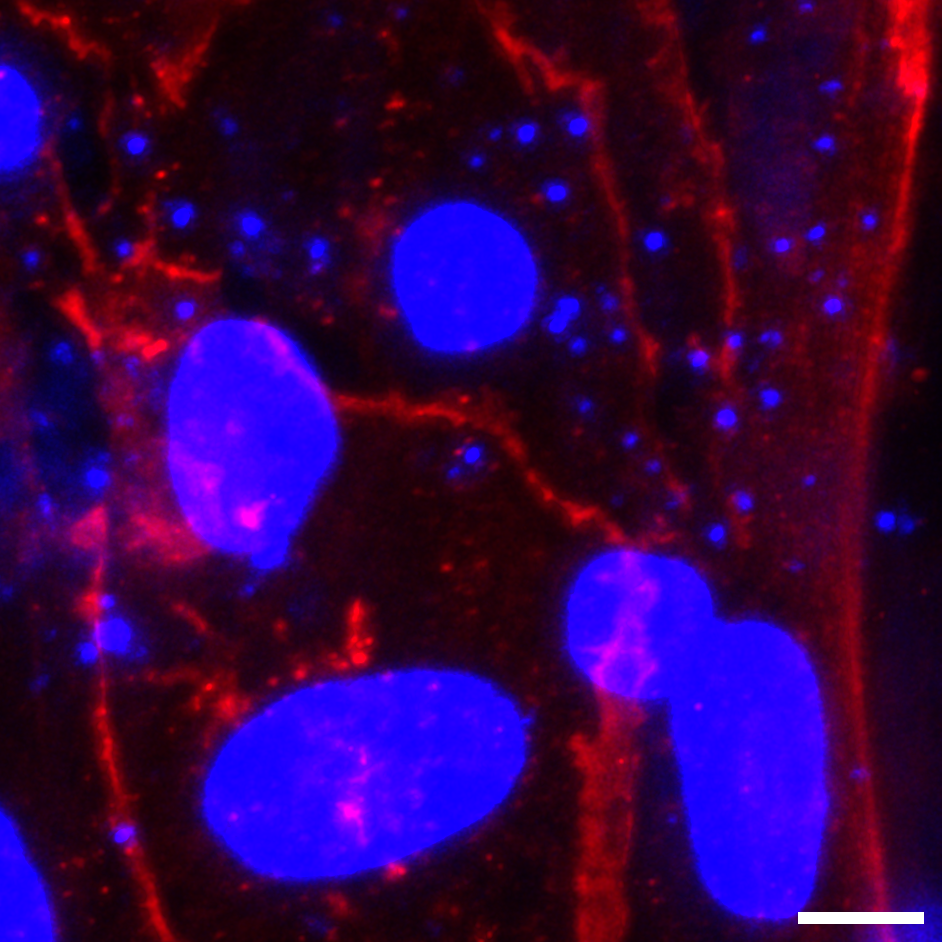

Supplement: Supplementary file 4 — Source data Fig. 3 [file 44321_2025_319_MOESM4_ESM.zip › Figure 3/Panel C/ROI_MAX_PC48_10_5_1_HBVP_HBMEC_iRBC_Egress_media_VECAD_20550_DAPI._17500_75_75um_Scale10um.tif]

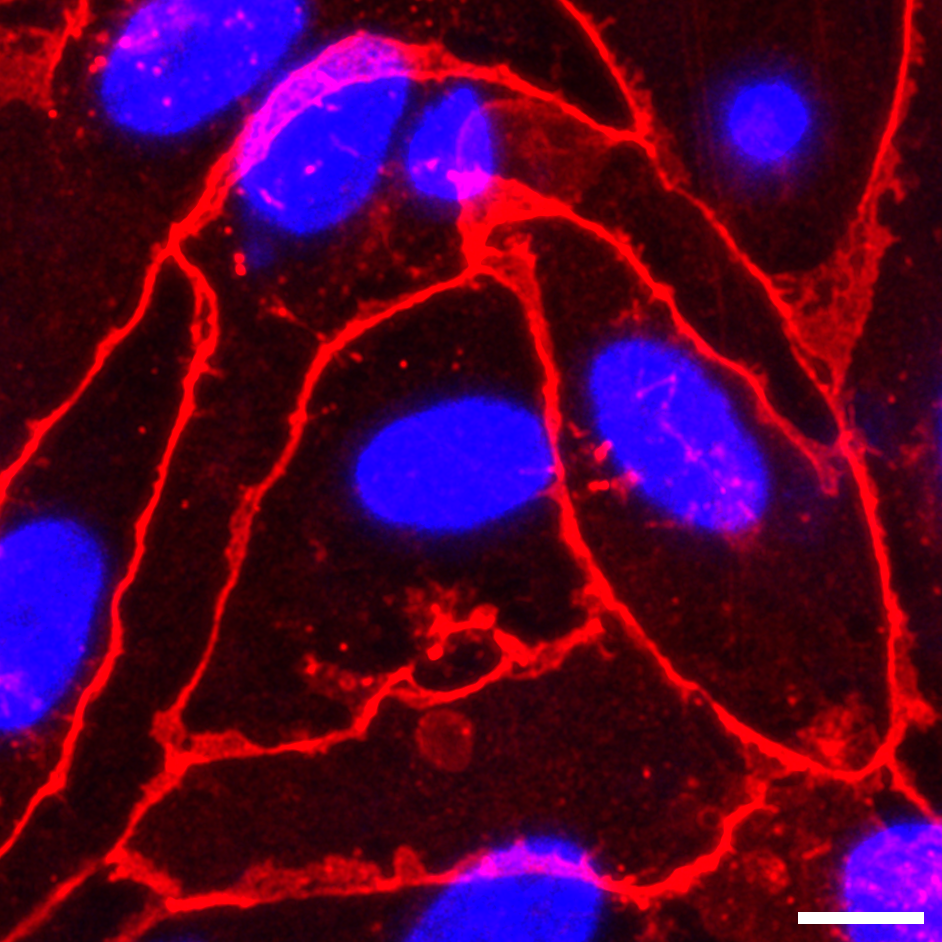

Supplement: Supplementary file 4 — Source data Fig. 3 [file 44321_2025_319_MOESM4_ESM.zip › Figure 3/Panel C/ROI_MAX_PC48_6_5_1_HBVP_HBMEC_media_only_VECAD_20550_DAPI_17500_75_75um_Scale10um.tif]

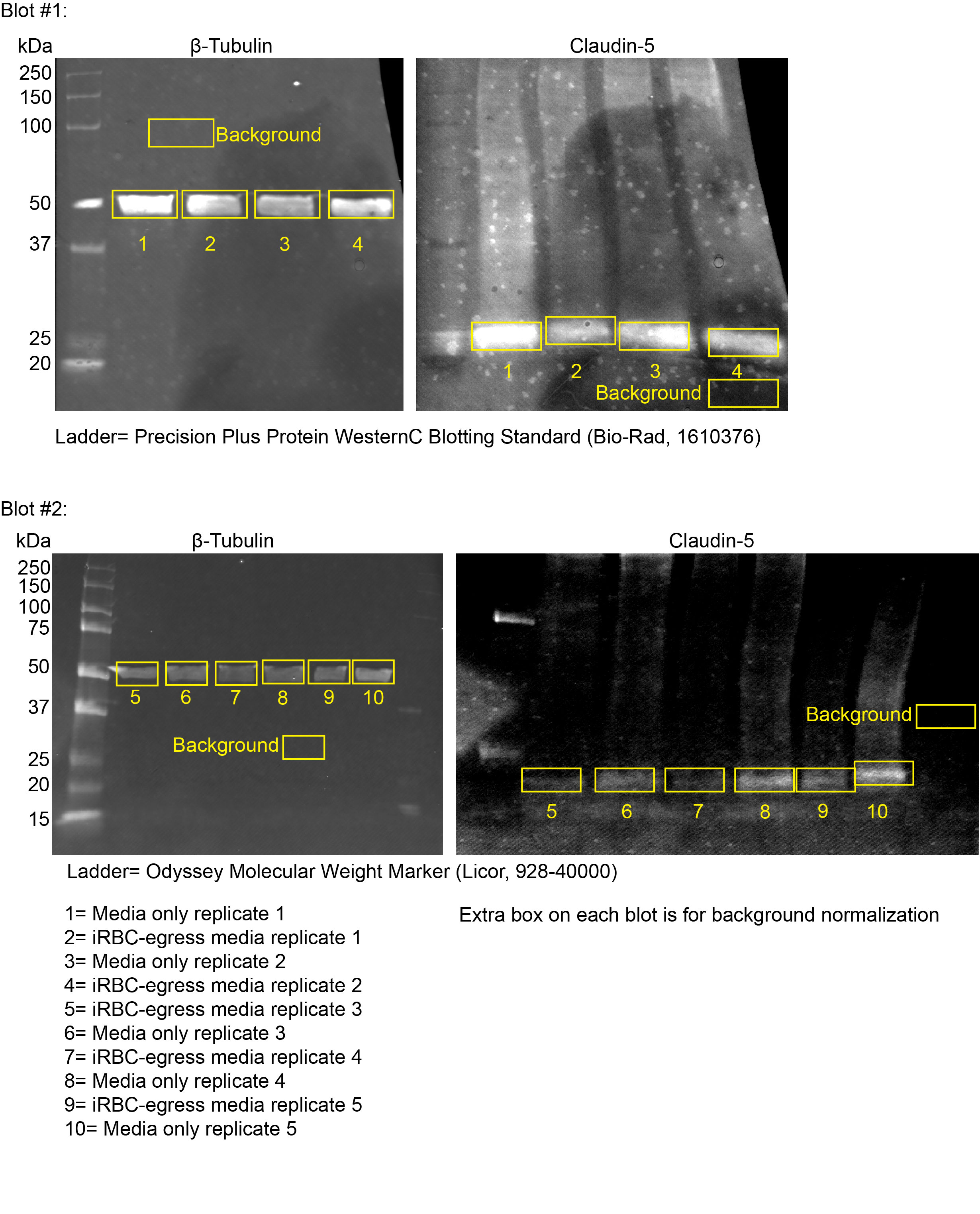

Supplement: Supplementary file 4 — Source data Fig. 3 [file 44321_2025_319_MOESM4_ESM.zip › Figure 3/Panel D/Claudin_5_Western_Blot_Raw_Images_with_annotation.jpg]

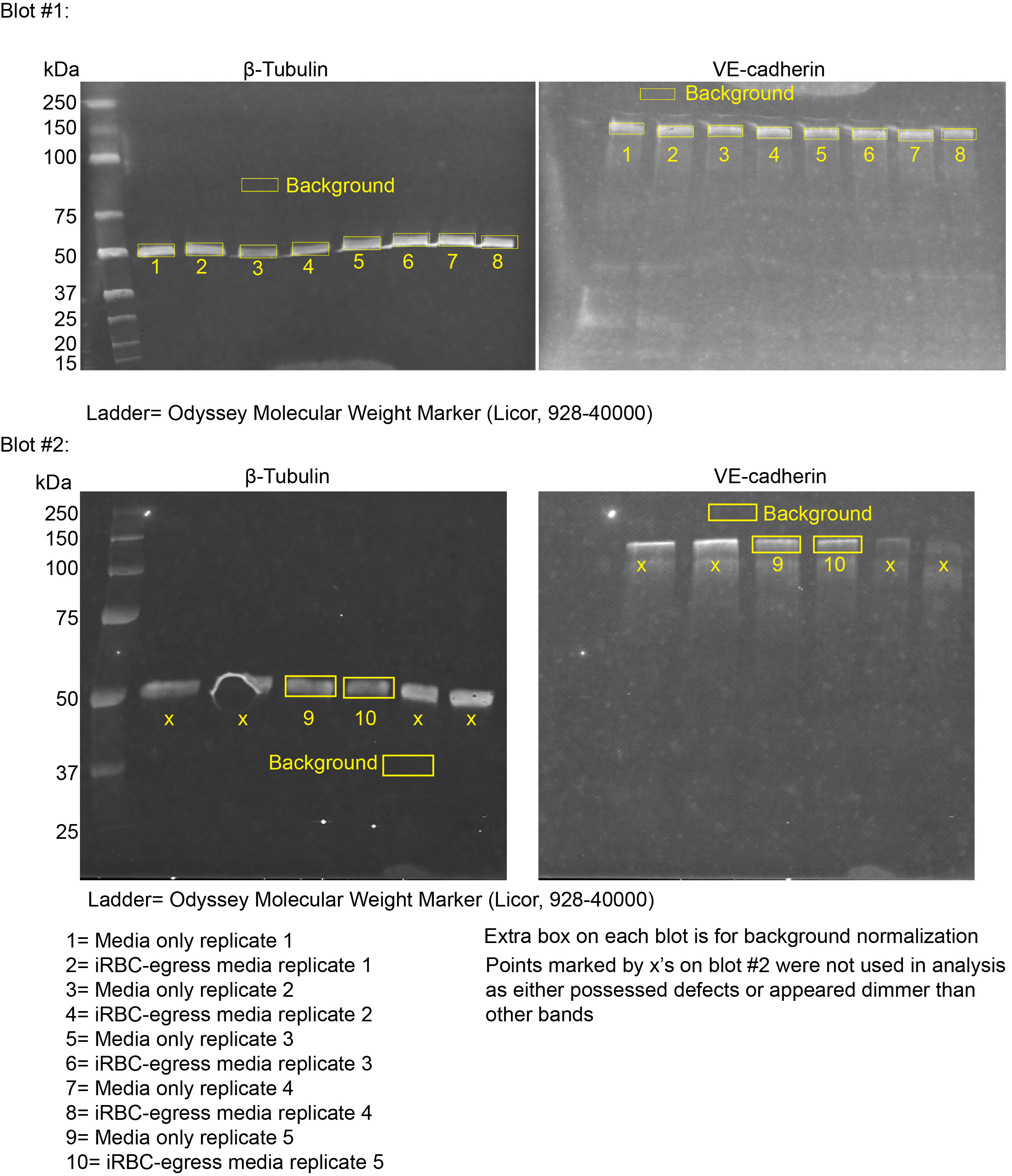

Supplement: Supplementary file 4 — Source data Fig. 3 [file 44321_2025_319_MOESM4_ESM.zip › Figure 3/Panel E/VE_Cadherin_Western_Blot_Raw_Images_with_annotation.jpg]

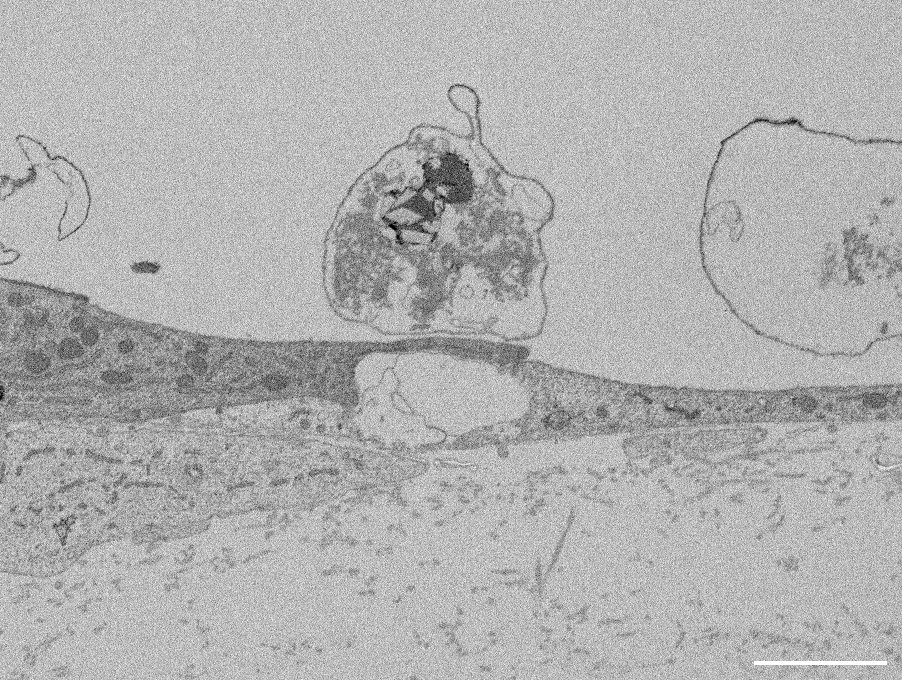

Supplement: Supplementary file 5 — Source data Fig. 4 [file 44321_2025_319_MOESM5_ESM.zip › Figure 4/Panel C/Egress_media_section_158_Ghost_Figure 4C Middle Image.jpg]

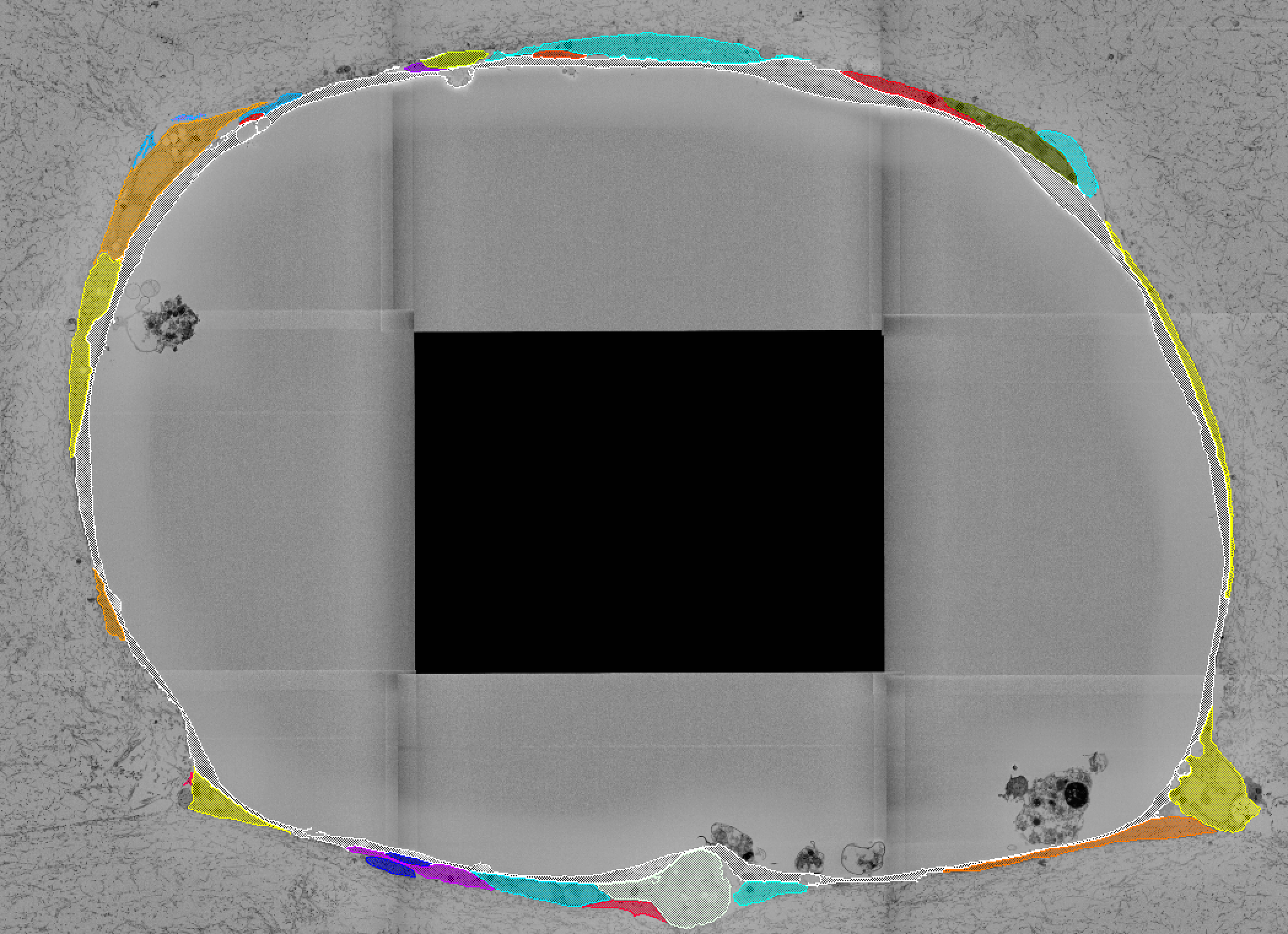

Supplement: Supplementary file 5 — Source data Fig. 4 [file 44321_2025_319_MOESM5_ESM.zip › Figure 4/Panel C/Screenshot_Egress_media_section_158_Pericyte_Endothelial_cell_Segmentation.PNG]

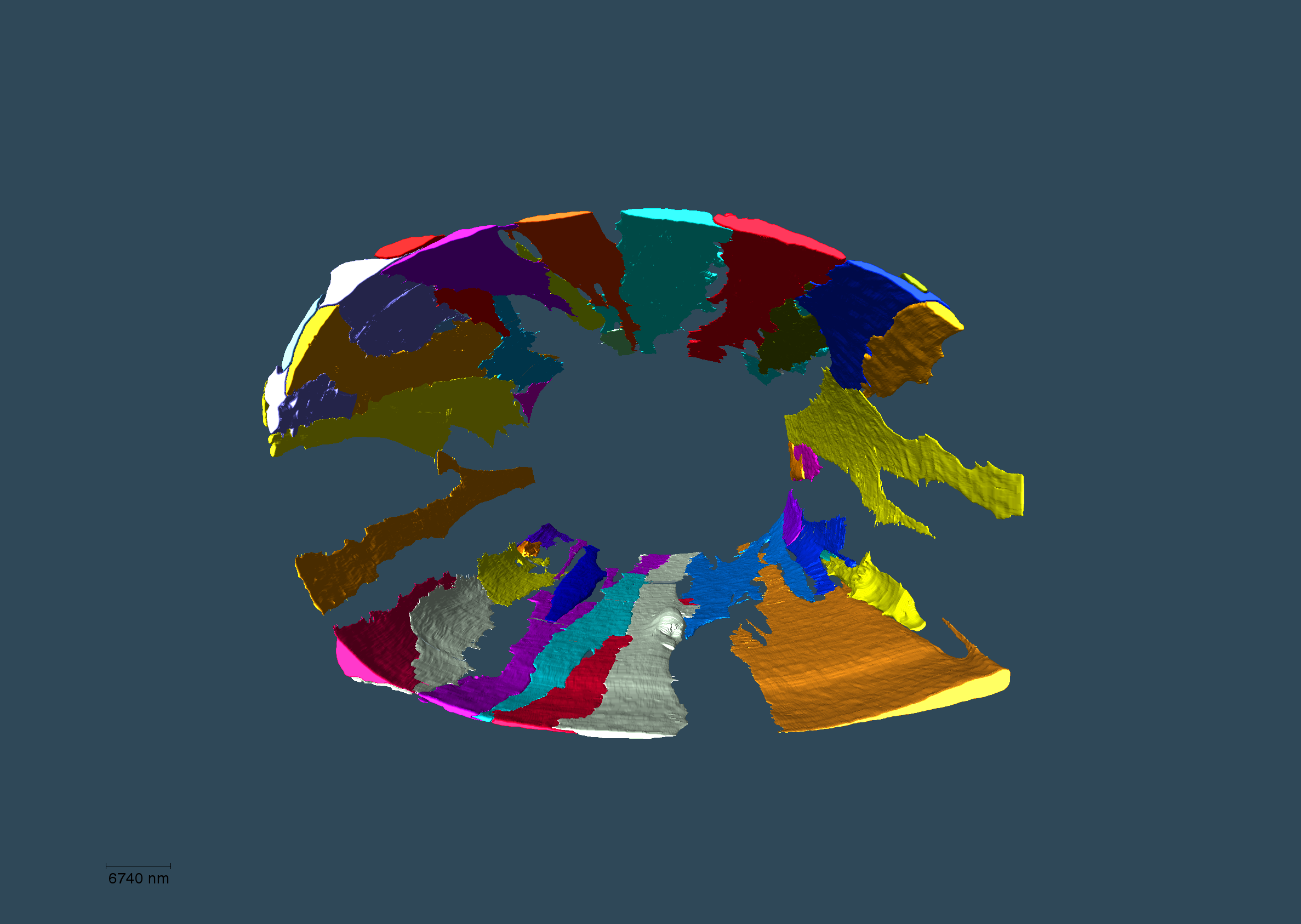

Supplement: Supplementary file 5 — Source data Fig. 4 [file 44321_2025_319_MOESM5_ESM.zip › Figure 4/Panel D/snapshot_Egress_media_crosssection_front_segmented_grey_background_scalebar_July102025_Contrast177.tif]

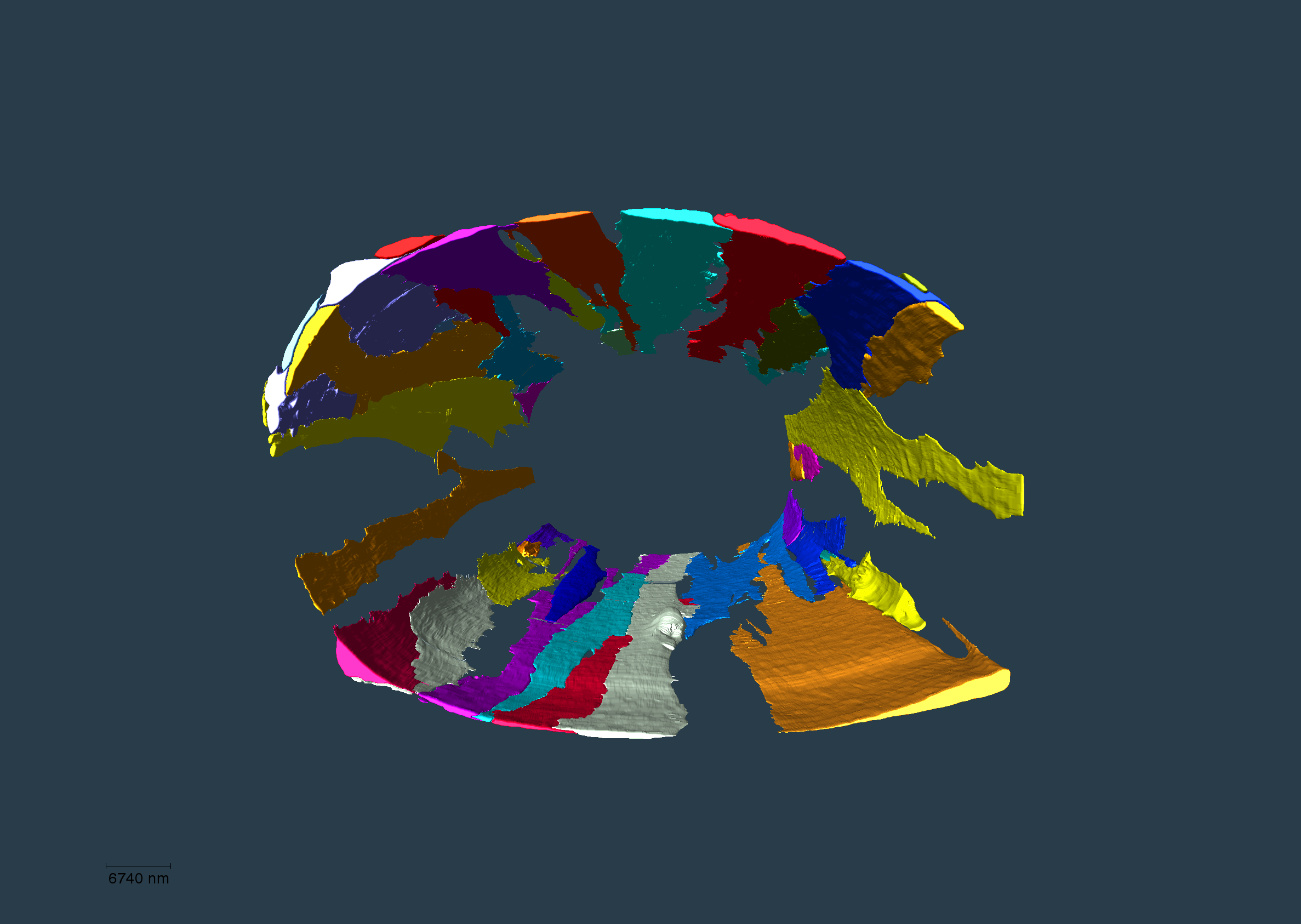

Supplement: Supplementary file 5 — Source data Fig. 4 [file 44321_2025_319_MOESM5_ESM.zip › Figure 4/Panel D/snapshot_Egress_media_crosssection_front_segmented_greyer_background_scalebar_July102025_Contrast177.tif]

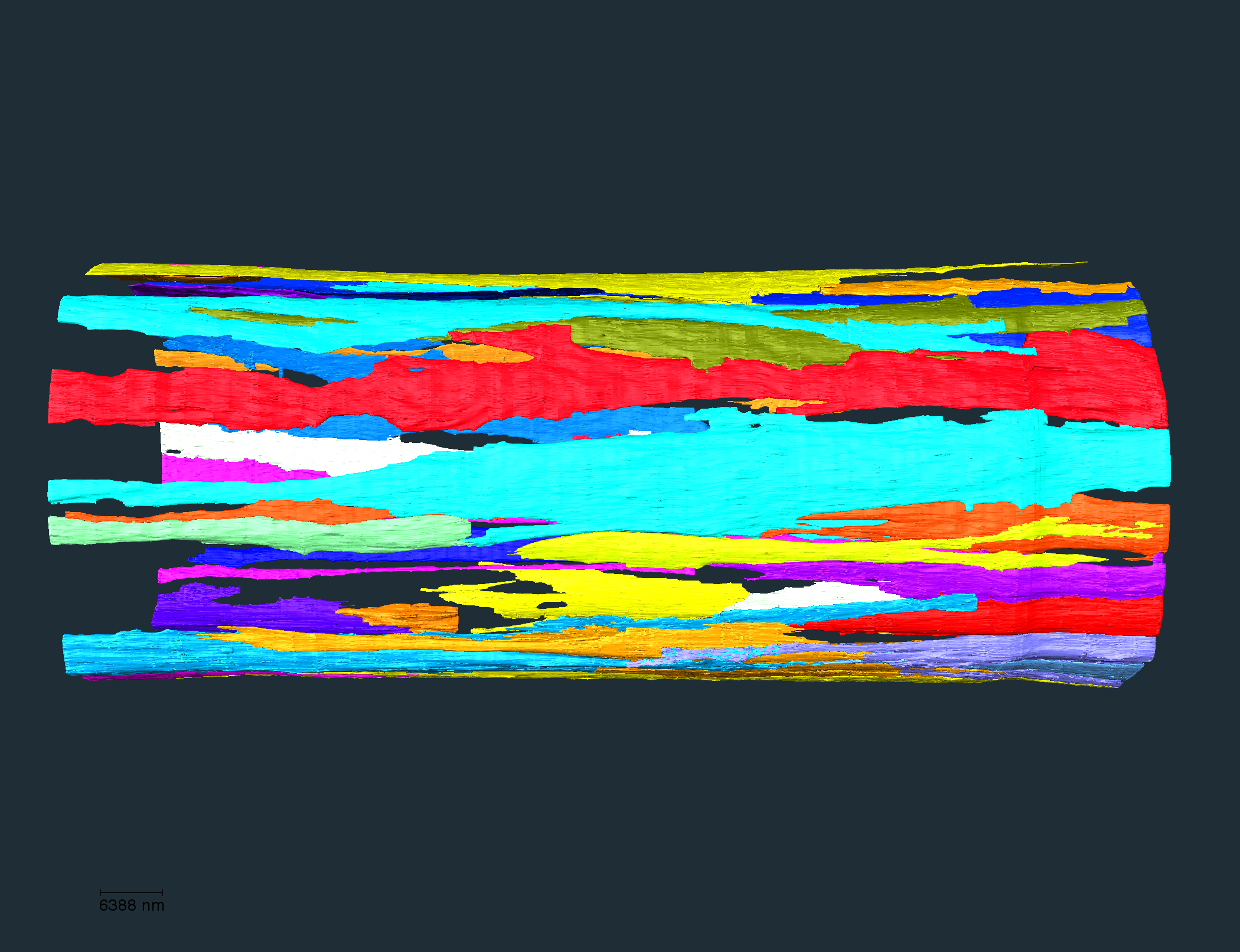

Supplement: Supplementary file 5 — Source data Fig. 4 [file 44321_2025_319_MOESM5_ESM.zip › Figure 4/Panel D/snapshot_Egress_media_top_segmented_grey_background_scalebar_July102025.png]

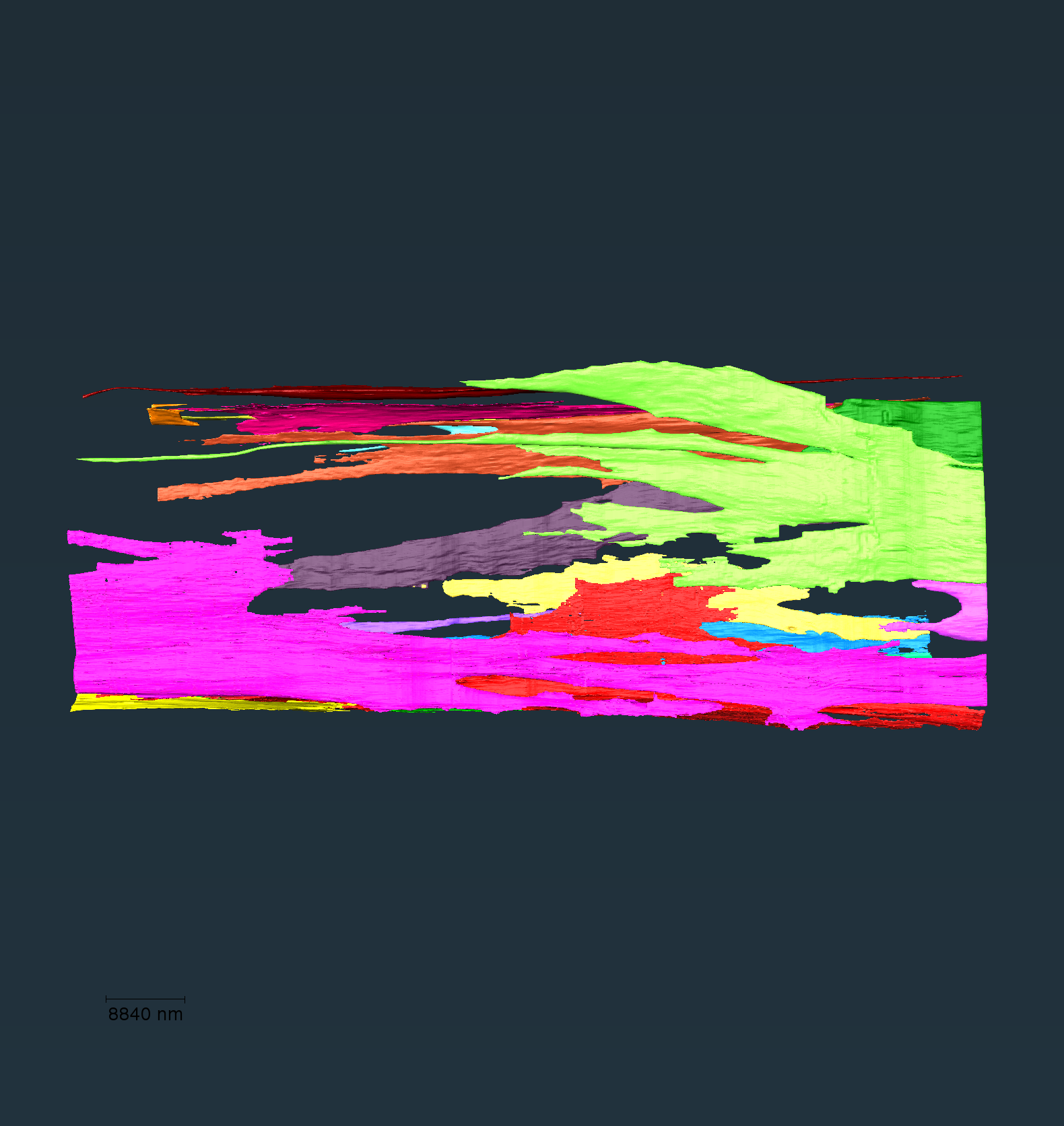

Supplement: Supplementary file 5 — Source data Fig. 4 [file 44321_2025_319_MOESM5_ESM.zip › Figure 4/Panel D/snapshot_Media_only_crosssection_Top_segmented_grey_background_scalebar_July112025.png]

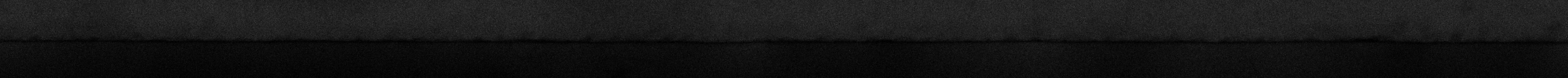

Supplement: Supplementary file 7 — Source data Fig. 6 [file 44321_2025_319_MOESM7_ESM.zip › Figure 6/Panel B/Permeability masks_time1_time2_used for analysis_rAng1/PC65_10_Bottom_SM_Ang1_slice_19.tif]

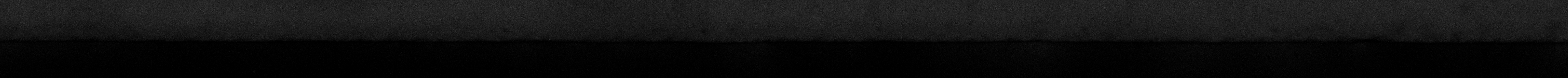

Supplement: Supplementary file 7 — Source data Fig. 6 [file 44321_2025_319_MOESM7_ESM.zip › Figure 6/Panel B/Permeability masks_time1_time2_used for analysis_rAng1/PC65_10_Bottom_SM_Ang1_slice_9.tif]

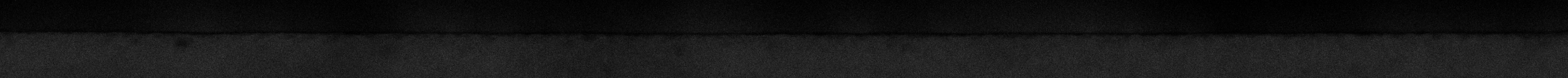

Supplement: Supplementary file 7 — Source data Fig. 6 [file 44321_2025_319_MOESM7_ESM.zip › Figure 6/Panel B/Permeability masks_time1_time2_used for analysis_rAng1/PC65_10_Top_SM_Ang1_slice_19.tif]

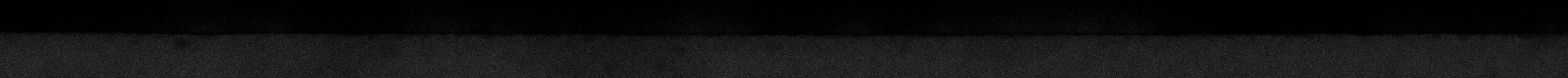

Supplement: Supplementary file 7 — Source data Fig. 6 [file 44321_2025_319_MOESM7_ESM.zip › Figure 6/Panel B/Permeability masks_time1_time2_used for analysis_rAng1/PC65_10_Top_SM_Ang1_slice_9.tif]

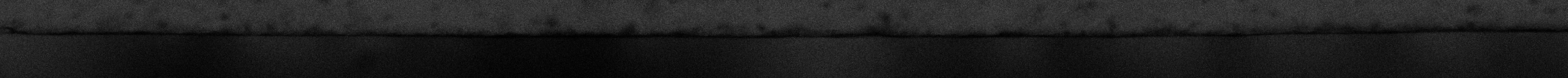

Supplement: Supplementary file 7 — Source data Fig. 6 [file 44321_2025_319_MOESM7_ESM.zip › Figure 6/Panel B/Permeability masks_time1_time2_used for analysis_rAng1/PC65_2_Bottom_SM_Ang1_slice_16.tif]

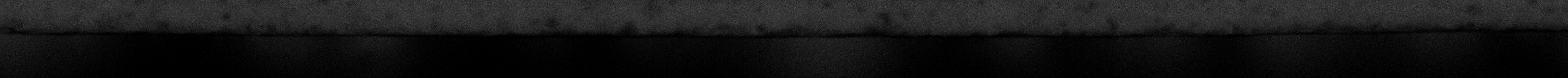

Supplement: Supplementary file 7 — Source data Fig. 6 [file 44321_2025_319_MOESM7_ESM.zip › Figure 6/Panel B/Permeability masks_time1_time2_used for analysis_rAng1/PC65_2_Bottom_SM_Ang1_slice_6.tif]

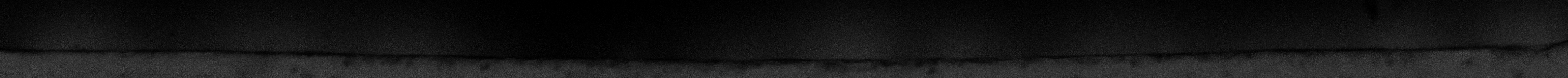

Supplement: Supplementary file 7 — Source data Fig. 6 [file 44321_2025_319_MOESM7_ESM.zip › Figure 6/Panel B/Permeability masks_time1_time2_used for analysis_rAng1/PC65_2_Top_SM_Ang1_slice_16.tif]

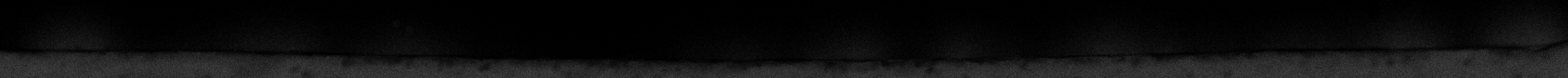

Supplement: Supplementary file 7 — Source data Fig. 6 [file 44321_2025_319_MOESM7_ESM.zip › Figure 6/Panel B/Permeability masks_time1_time2_used for analysis_rAng1/PC65_2_Top_SM_Ang1_slice_6.tif]

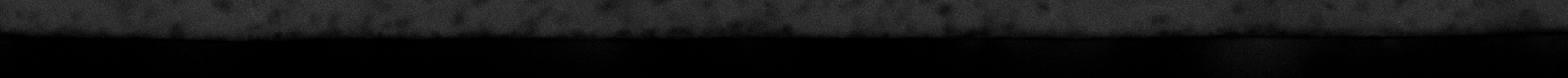

Supplement: Supplementary file 7 — Source data Fig. 6 [file 44321_2025_319_MOESM7_ESM.zip › Figure 6/Panel B/Permeability masks_time1_time2_used for analysis_rAng1/PC65_3_Bottom_SM_Ang1_slice_10.tif]

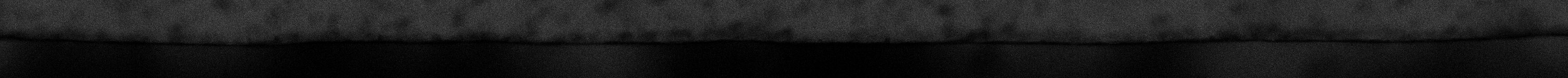

Supplement: Supplementary file 7 — Source data Fig. 6 [file 44321_2025_319_MOESM7_ESM.zip › Figure 6/Panel B/Permeability masks_time1_time2_used for analysis_rAng1/PC65_3_Bottom_SM_Ang1_slice_20.tif]

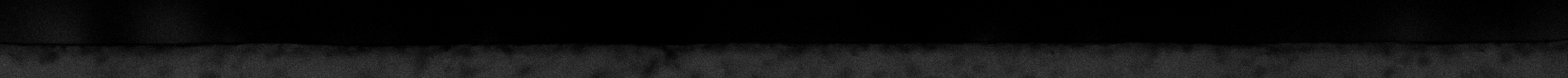

Supplement: Supplementary file 7 — Source data Fig. 6 [file 44321_2025_319_MOESM7_ESM.zip › Figure 6/Panel B/Permeability masks_time1_time2_used for analysis_rAng1/PC65_3_Top_SM_Ang1_slice_10.tif]

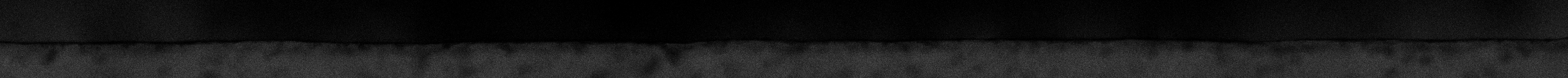

Supplement: Supplementary file 7 — Source data Fig. 6 [file 44321_2025_319_MOESM7_ESM.zip › Figure 6/Panel B/Permeability masks_time1_time2_used for analysis_rAng1/PC65_3_Top_SM_Ang1_slice_20.tif]

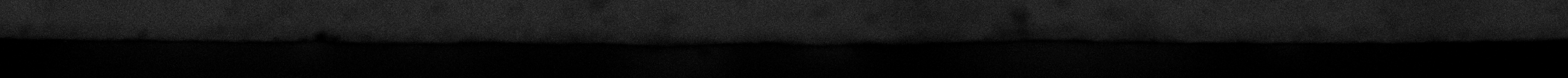

Supplement: Supplementary file 7 — Source data Fig. 6 [file 44321_2025_319_MOESM7_ESM.zip › Figure 6/Panel B/Permeability masks_time1_time2_used for analysis_rAng1/PC65_7_Bottom_SM_Ang1_slice_15.tif]

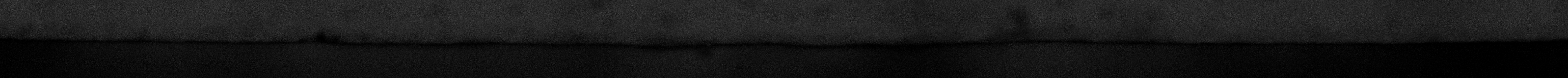

Supplement: Supplementary file 7 — Source data Fig. 6 [file 44321_2025_319_MOESM7_ESM.zip › Figure 6/Panel B/Permeability masks_time1_time2_used for analysis_rAng1/PC65_7_Bottom_SM_Ang1_slice_25.tif]

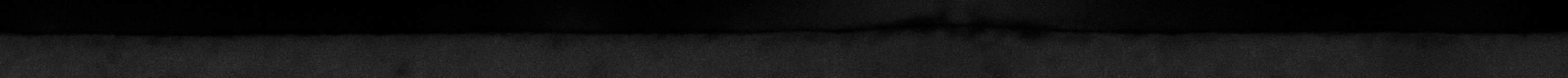

Supplement: Supplementary file 7 — Source data Fig. 6 [file 44321_2025_319_MOESM7_ESM.zip › Figure 6/Panel B/Permeability masks_time1_time2_used for analysis_rAng1/PC65_7_Top_SM_Ang1_slice_15.tif]

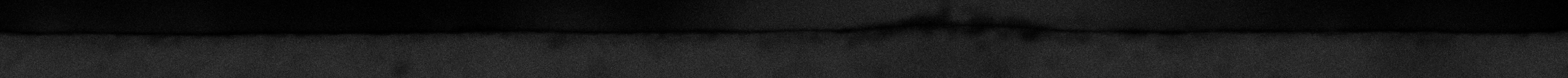

Supplement: Supplementary file 7 — Source data Fig. 6 [file 44321_2025_319_MOESM7_ESM.zip › Figure 6/Panel B/Permeability masks_time1_time2_used for analysis_rAng1/PC65_7_Top_SM_Ang1_slice_25.tif]

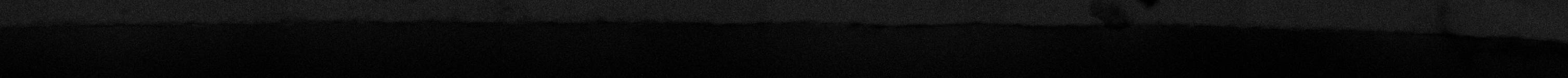

Supplement: Supplementary file 7 — Source data Fig. 6 [file 44321_2025_319_MOESM7_ESM.zip › Figure 6/Panel B/Permeability masks_time1_time2_used for analysis_rAng1/PC66_10_Bottom_SM_Ang1_slice_17.tif]

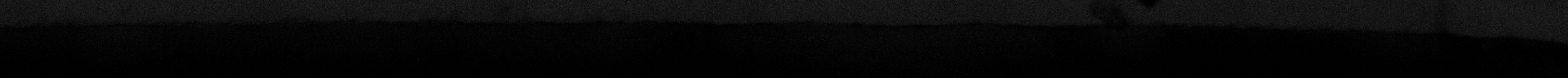

Supplement: Supplementary file 7 — Source data Fig. 6 [file 44321_2025_319_MOESM7_ESM.zip › Figure 6/Panel B/Permeability masks_time1_time2_used for analysis_rAng1/PC66_10_Bottom_SM_Ang1_slice_7.tif]
