# Supplementary figures and images for: Plasmodium falciparum impairs Ang-1 secretion by pericytes in a 3D brain microvessel model (part 4 of 4)
Source: EMBO Mol Med. 2025 Oct 16;17(11):3110–38. doi: 10.1038/s44321-025-00319-y (PMC12603187; doi:10.1038/s44321-025-00319-y)

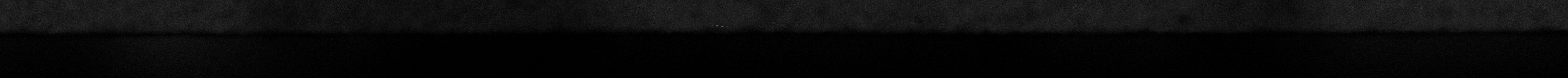

Supplement: Supplementary file 7 — Source data Fig. 6 [file 44321_2025_319_MOESM7_ESM.zip › Figure 6/Panel B/Permeability masks_time1_time2_used for analysis_Serum_free_media_only/PC68_6_Bottom_SM_SF_slice_12.tif]

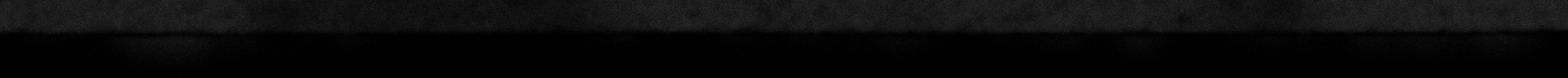

Supplement: Supplementary file 7 — Source data Fig. 6 [file 44321_2025_319_MOESM7_ESM.zip › Figure 6/Panel B/Permeability masks_time1_time2_used for analysis_Serum_free_media_only/PC68_6_Bottom_SM_SF_slice_2.tif]

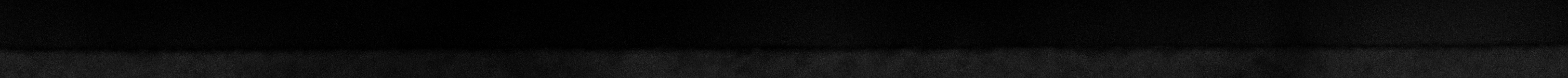

Supplement: Supplementary file 7 — Source data Fig. 6 [file 44321_2025_319_MOESM7_ESM.zip › Figure 6/Panel B/Permeability masks_time1_time2_used for analysis_Serum_free_media_only/PC68_6_Top_SM_SF_slice_12.tif]

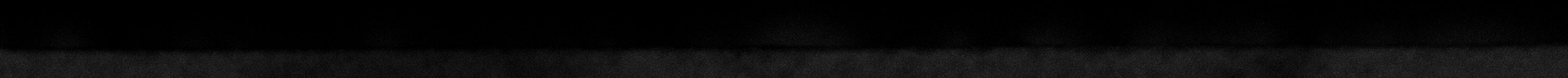

Supplement: Supplementary file 7 — Source data Fig. 6 [file 44321_2025_319_MOESM7_ESM.zip › Figure 6/Panel B/Permeability masks_time1_time2_used for analysis_Serum_free_media_only/PC68_6_Top_SM_SF_slice_2.tif]

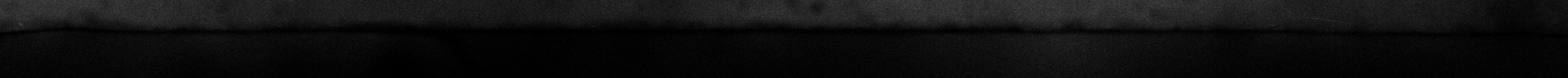

Supplement: Supplementary file 7 — Source data Fig. 6 [file 44321_2025_319_MOESM7_ESM.zip › Figure 6/Panel B/Permeability masks_time1_time2_used for analysis_Serum_free_media_only/PC71_7_Bottom_SM_SF_slice_18.tif]

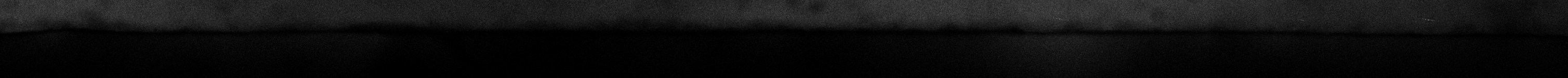

Supplement: Supplementary file 7 — Source data Fig. 6 [file 44321_2025_319_MOESM7_ESM.zip › Figure 6/Panel B/Permeability masks_time1_time2_used for analysis_Serum_free_media_only/PC71_7_Bottom_SM_SF_slice_8.tif]

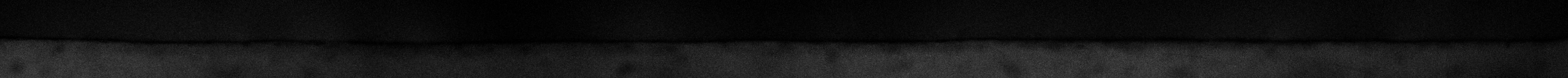

Supplement: Supplementary file 7 — Source data Fig. 6 [file 44321_2025_319_MOESM7_ESM.zip › Figure 6/Panel B/Permeability masks_time1_time2_used for analysis_Serum_free_media_only/PC71_7_Top_SM_SF_slice_18.tif]

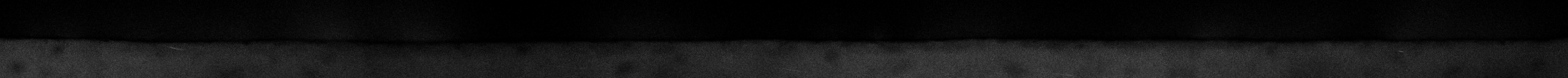

Supplement: Supplementary file 7 — Source data Fig. 6 [file 44321_2025_319_MOESM7_ESM.zip › Figure 6/Panel B/Permeability masks_time1_time2_used for analysis_Serum_free_media_only/PC71_7_Top_SM_SF_slice_8.tif]

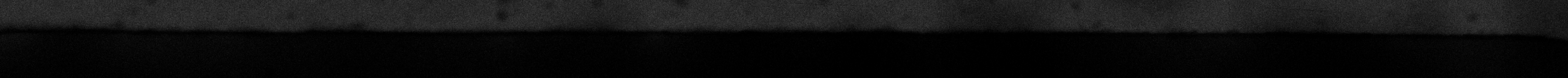

Supplement: Supplementary file 7 — Source data Fig. 6 [file 44321_2025_319_MOESM7_ESM.zip › Figure 6/Panel B/Permeability masks_time1_time2_used for analysis_Serum_free_media_only/PC71_8_Bottom_SM_SF_slice_12.tif]

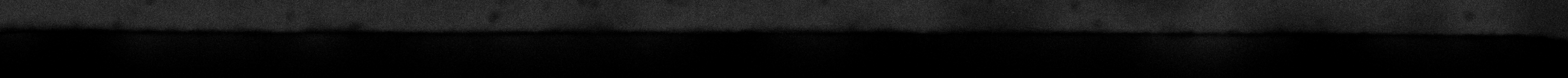

Supplement: Supplementary file 7 — Source data Fig. 6 [file 44321_2025_319_MOESM7_ESM.zip › Figure 6/Panel B/Permeability masks_time1_time2_used for analysis_Serum_free_media_only/PC71_8_Bottom_SM_SF_slice_2.tif]

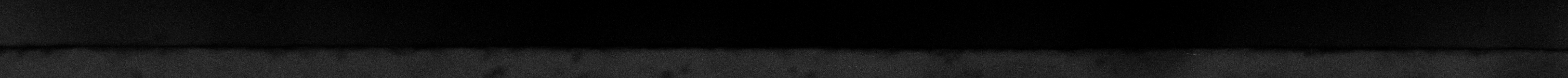

Supplement: Supplementary file 7 — Source data Fig. 6 [file 44321_2025_319_MOESM7_ESM.zip › Figure 6/Panel B/Permeability masks_time1_time2_used for analysis_Serum_free_media_only/PC71_8_Top_SM_SF_slice_12.tif]

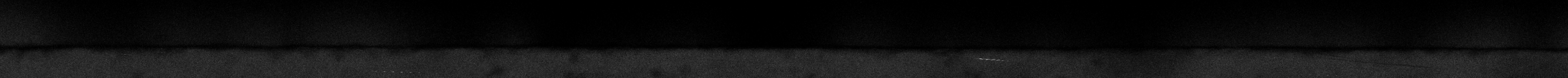

Supplement: Supplementary file 7 — Source data Fig. 6 [file 44321_2025_319_MOESM7_ESM.zip › Figure 6/Panel B/Permeability masks_time1_time2_used for analysis_Serum_free_media_only/PC71_8_Top_SM_SF_slice_2.tif]

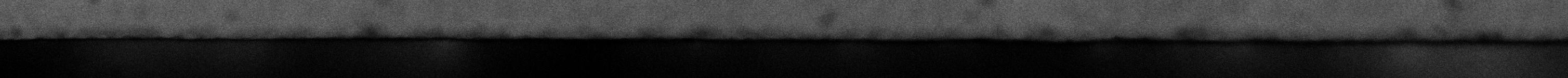

Supplement: Supplementary file 7 — Source data Fig. 6 [file 44321_2025_319_MOESM7_ESM.zip › Figure 6/Panel E/Permeability masks_time1_time2_used for analysis_AKB/PC78_10_Bottom_SM_AKB_slice_16.tif]

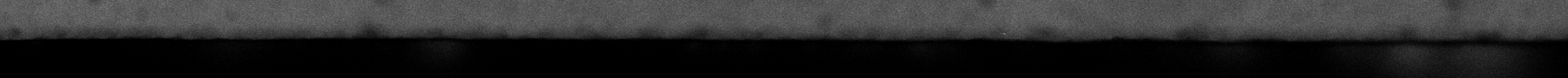

Supplement: Supplementary file 7 — Source data Fig. 6 [file 44321_2025_319_MOESM7_ESM.zip › Figure 6/Panel E/Permeability masks_time1_time2_used for analysis_AKB/PC78_10_Bottom_SM_AKB_slice_6.tif]

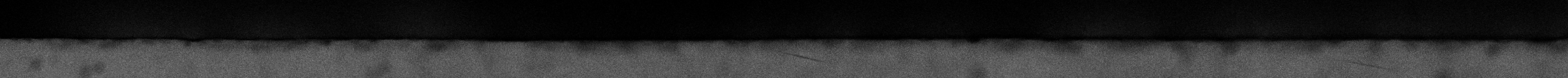

Supplement: Supplementary file 7 — Source data Fig. 6 [file 44321_2025_319_MOESM7_ESM.zip › Figure 6/Panel E/Permeability masks_time1_time2_used for analysis_AKB/PC78_10_Top_SM_AKB_slice_16.tif]

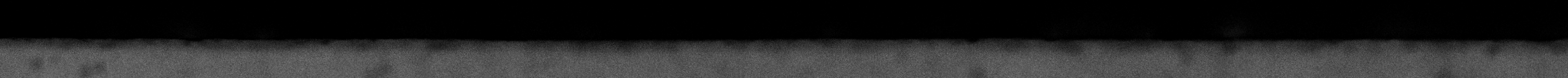

Supplement: Supplementary file 7 — Source data Fig. 6 [file 44321_2025_319_MOESM7_ESM.zip › Figure 6/Panel E/Permeability masks_time1_time2_used for analysis_AKB/PC78_10_Top_SM_AKB_slice_6.tif]

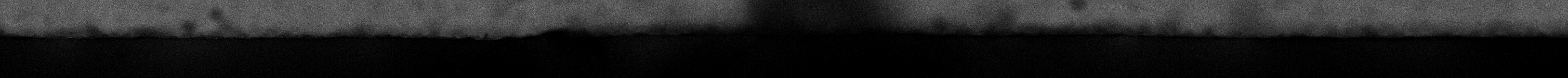

Supplement: Supplementary file 7 — Source data Fig. 6 [file 44321_2025_319_MOESM7_ESM.zip › Figure 6/Panel E/Permeability masks_time1_time2_used for analysis_AKB/PC78_12_Bottom_SM_AKB_slice_15.tif]

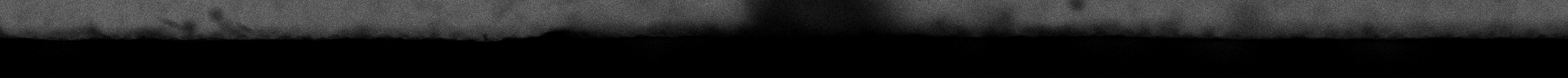

Supplement: Supplementary file 7 — Source data Fig. 6 [file 44321_2025_319_MOESM7_ESM.zip › Figure 6/Panel E/Permeability masks_time1_time2_used for analysis_AKB/PC78_12_Bottom_SM_AKB_slice_5.tif]

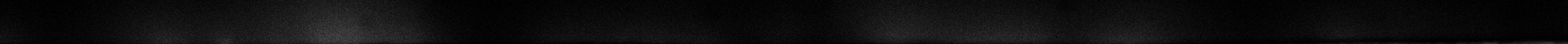

Supplement: Supplementary file 7 — Source data Fig. 6 [file 44321_2025_319_MOESM7_ESM.zip › Figure 6/Panel E/Permeability masks_time1_time2_used for analysis_AKB/PC78_12_Top_SM_AKB_slice_15.tif]

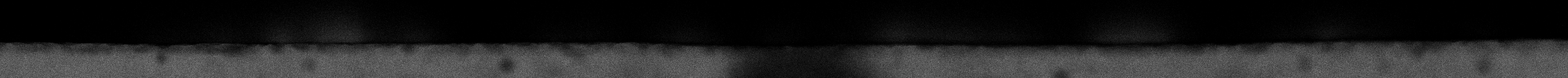

Supplement: Supplementary file 7 — Source data Fig. 6 [file 44321_2025_319_MOESM7_ESM.zip › Figure 6/Panel E/Permeability masks_time1_time2_used for analysis_AKB/PC78_12_Top_SM_AKB_slice_5.tif]

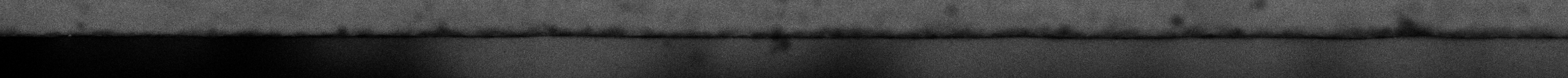

Supplement: Supplementary file 7 — Source data Fig. 6 [file 44321_2025_319_MOESM7_ESM.zip › Figure 6/Panel E/Permeability masks_time1_time2_used for analysis_AKB/PC78_5_Bottom_SM_AKB_slice_15.tif]

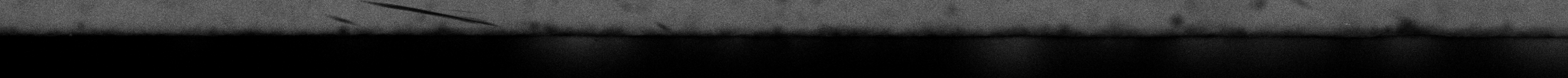

Supplement: Supplementary file 7 — Source data Fig. 6 [file 44321_2025_319_MOESM7_ESM.zip › Figure 6/Panel E/Permeability masks_time1_time2_used for analysis_AKB/PC78_5_Bottom_SM_AKB_slice_5.tif]

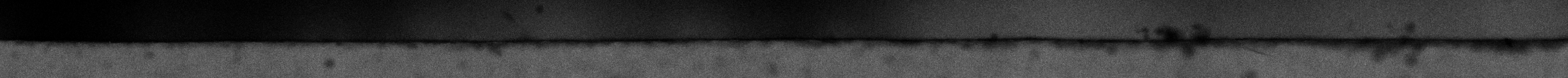

Supplement: Supplementary file 7 — Source data Fig. 6 [file 44321_2025_319_MOESM7_ESM.zip › Figure 6/Panel E/Permeability masks_time1_time2_used for analysis_AKB/PC78_5_Top_SM_AKB_slice_15.tif]

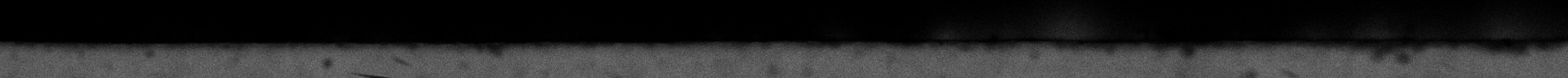

Supplement: Supplementary file 7 — Source data Fig. 6 [file 44321_2025_319_MOESM7_ESM.zip › Figure 6/Panel E/Permeability masks_time1_time2_used for analysis_AKB/PC78_5_Top_SM_AKB_slice_5.tif]

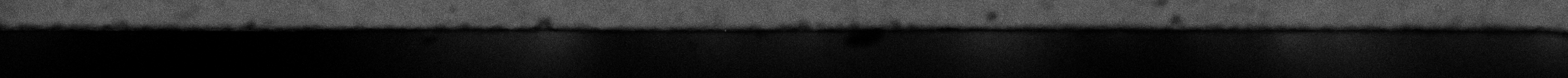

Supplement: Supplementary file 7 — Source data Fig. 6 [file 44321_2025_319_MOESM7_ESM.zip › Figure 6/Panel E/Permeability masks_time1_time2_used for analysis_AKB/PC78_6_Bottom_SM_AKB_slice_15.tif]

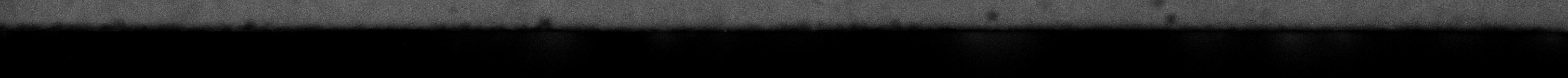

Supplement: Supplementary file 7 — Source data Fig. 6 [file 44321_2025_319_MOESM7_ESM.zip › Figure 6/Panel E/Permeability masks_time1_time2_used for analysis_AKB/PC78_6_Bottom_SM_AKB_slice_5.tif]

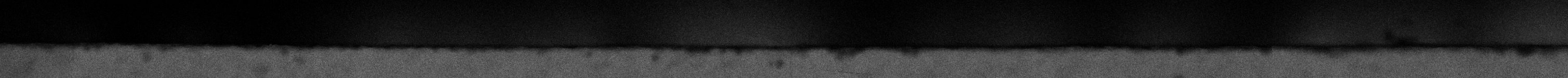

Supplement: Supplementary file 7 — Source data Fig. 6 [file 44321_2025_319_MOESM7_ESM.zip › Figure 6/Panel E/Permeability masks_time1_time2_used for analysis_AKB/PC78_6_Top_SM_AKB_slice_15.tif]

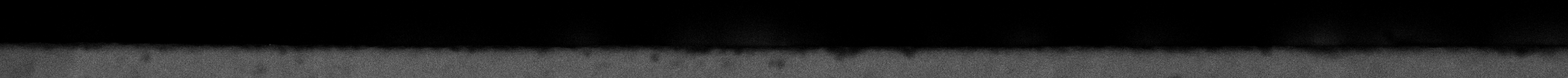

Supplement: Supplementary file 7 — Source data Fig. 6 [file 44321_2025_319_MOESM7_ESM.zip › Figure 6/Panel E/Permeability masks_time1_time2_used for analysis_AKB/PC78_6_Top_SM_AKB_slice_5.tif]

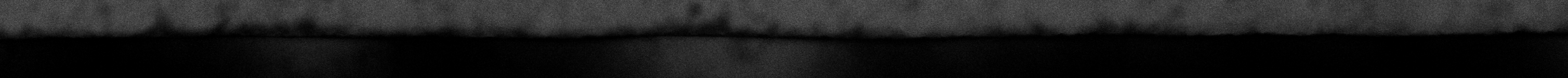

Supplement: Supplementary file 7 — Source data Fig. 6 [file 44321_2025_319_MOESM7_ESM.zip › Figure 6/Panel E/Permeability masks_time1_time2_used for analysis_AKB/PC79_1_Bottom_SM_AKB_slice_14.tif]

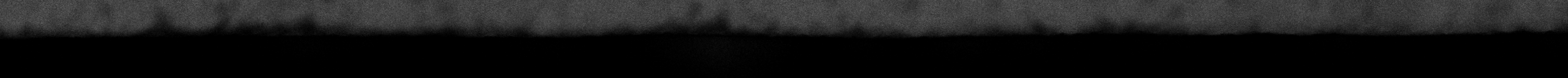

Supplement: Supplementary file 7 — Source data Fig. 6 [file 44321_2025_319_MOESM7_ESM.zip › Figure 6/Panel E/Permeability masks_time1_time2_used for analysis_AKB/PC79_1_Bottom_SM_AKB_slice_4.tif]

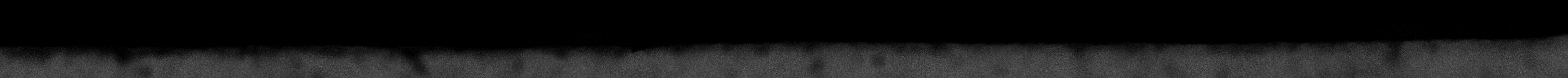

Supplement: Supplementary file 7 — Source data Fig. 6 [file 44321_2025_319_MOESM7_ESM.zip › Figure 6/Panel E/Permeability masks_time1_time2_used for analysis_AKB/PC79_1_Top_SM_AKB_slice_14.tif]

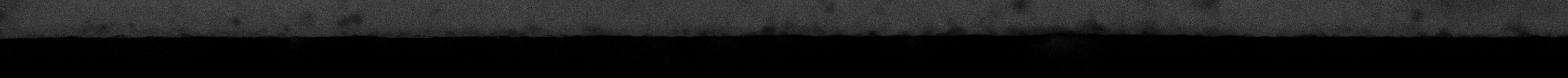

Supplement: Supplementary file 7 — Source data Fig. 6 [file 44321_2025_319_MOESM7_ESM.zip › Figure 6/Panel E/Permeability masks_time1_time2_used for analysis_AKB/PC79_13_Bottom_SM_AKB_slice_16.tif]

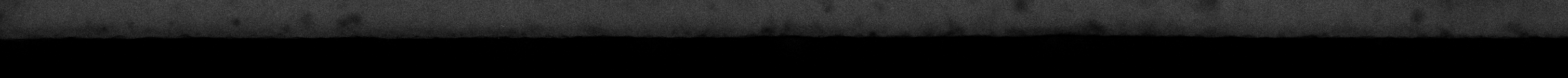

Supplement: Supplementary file 7 — Source data Fig. 6 [file 44321_2025_319_MOESM7_ESM.zip › Figure 6/Panel E/Permeability masks_time1_time2_used for analysis_AKB/PC79_13_Bottom_SM_AKB_slice_6.tif]

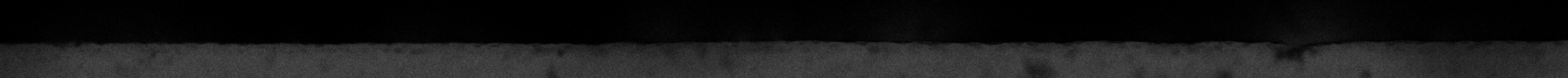

Supplement: Supplementary file 7 — Source data Fig. 6 [file 44321_2025_319_MOESM7_ESM.zip › Figure 6/Panel E/Permeability masks_time1_time2_used for analysis_AKB/PC79_13_Top_SM_AKB_slice_16.tif]

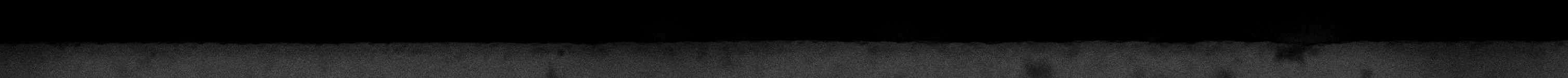

Supplement: Supplementary file 7 — Source data Fig. 6 [file 44321_2025_319_MOESM7_ESM.zip › Figure 6/Panel E/Permeability masks_time1_time2_used for analysis_AKB/PC79_13_Top_SM_AKB_slice_6.tif]

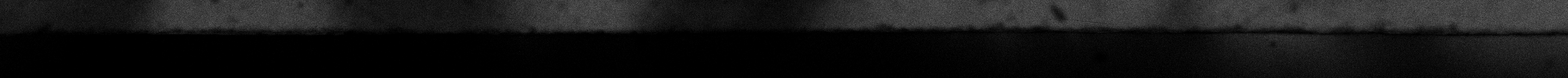

Supplement: Supplementary file 7 — Source data Fig. 6 [file 44321_2025_319_MOESM7_ESM.zip › Figure 6/Panel E/Permeability masks_time1_time2_used for analysis_AKB/PC79_2_Bottom_SM_AKB_slice_15.tif]

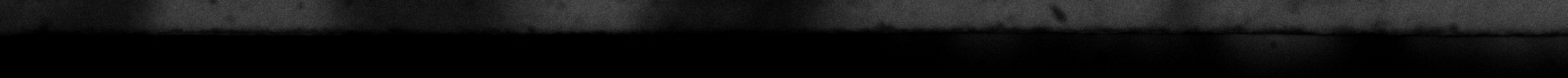

Supplement: Supplementary file 7 — Source data Fig. 6 [file 44321_2025_319_MOESM7_ESM.zip › Figure 6/Panel E/Permeability masks_time1_time2_used for analysis_AKB/PC79_2_Bottom_SM_AKB_slice_5.tif]

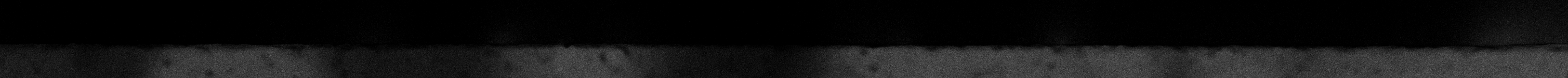

Supplement: Supplementary file 7 — Source data Fig. 6 [file 44321_2025_319_MOESM7_ESM.zip › Figure 6/Panel E/Permeability masks_time1_time2_used for analysis_AKB/PC79_2_Top_SM_AKB_slice_15.tif]

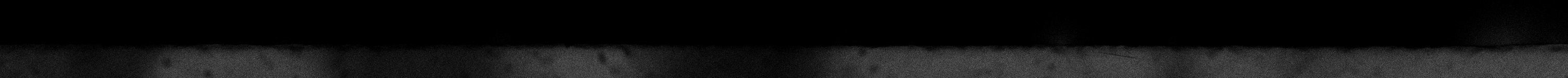

Supplement: Supplementary file 7 — Source data Fig. 6 [file 44321_2025_319_MOESM7_ESM.zip › Figure 6/Panel E/Permeability masks_time1_time2_used for analysis_AKB/PC79_2_Top_SM_AKB_slice_5.tif]

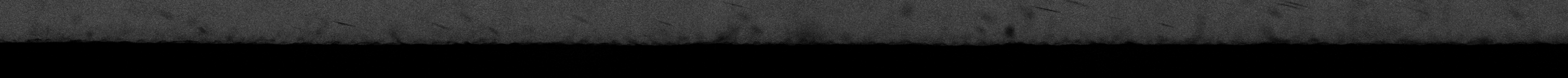

Supplement: Supplementary file 7 — Source data Fig. 6 [file 44321_2025_319_MOESM7_ESM.zip › Figure 6/Panel E/Permeability masks_time1_time2_used for analysis_AKB/PC79_9_Bottom_SM_AKB_slice_14.tif]

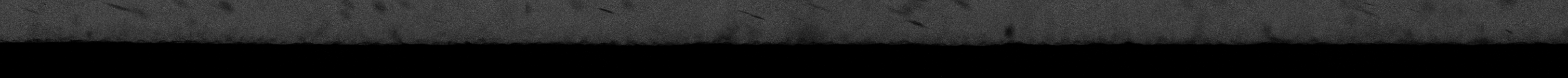

Supplement: Supplementary file 7 — Source data Fig. 6 [file 44321_2025_319_MOESM7_ESM.zip › Figure 6/Panel E/Permeability masks_time1_time2_used for analysis_AKB/PC79_9_Bottom_SM_AKB_slice_4.tif]

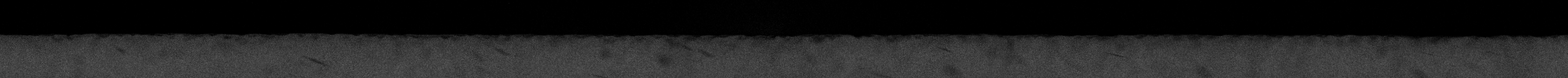

Supplement: Supplementary file 7 — Source data Fig. 6 [file 44321_2025_319_MOESM7_ESM.zip › Figure 6/Panel E/Permeability masks_time1_time2_used for analysis_AKB/PC79_9_Top_SM_AKB_slice_14.tif]

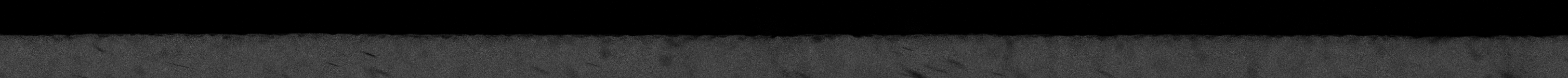

Supplement: Supplementary file 7 — Source data Fig. 6 [file 44321_2025_319_MOESM7_ESM.zip › Figure 6/Panel E/Permeability masks_time1_time2_used for analysis_AKB/PC79_9_Top_SM_AKB_slice_4.tif]

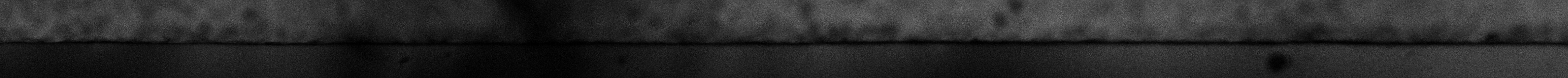

Supplement: Supplementary file 7 — Source data Fig. 6 [file 44321_2025_319_MOESM7_ESM.zip › Figure 6/Panel E/Permeability masks_time1_time2_used for analysis_AKB_RM/PC78_3_Bottom_RM_AKB_slice_14.tif]

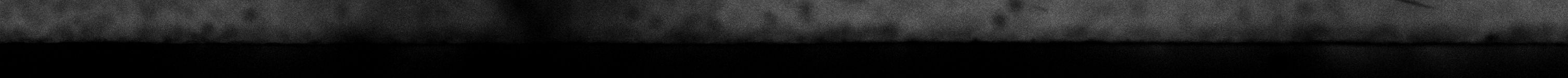

Supplement: Supplementary file 7 — Source data Fig. 6 [file 44321_2025_319_MOESM7_ESM.zip › Figure 6/Panel E/Permeability masks_time1_time2_used for analysis_AKB_RM/PC78_3_Bottom_RM_AKB_slice_4.tif]

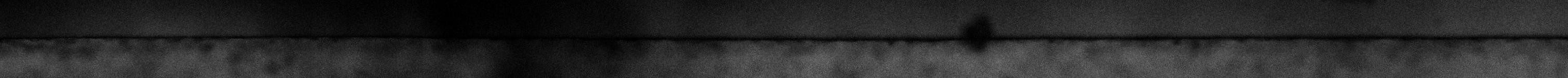

Supplement: Supplementary file 7 — Source data Fig. 6 [file 44321_2025_319_MOESM7_ESM.zip › Figure 6/Panel E/Permeability masks_time1_time2_used for analysis_AKB_RM/PC78_3_Top_RM_AKB_slice_14.tif]

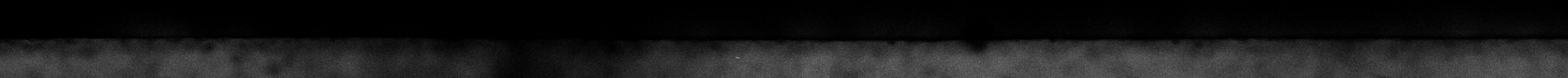

Supplement: Supplementary file 7 — Source data Fig. 6 [file 44321_2025_319_MOESM7_ESM.zip › Figure 6/Panel E/Permeability masks_time1_time2_used for analysis_AKB_RM/PC78_3_Top_RM_AKB_slice_4.tif]

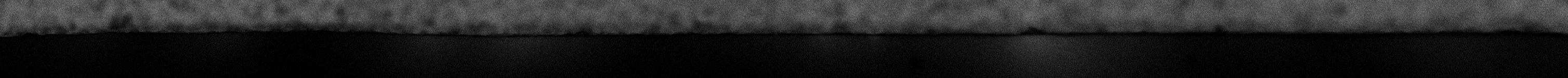

Supplement: Supplementary file 7 — Source data Fig. 6 [file 44321_2025_319_MOESM7_ESM.zip › Figure 6/Panel E/Permeability masks_time1_time2_used for analysis_AKB_RM/PC78_4_Bottom_RM_AKB_slice_14.tif]

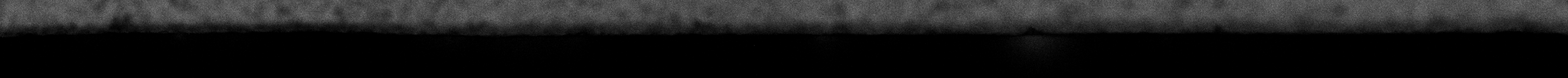

Supplement: Supplementary file 7 — Source data Fig. 6 [file 44321_2025_319_MOESM7_ESM.zip › Figure 6/Panel E/Permeability masks_time1_time2_used for analysis_AKB_RM/PC78_4_Bottom_RM_AKB_slice_4.tif]

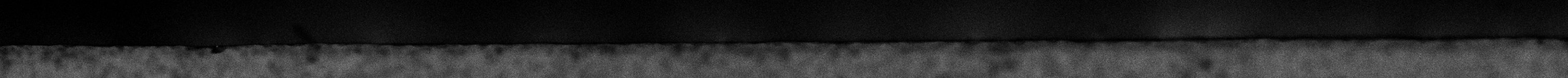

Supplement: Supplementary file 7 — Source data Fig. 6 [file 44321_2025_319_MOESM7_ESM.zip › Figure 6/Panel E/Permeability masks_time1_time2_used for analysis_AKB_RM/PC78_4_Top_RM_AKB_slice_14.tif]
