# Supplementary figures and images for: Plasmodium falciparum impairs Ang-1 secretion by pericytes in a 3D brain microvessel model (part 3 of 4)
Source: EMBO Mol Med. 2025 Oct 16;17(11):3110–38. doi: 10.1038/s44321-025-00319-y (PMC12603187; doi:10.1038/s44321-025-00319-y)

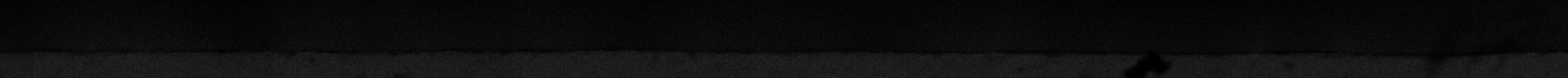

Supplement: Supplementary file 7 — Source data Fig. 6 [file 44321_2025_319_MOESM7_ESM.zip › Figure 6/Panel B/Permeability masks_time1_time2_used for analysis_rAng1/PC66_10_Top_SM_Ang1_slice_17.tif]

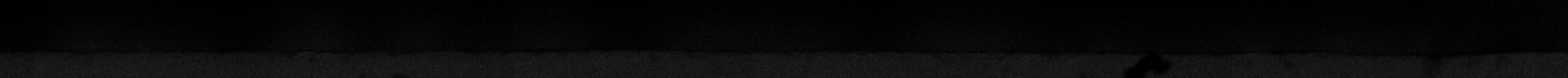

Supplement: Supplementary file 7 — Source data Fig. 6 [file 44321_2025_319_MOESM7_ESM.zip › Figure 6/Panel B/Permeability masks_time1_time2_used for analysis_rAng1/PC66_10_Top_SM_Ang1_slice_7.tif]

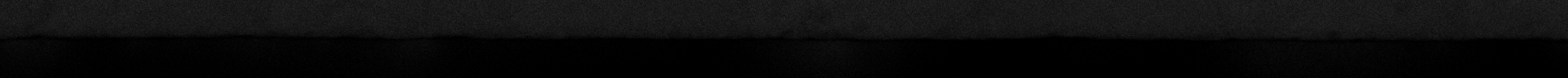

Supplement: Supplementary file 7 — Source data Fig. 6 [file 44321_2025_319_MOESM7_ESM.zip › Figure 6/Panel B/Permeability masks_time1_time2_used for analysis_rAng1/PC66_11_Bottom_SM_Ang1_slice_13.tif]

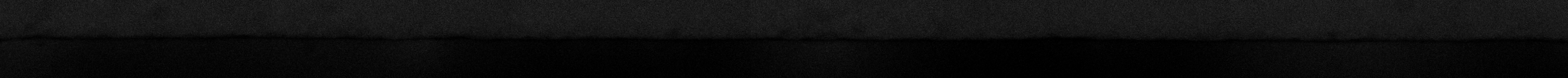

Supplement: Supplementary file 7 — Source data Fig. 6 [file 44321_2025_319_MOESM7_ESM.zip › Figure 6/Panel B/Permeability masks_time1_time2_used for analysis_rAng1/PC66_11_Bottom_SM_Ang1_slice_23.tif]

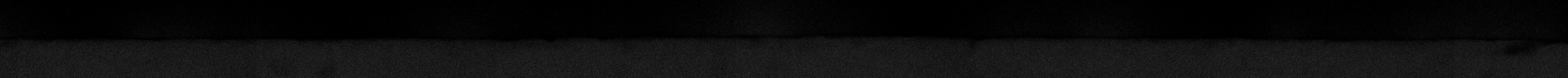

Supplement: Supplementary file 7 — Source data Fig. 6 [file 44321_2025_319_MOESM7_ESM.zip › Figure 6/Panel B/Permeability masks_time1_time2_used for analysis_rAng1/PC66_11_Top_SM_Ang1_slice_13.tif]

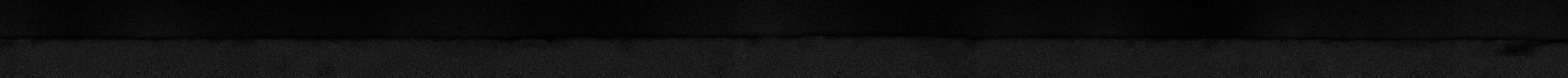

Supplement: Supplementary file 7 — Source data Fig. 6 [file 44321_2025_319_MOESM7_ESM.zip › Figure 6/Panel B/Permeability masks_time1_time2_used for analysis_rAng1/PC66_11_Top_SM_Ang1_slice_23.tif]

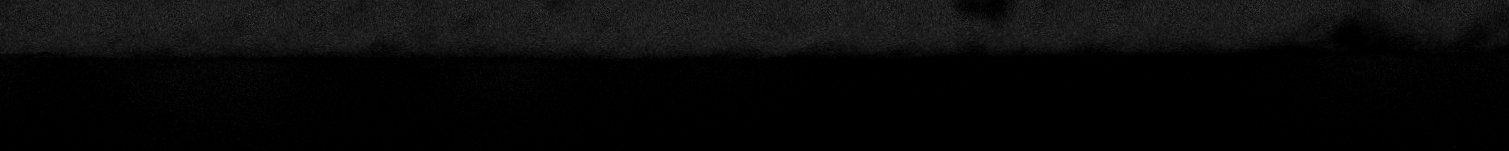

Supplement: Supplementary file 7 — Source data Fig. 6 [file 44321_2025_319_MOESM7_ESM.zip › Figure 6/Panel B/Permeability masks_time1_time2_used for analysis_rAng1/PC66_4_Bottom_SM_Ang1_slice_13.tif]

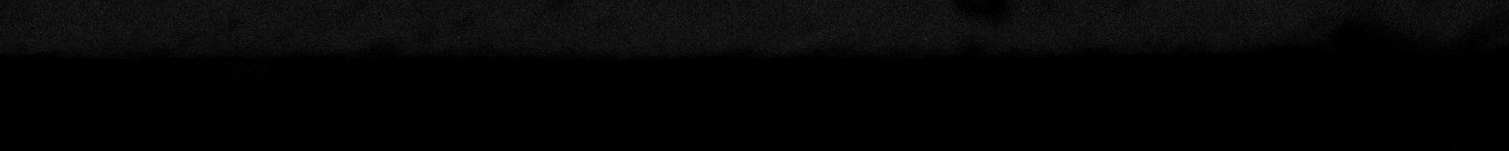

Supplement: Supplementary file 7 — Source data Fig. 6 [file 44321_2025_319_MOESM7_ESM.zip › Figure 6/Panel B/Permeability masks_time1_time2_used for analysis_rAng1/PC66_4_Bottom_SM_Ang1_slice_6.tif]

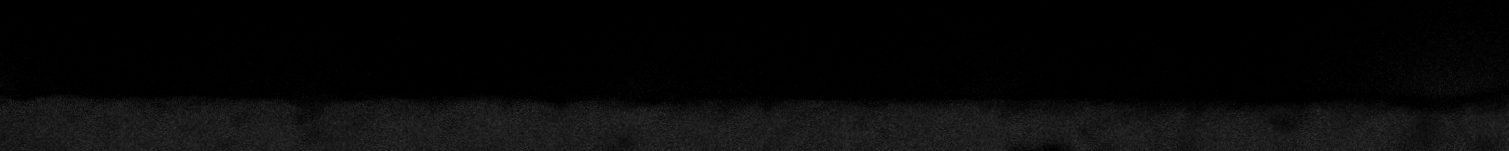

Supplement: Supplementary file 7 — Source data Fig. 6 [file 44321_2025_319_MOESM7_ESM.zip › Figure 6/Panel B/Permeability masks_time1_time2_used for analysis_rAng1/PC66_4_Top_SM_Ang1_slice_13.tif]

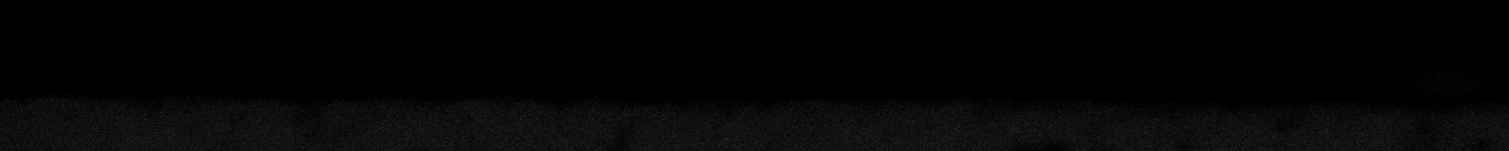

Supplement: Supplementary file 7 — Source data Fig. 6 [file 44321_2025_319_MOESM7_ESM.zip › Figure 6/Panel B/Permeability masks_time1_time2_used for analysis_rAng1/PC66_4_Top_SM_Ang1_slice_6.tif]

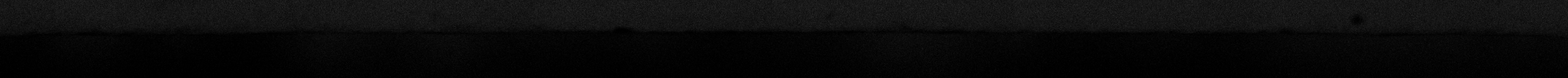

Supplement: Supplementary file 7 — Source data Fig. 6 [file 44321_2025_319_MOESM7_ESM.zip › Figure 6/Panel B/Permeability masks_time1_time2_used for analysis_rAng1/PC66_9_Bottom_SM_Ang1_slice_10.tif]

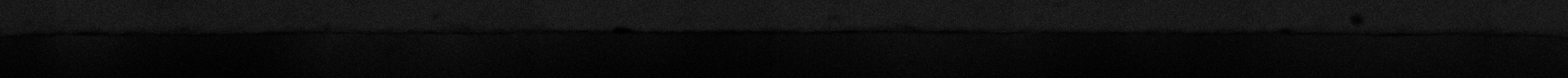

Supplement: Supplementary file 7 — Source data Fig. 6 [file 44321_2025_319_MOESM7_ESM.zip › Figure 6/Panel B/Permeability masks_time1_time2_used for analysis_rAng1/PC66_9_Bottom_SM_Ang1_slice_20.tif]

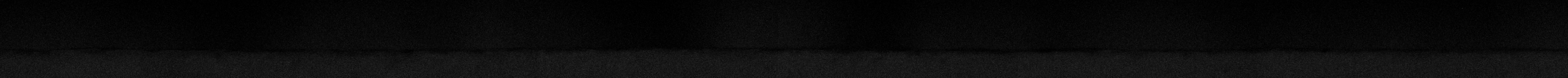

Supplement: Supplementary file 7 — Source data Fig. 6 [file 44321_2025_319_MOESM7_ESM.zip › Figure 6/Panel B/Permeability masks_time1_time2_used for analysis_rAng1/PC66_9_Top_SM_Ang1_slice_10.tif]

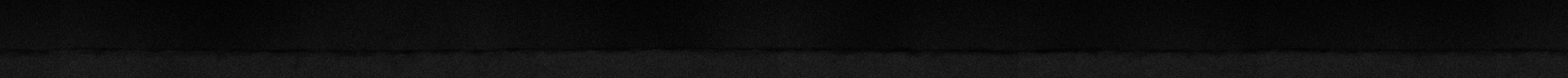

Supplement: Supplementary file 7 — Source data Fig. 6 [file 44321_2025_319_MOESM7_ESM.zip › Figure 6/Panel B/Permeability masks_time1_time2_used for analysis_rAng1/PC66_9_Top_SM_Ang1_slice_20.tif]

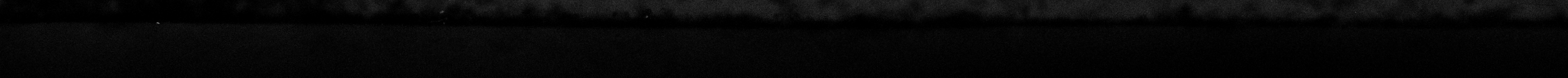

Supplement: Supplementary file 7 — Source data Fig. 6 [file 44321_2025_319_MOESM7_ESM.zip › Figure 6/Panel B/Permeability masks_time1_time2_used for analysis_RM/PC88_4_Bottom_RM_slice12.tif]

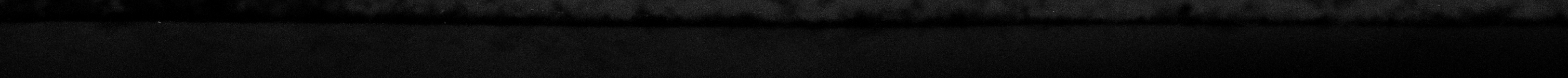

Supplement: Supplementary file 7 — Source data Fig. 6 [file 44321_2025_319_MOESM7_ESM.zip › Figure 6/Panel B/Permeability masks_time1_time2_used for analysis_RM/PC88_4_Bottom_RM_slice22.tif]

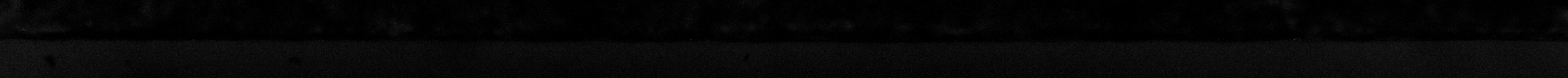

Supplement: Supplementary file 7 — Source data Fig. 6 [file 44321_2025_319_MOESM7_ESM.zip › Figure 6/Panel B/Permeability masks_time1_time2_used for analysis_RM/PC88_9_Bottom_RM_slice16.tif]

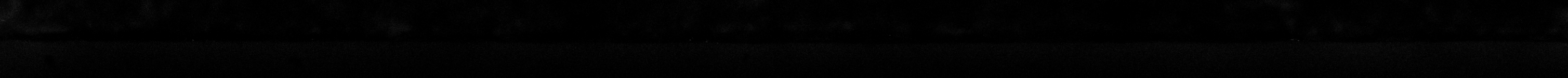

Supplement: Supplementary file 7 — Source data Fig. 6 [file 44321_2025_319_MOESM7_ESM.zip › Figure 6/Panel B/Permeability masks_time1_time2_used for analysis_RM/PC88_9_Bottom_RM_slice6.tif]

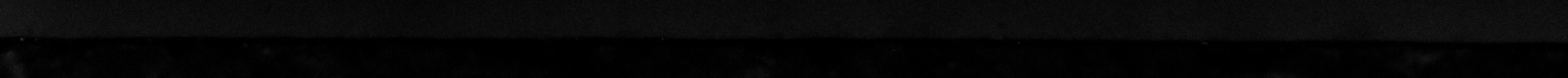

Supplement: Supplementary file 7 — Source data Fig. 6 [file 44321_2025_319_MOESM7_ESM.zip › Figure 6/Panel B/Permeability masks_time1_time2_used for analysis_RM/PC88_9_Top_RM_slice19.tif]

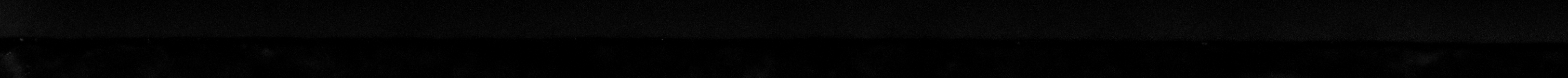

Supplement: Supplementary file 7 — Source data Fig. 6 [file 44321_2025_319_MOESM7_ESM.zip › Figure 6/Panel B/Permeability masks_time1_time2_used for analysis_RM/PC88_9_Top_RM_slice9.tif]

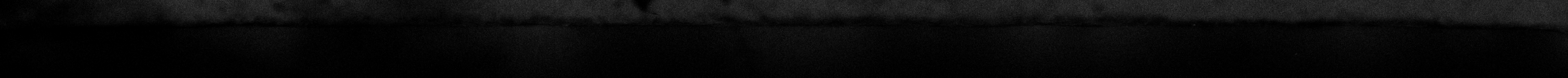

Supplement: Supplementary file 7 — Source data Fig. 6 [file 44321_2025_319_MOESM7_ESM.zip › Figure 6/Panel B/Permeability masks_time1_time2_used for analysis_RM/PC90_11_Bottom_RM_slice10.tif]

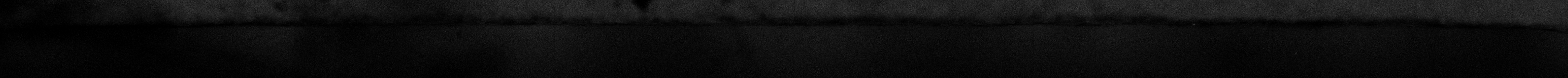

Supplement: Supplementary file 7 — Source data Fig. 6 [file 44321_2025_319_MOESM7_ESM.zip › Figure 6/Panel B/Permeability masks_time1_time2_used for analysis_RM/PC90_11_Bottom_RM_slice20.tif]

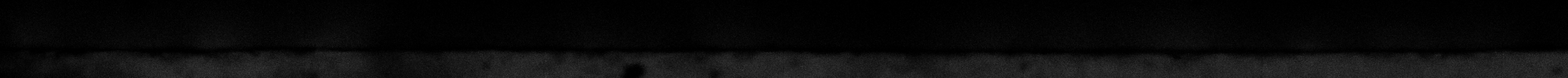

Supplement: Supplementary file 7 — Source data Fig. 6 [file 44321_2025_319_MOESM7_ESM.zip › Figure 6/Panel B/Permeability masks_time1_time2_used for analysis_RM/PC90_11_Top_RM_slice10.tif]

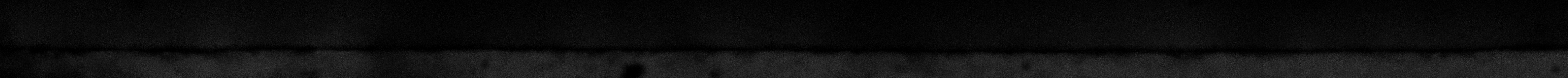

Supplement: Supplementary file 7 — Source data Fig. 6 [file 44321_2025_319_MOESM7_ESM.zip › Figure 6/Panel B/Permeability masks_time1_time2_used for analysis_RM/PC90_11_Top_RM_slice20.tif]

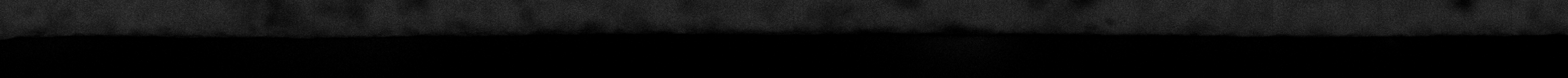

Supplement: Supplementary file 7 — Source data Fig. 6 [file 44321_2025_319_MOESM7_ESM.zip › Figure 6/Panel B/Permeability masks_time1_time2_used for analysis_RM/PC90_12_Bottom_RM_slice10.tif]

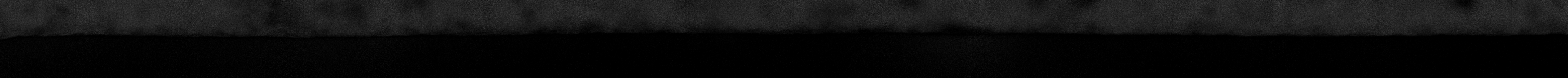

Supplement: Supplementary file 7 — Source data Fig. 6 [file 44321_2025_319_MOESM7_ESM.zip › Figure 6/Panel B/Permeability masks_time1_time2_used for analysis_RM/PC90_12_Bottom_RM_slice20.tif]

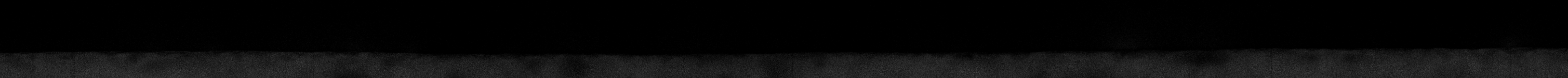

Supplement: Supplementary file 7 — Source data Fig. 6 [file 44321_2025_319_MOESM7_ESM.zip › Figure 6/Panel B/Permeability masks_time1_time2_used for analysis_RM/PC90_12_Top_RM_slice10.tif]

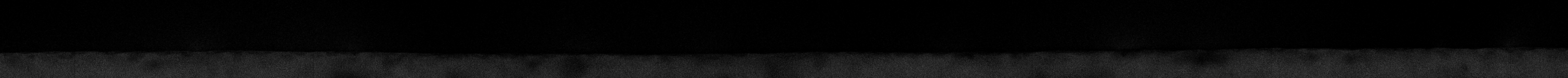

Supplement: Supplementary file 7 — Source data Fig. 6 [file 44321_2025_319_MOESM7_ESM.zip › Figure 6/Panel B/Permeability masks_time1_time2_used for analysis_RM/PC90_12_Top_RM_slice20.tif]

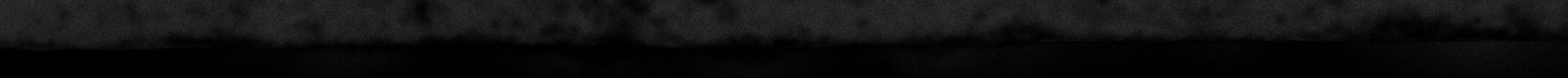

Supplement: Supplementary file 7 — Source data Fig. 6 [file 44321_2025_319_MOESM7_ESM.zip › Figure 6/Panel B/Permeability masks_time1_time2_used for analysis_RM+rAng1/PC65_1_Bottom_RM_Ang1_slice_14.tif]

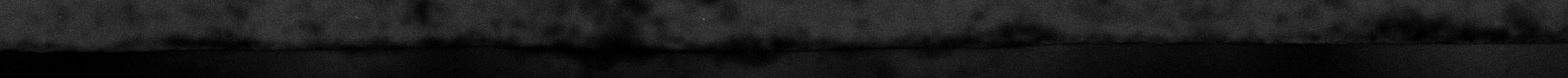

Supplement: Supplementary file 7 — Source data Fig. 6 [file 44321_2025_319_MOESM7_ESM.zip › Figure 6/Panel B/Permeability masks_time1_time2_used for analysis_RM+rAng1/PC65_1_Bottom_RM_Ang1_slice_24.tif]

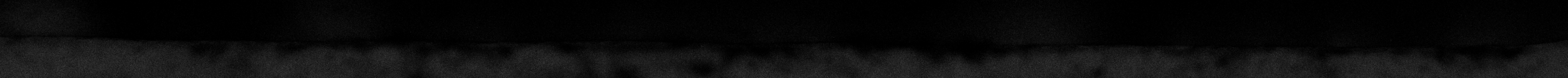

Supplement: Supplementary file 7 — Source data Fig. 6 [file 44321_2025_319_MOESM7_ESM.zip › Figure 6/Panel B/Permeability masks_time1_time2_used for analysis_RM+rAng1/PC65_1_Top_RM_Ang1_slice_14.tif]

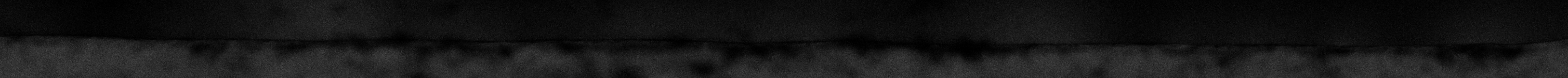

Supplement: Supplementary file 7 — Source data Fig. 6 [file 44321_2025_319_MOESM7_ESM.zip › Figure 6/Panel B/Permeability masks_time1_time2_used for analysis_RM+rAng1/PC65_1_Top_RM_Ang1_slice_24.tif]

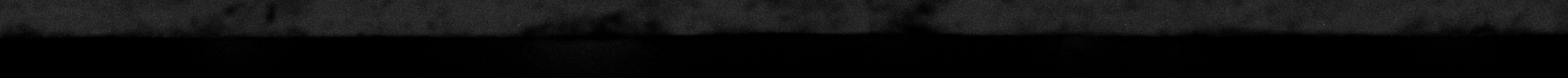

Supplement: Supplementary file 7 — Source data Fig. 6 [file 44321_2025_319_MOESM7_ESM.zip › Figure 6/Panel B/Permeability masks_time1_time2_used for analysis_RM+rAng1/PC65_4_Bottom_RM_Ang1_slice_12.tif]

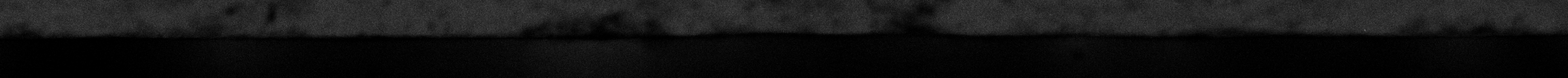

Supplement: Supplementary file 7 — Source data Fig. 6 [file 44321_2025_319_MOESM7_ESM.zip › Figure 6/Panel B/Permeability masks_time1_time2_used for analysis_RM+rAng1/PC65_4_Bottom_RM_Ang1_slice_22.tif]

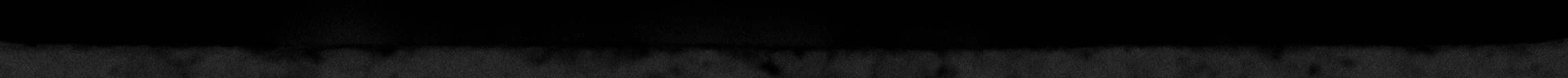

Supplement: Supplementary file 7 — Source data Fig. 6 [file 44321_2025_319_MOESM7_ESM.zip › Figure 6/Panel B/Permeability masks_time1_time2_used for analysis_RM+rAng1/PC65_4_Top_RM_Ang1_slice_12.tif]

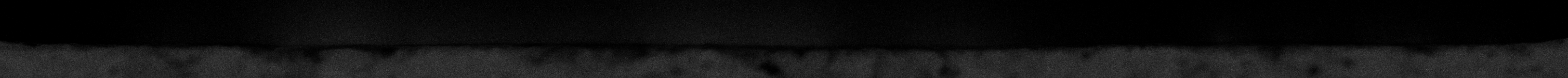

Supplement: Supplementary file 7 — Source data Fig. 6 [file 44321_2025_319_MOESM7_ESM.zip › Figure 6/Panel B/Permeability masks_time1_time2_used for analysis_RM+rAng1/PC65_4_Top_RM_Ang1_slice_22.tif]

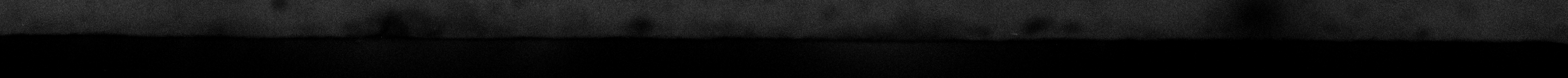

Supplement: Supplementary file 7 — Source data Fig. 6 [file 44321_2025_319_MOESM7_ESM.zip › Figure 6/Panel B/Permeability masks_time1_time2_used for analysis_RM+rAng1/PC65_6_Bottom_RM_Ang1_slice_10.tif]

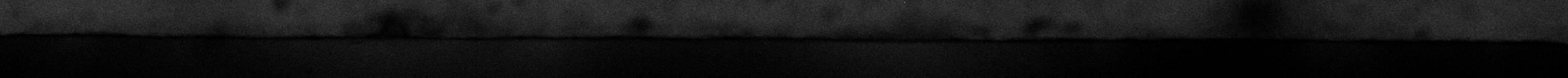

Supplement: Supplementary file 7 — Source data Fig. 6 [file 44321_2025_319_MOESM7_ESM.zip › Figure 6/Panel B/Permeability masks_time1_time2_used for analysis_RM+rAng1/PC65_6_Bottom_RM_Ang1_slice_20.tif]

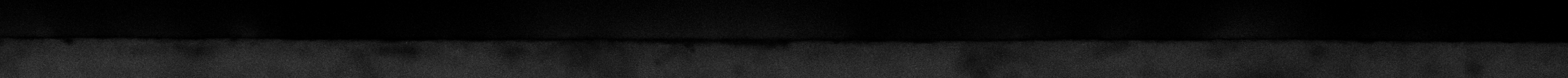

Supplement: Supplementary file 7 — Source data Fig. 6 [file 44321_2025_319_MOESM7_ESM.zip › Figure 6/Panel B/Permeability masks_time1_time2_used for analysis_RM+rAng1/PC65_6_Top_RM_Ang1_slice_10.tif]

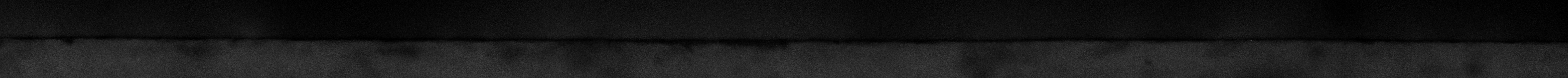

Supplement: Supplementary file 7 — Source data Fig. 6 [file 44321_2025_319_MOESM7_ESM.zip › Figure 6/Panel B/Permeability masks_time1_time2_used for analysis_RM+rAng1/PC65_6_Top_RM_Ang1_slice_20.tif]

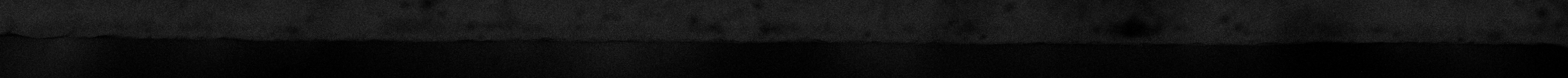

Supplement: Supplementary file 7 — Source data Fig. 6 [file 44321_2025_319_MOESM7_ESM.zip › Figure 6/Panel B/Permeability masks_time1_time2_used for analysis_RM+rAng1/PC65_9_Bottom_RM_Ang1_slice_15.tif]

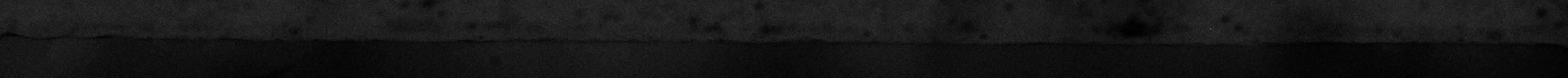

Supplement: Supplementary file 7 — Source data Fig. 6 [file 44321_2025_319_MOESM7_ESM.zip › Figure 6/Panel B/Permeability masks_time1_time2_used for analysis_RM+rAng1/PC65_9_Bottom_RM_Ang1_slice_25.tif]

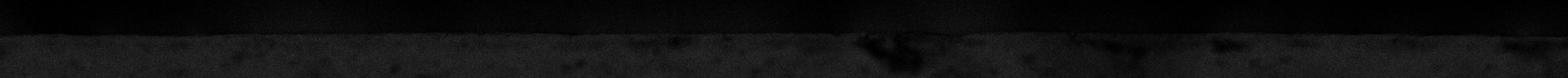

Supplement: Supplementary file 7 — Source data Fig. 6 [file 44321_2025_319_MOESM7_ESM.zip › Figure 6/Panel B/Permeability masks_time1_time2_used for analysis_RM+rAng1/PC65_9_Top_RM_Ang1_slice_15.tif]

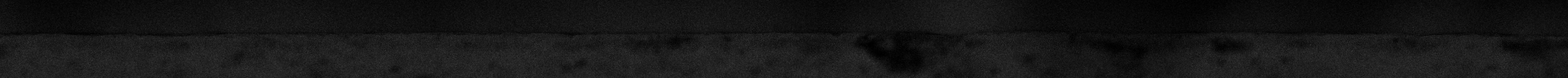

Supplement: Supplementary file 7 — Source data Fig. 6 [file 44321_2025_319_MOESM7_ESM.zip › Figure 6/Panel B/Permeability masks_time1_time2_used for analysis_RM+rAng1/PC65_9_Top_RM_Ang1_slice_25.tif]

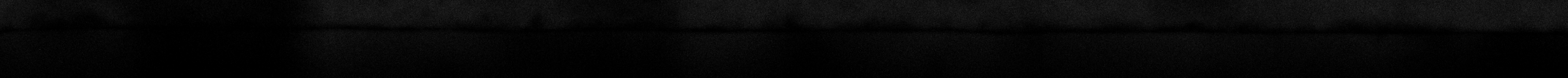

Supplement: Supplementary file 7 — Source data Fig. 6 [file 44321_2025_319_MOESM7_ESM.zip › Figure 6/Panel B/Permeability masks_time1_time2_used for analysis_RM+rAng1/PC66_12_Bottom_RM_Ang1_slice_10.tif]

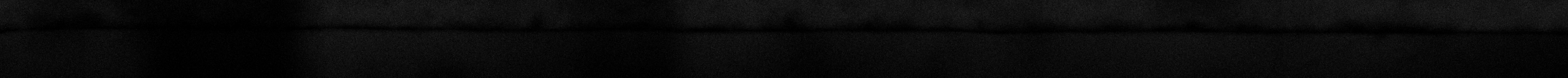

Supplement: Supplementary file 7 — Source data Fig. 6 [file 44321_2025_319_MOESM7_ESM.zip › Figure 6/Panel B/Permeability masks_time1_time2_used for analysis_RM+rAng1/PC66_12_Bottom_RM_Ang1_slice_20.tif]

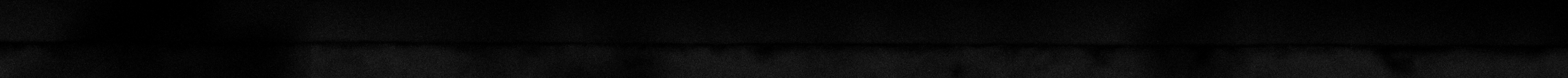

Supplement: Supplementary file 7 — Source data Fig. 6 [file 44321_2025_319_MOESM7_ESM.zip › Figure 6/Panel B/Permeability masks_time1_time2_used for analysis_RM+rAng1/PC66_12_Top_RM_Ang1_slice_10.tif]

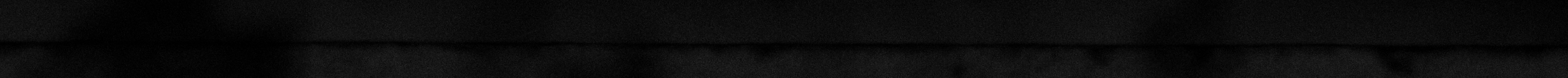

Supplement: Supplementary file 7 — Source data Fig. 6 [file 44321_2025_319_MOESM7_ESM.zip › Figure 6/Panel B/Permeability masks_time1_time2_used for analysis_RM+rAng1/PC66_12_Top_RM_Ang1_slice_20.tif]

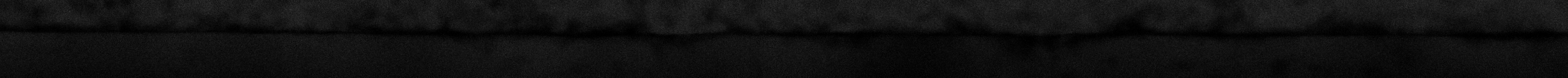

Supplement: Supplementary file 7 — Source data Fig. 6 [file 44321_2025_319_MOESM7_ESM.zip › Figure 6/Panel B/Permeability masks_time1_time2_used for analysis_RM+rAng1/PC66_3_Bottom_RM_Ang1_slice_14.tif]

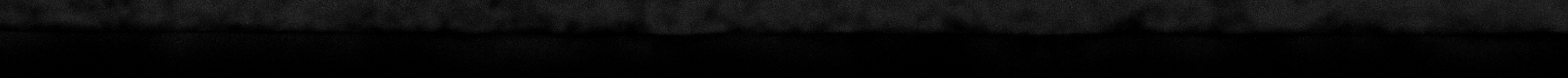

Supplement: Supplementary file 7 — Source data Fig. 6 [file 44321_2025_319_MOESM7_ESM.zip › Figure 6/Panel B/Permeability masks_time1_time2_used for analysis_RM+rAng1/PC66_3_Bottom_RM_Ang1_slice_4.tif]

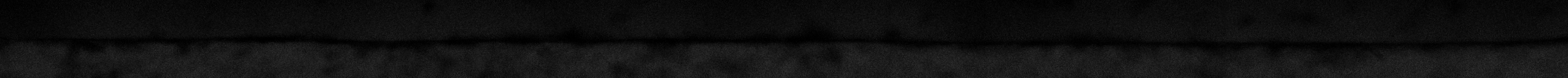

Supplement: Supplementary file 7 — Source data Fig. 6 [file 44321_2025_319_MOESM7_ESM.zip › Figure 6/Panel B/Permeability masks_time1_time2_used for analysis_RM+rAng1/PC66_3_Top_RM_Ang1_slice_14.tif]

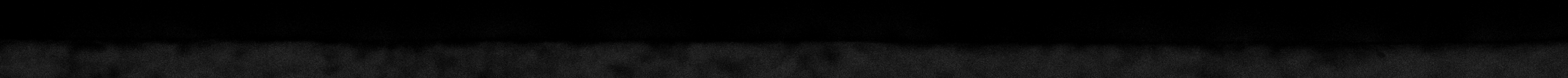

Supplement: Supplementary file 7 — Source data Fig. 6 [file 44321_2025_319_MOESM7_ESM.zip › Figure 6/Panel B/Permeability masks_time1_time2_used for analysis_RM+rAng1/PC66_3_Top_RM_Ang1_slice_4.tif]

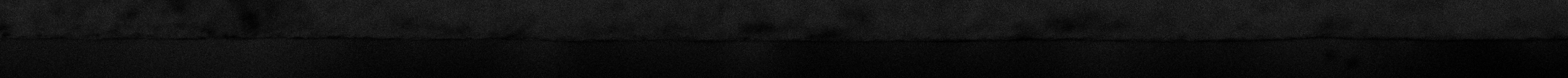

Supplement: Supplementary file 7 — Source data Fig. 6 [file 44321_2025_319_MOESM7_ESM.zip › Figure 6/Panel B/Permeability masks_time1_time2_used for analysis_RM+rAng1/PC66_6_Bottom_RM_Ang1_slice_17.tif]

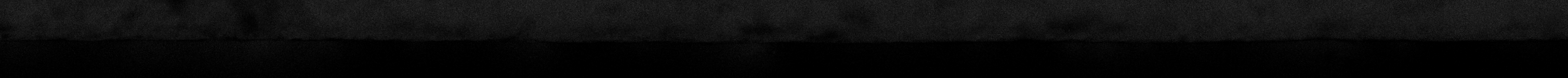

Supplement: Supplementary file 7 — Source data Fig. 6 [file 44321_2025_319_MOESM7_ESM.zip › Figure 6/Panel B/Permeability masks_time1_time2_used for analysis_RM+rAng1/PC66_6_Bottom_RM_Ang1_slice_7.tif]

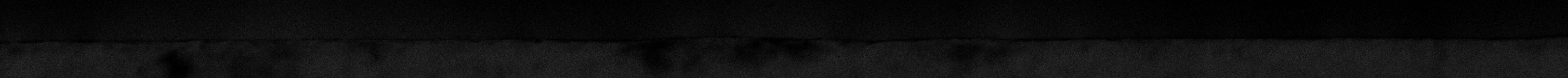

Supplement: Supplementary file 7 — Source data Fig. 6 [file 44321_2025_319_MOESM7_ESM.zip › Figure 6/Panel B/Permeability masks_time1_time2_used for analysis_RM+rAng1/PC66_6_Top_RM_Ang1_slice_17.tif]

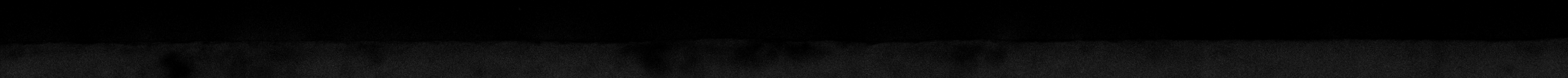

Supplement: Supplementary file 7 — Source data Fig. 6 [file 44321_2025_319_MOESM7_ESM.zip › Figure 6/Panel B/Permeability masks_time1_time2_used for analysis_RM+rAng1/PC66_6_Top_RM_Ang1_slice_7.tif]

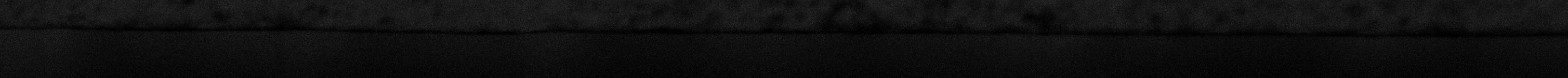

Supplement: Supplementary file 7 — Source data Fig. 6 [file 44321_2025_319_MOESM7_ESM.zip › Figure 6/Panel B/Permeability masks_time1_time2_used for analysis_RM+rAng1/PC66_7_Bottom_RM_Ang1_slice_19.tif]

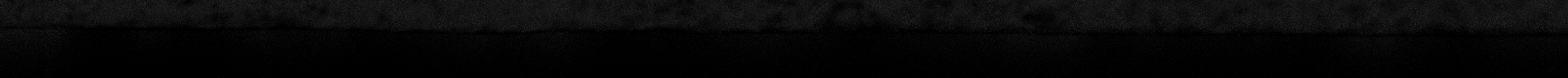

Supplement: Supplementary file 7 — Source data Fig. 6 [file 44321_2025_319_MOESM7_ESM.zip › Figure 6/Panel B/Permeability masks_time1_time2_used for analysis_RM+rAng1/PC66_7_Bottom_RM_Ang1_slice_9.tif]

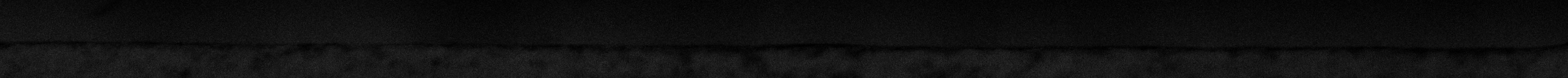

Supplement: Supplementary file 7 — Source data Fig. 6 [file 44321_2025_319_MOESM7_ESM.zip › Figure 6/Panel B/Permeability masks_time1_time2_used for analysis_RM+rAng1/PC66_7_Top_RM_Ang1_slice_19.tif]

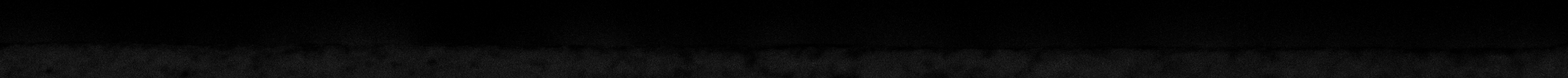

Supplement: Supplementary file 7 — Source data Fig. 6 [file 44321_2025_319_MOESM7_ESM.zip › Figure 6/Panel B/Permeability masks_time1_time2_used for analysis_RM+rAng1/PC66_7_Top_RM_Ang1_slice_9.tif]

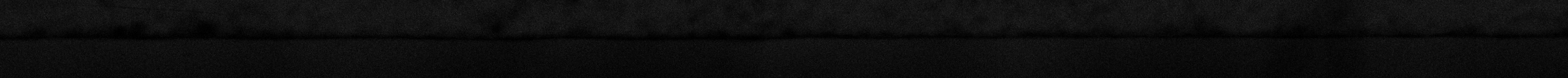

Supplement: Supplementary file 7 — Source data Fig. 6 [file 44321_2025_319_MOESM7_ESM.zip › Figure 6/Panel B/Permeability masks_time1_time2_used for analysis_RM+rAng1/PC66_8_Bottom_RM_Ang1_slice_17.tif]

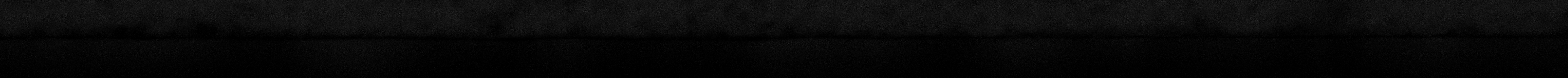

Supplement: Supplementary file 7 — Source data Fig. 6 [file 44321_2025_319_MOESM7_ESM.zip › Figure 6/Panel B/Permeability masks_time1_time2_used for analysis_RM+rAng1/PC66_8_Bottom_RM_Ang1_slice_7.tif]

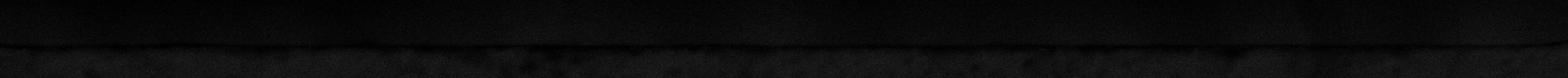

Supplement: Supplementary file 7 — Source data Fig. 6 [file 44321_2025_319_MOESM7_ESM.zip › Figure 6/Panel B/Permeability masks_time1_time2_used for analysis_RM+rAng1/PC66_8_Top_RM_Ang1_slice_17.tif]

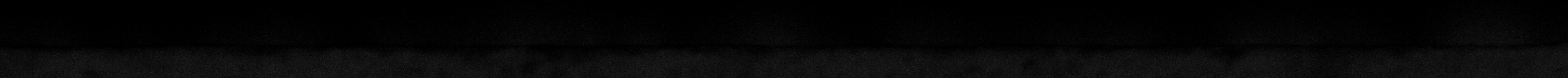

Supplement: Supplementary file 7 — Source data Fig. 6 [file 44321_2025_319_MOESM7_ESM.zip › Figure 6/Panel B/Permeability masks_time1_time2_used for analysis_RM+rAng1/PC66_8_Top_RM_Ang1_slice_7.tif]

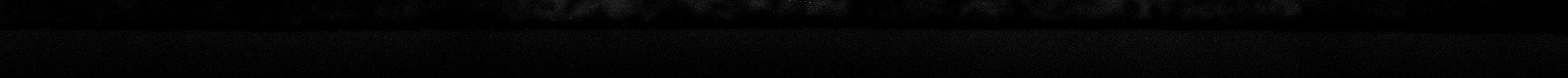

Supplement: Supplementary file 7 — Source data Fig. 6 [file 44321_2025_319_MOESM7_ESM.zip › Figure 6/Panel B/Permeability masks_time1_time2_used for analysis_RM+rAng1/PC88_5_Bottom_RM_rAng1_slice12.tif]

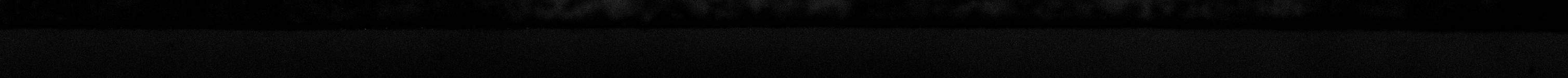

Supplement: Supplementary file 7 — Source data Fig. 6 [file 44321_2025_319_MOESM7_ESM.zip › Figure 6/Panel B/Permeability masks_time1_time2_used for analysis_RM+rAng1/PC88_5_Bottom_RM_rAng1_slice22.tif]

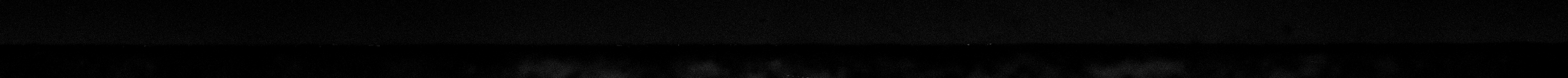

Supplement: Supplementary file 7 — Source data Fig. 6 [file 44321_2025_319_MOESM7_ESM.zip › Figure 6/Panel B/Permeability masks_time1_time2_used for analysis_RM+rAng1/PC88_5_Top_RM_rAng1_slice12.tif]

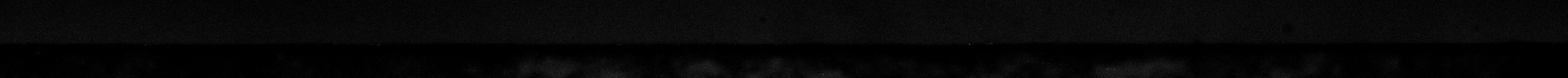

Supplement: Supplementary file 7 — Source data Fig. 6 [file 44321_2025_319_MOESM7_ESM.zip › Figure 6/Panel B/Permeability masks_time1_time2_used for analysis_RM+rAng1/PC88_5_Top_RM_rAng1_slice22.tif]

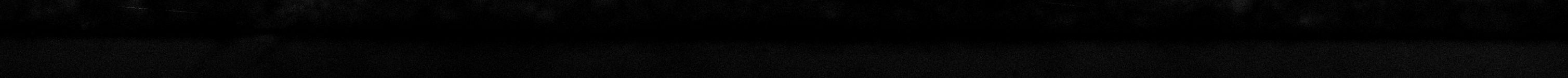

Supplement: Supplementary file 7 — Source data Fig. 6 [file 44321_2025_319_MOESM7_ESM.zip › Figure 6/Panel B/Permeability masks_time1_time2_used for analysis_RM+rAng1/PC88_6_Bottom_RM_rAng1_slice18.tif]

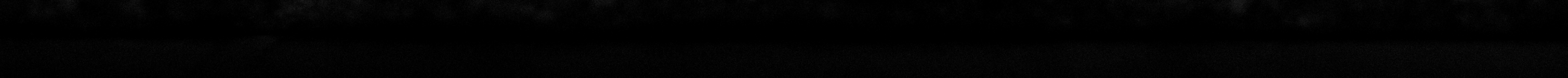

Supplement: Supplementary file 7 — Source data Fig. 6 [file 44321_2025_319_MOESM7_ESM.zip › Figure 6/Panel B/Permeability masks_time1_time2_used for analysis_RM+rAng1/PC88_6_Bottom_RM_rAng1_slice8.tif]

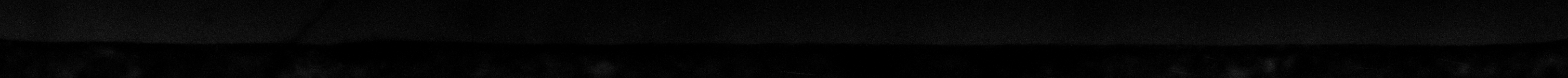

Supplement: Supplementary file 7 — Source data Fig. 6 [file 44321_2025_319_MOESM7_ESM.zip › Figure 6/Panel B/Permeability masks_time1_time2_used for analysis_RM+rAng1/PC88_6_Top_RM_rAng1_slice18.tif]

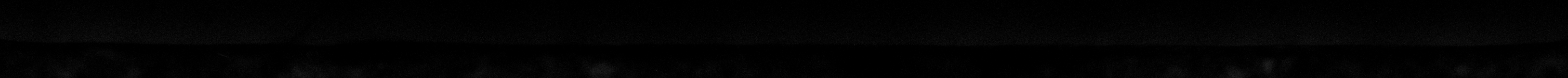

Supplement: Supplementary file 7 — Source data Fig. 6 [file 44321_2025_319_MOESM7_ESM.zip › Figure 6/Panel B/Permeability masks_time1_time2_used for analysis_RM+rAng1/PC88_6_Top_RM_rAng1_slice8.tif]

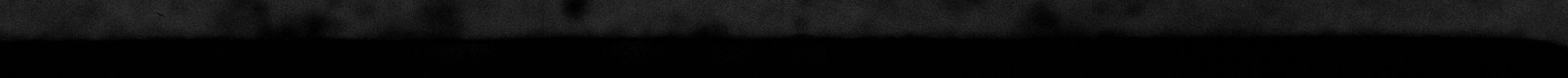

Supplement: Supplementary file 7 — Source data Fig. 6 [file 44321_2025_319_MOESM7_ESM.zip › Figure 6/Panel B/Permeability masks_time1_time2_used for analysis_RM+rAng1/PC90_10_Bottom_RM_Ang1_slice18.tif]

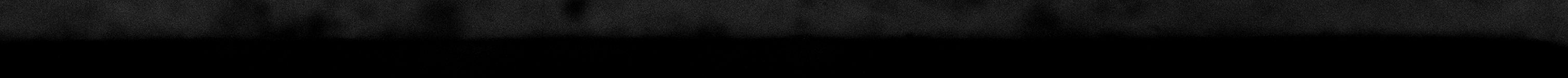

Supplement: Supplementary file 7 — Source data Fig. 6 [file 44321_2025_319_MOESM7_ESM.zip › Figure 6/Panel B/Permeability masks_time1_time2_used for analysis_RM+rAng1/PC90_10_Bottom_RM_Ang1_slice8.tif]

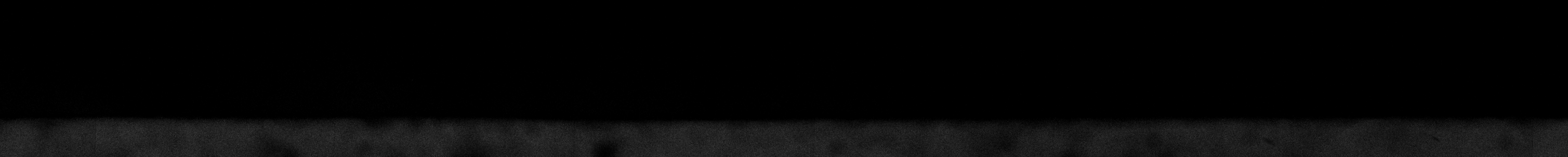

Supplement: Supplementary file 7 — Source data Fig. 6 [file 44321_2025_319_MOESM7_ESM.zip › Figure 6/Panel B/Permeability masks_time1_time2_used for analysis_RM+rAng1/PC90_10_Top_RM_Ang1_slice18.tif]

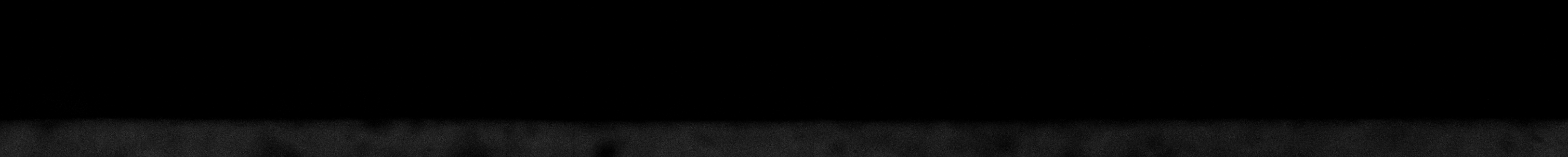

Supplement: Supplementary file 7 — Source data Fig. 6 [file 44321_2025_319_MOESM7_ESM.zip › Figure 6/Panel B/Permeability masks_time1_time2_used for analysis_RM+rAng1/PC90_10_Top_RM_Ang1_slice8.tif]

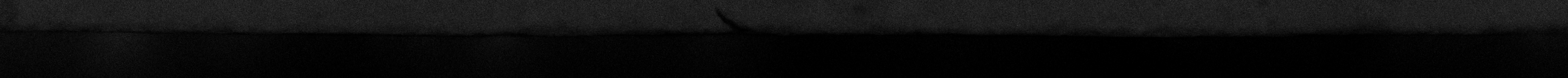

Supplement: Supplementary file 7 — Source data Fig. 6 [file 44321_2025_319_MOESM7_ESM.zip › Figure 6/Panel B/Permeability masks_time1_time2_used for analysis_Serum_free_media_only/PC67_10_Bottom_SM_SF_slice_13.tif]

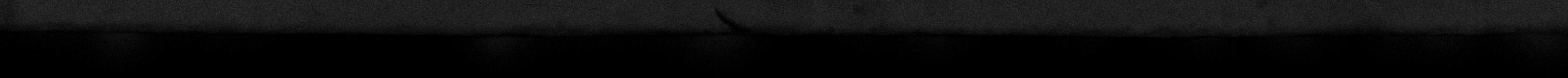

Supplement: Supplementary file 7 — Source data Fig. 6 [file 44321_2025_319_MOESM7_ESM.zip › Figure 6/Panel B/Permeability masks_time1_time2_used for analysis_Serum_free_media_only/PC67_10_Bottom_SM_SF_slice_3.tif]

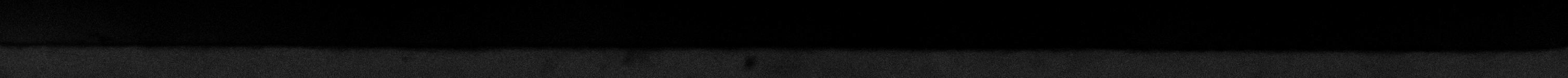

Supplement: Supplementary file 7 — Source data Fig. 6 [file 44321_2025_319_MOESM7_ESM.zip › Figure 6/Panel B/Permeability masks_time1_time2_used for analysis_Serum_free_media_only/PC67_10_Top_SM_SF_slice_13.tif]

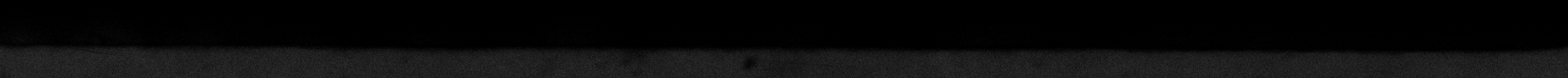

Supplement: Supplementary file 7 — Source data Fig. 6 [file 44321_2025_319_MOESM7_ESM.zip › Figure 6/Panel B/Permeability masks_time1_time2_used for analysis_Serum_free_media_only/PC67_10_Top_SM_SF_slice_3.tif]

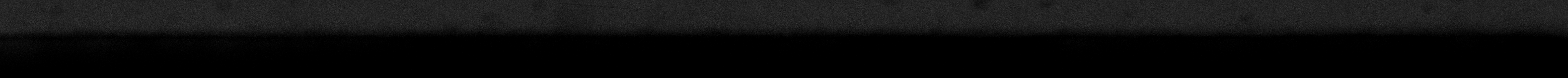

Supplement: Supplementary file 7 — Source data Fig. 6 [file 44321_2025_319_MOESM7_ESM.zip › Figure 6/Panel B/Permeability masks_time1_time2_used for analysis_Serum_free_media_only/PC67_11_Bottom_SM_SF_slice_1.tif]

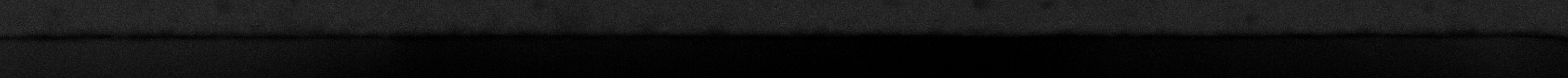

Supplement: Supplementary file 7 — Source data Fig. 6 [file 44321_2025_319_MOESM7_ESM.zip › Figure 6/Panel B/Permeability masks_time1_time2_used for analysis_Serum_free_media_only/PC67_11_Bottom_SM_SF_slice_11.tif]

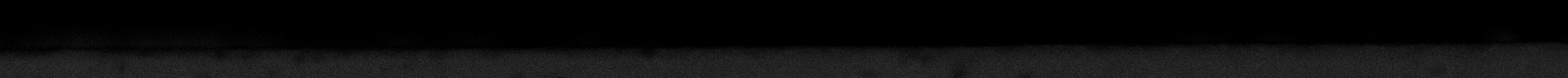

Supplement: Supplementary file 7 — Source data Fig. 6 [file 44321_2025_319_MOESM7_ESM.zip › Figure 6/Panel B/Permeability masks_time1_time2_used for analysis_Serum_free_media_only/PC67_11_Top_SM_SF_slice_1.tif]

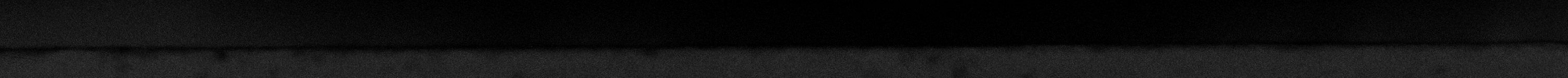

Supplement: Supplementary file 7 — Source data Fig. 6 [file 44321_2025_319_MOESM7_ESM.zip › Figure 6/Panel B/Permeability masks_time1_time2_used for analysis_Serum_free_media_only/PC67_11_Top_SM_SF_slice_11.tif]

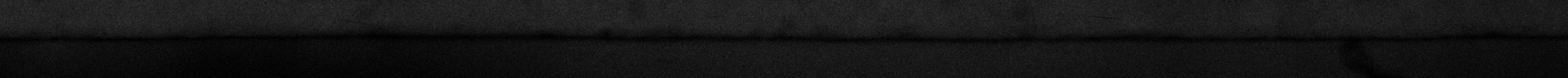

Supplement: Supplementary file 7 — Source data Fig. 6 [file 44321_2025_319_MOESM7_ESM.zip › Figure 6/Panel B/Permeability masks_time1_time2_used for analysis_Serum_free_media_only/PC67_12_Bottom_SM_SF_slice_12.tif]

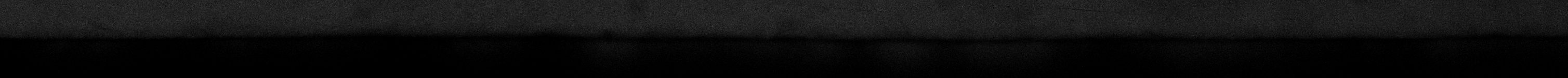

Supplement: Supplementary file 7 — Source data Fig. 6 [file 44321_2025_319_MOESM7_ESM.zip › Figure 6/Panel B/Permeability masks_time1_time2_used for analysis_Serum_free_media_only/PC67_12_Bottom_SM_SF_slice_2.tif]

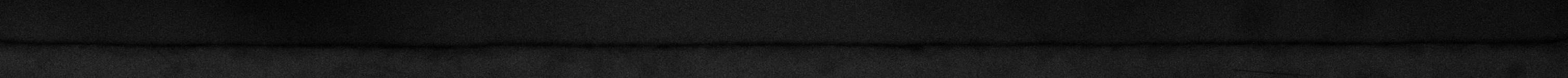

Supplement: Supplementary file 7 — Source data Fig. 6 [file 44321_2025_319_MOESM7_ESM.zip › Figure 6/Panel B/Permeability masks_time1_time2_used for analysis_Serum_free_media_only/PC67_12_Top_SM_SF_slice_12.tif]

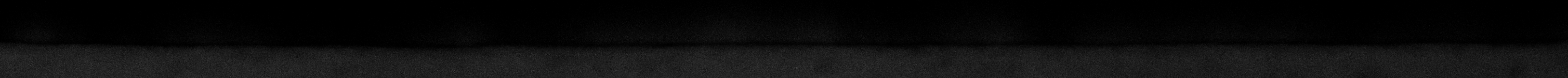

Supplement: Supplementary file 7 — Source data Fig. 6 [file 44321_2025_319_MOESM7_ESM.zip › Figure 6/Panel B/Permeability masks_time1_time2_used for analysis_Serum_free_media_only/PC67_12_Top_SM_SF_slice_2.tif]

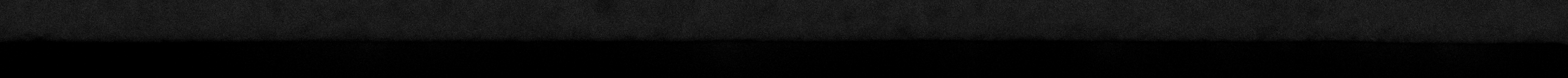

Supplement: Supplementary file 7 — Source data Fig. 6 [file 44321_2025_319_MOESM7_ESM.zip › Figure 6/Panel B/Permeability masks_time1_time2_used for analysis_Serum_free_media_only/PC68_11_Bottom_SM_SF_slice_12.tif]

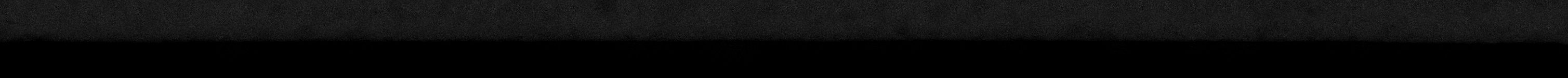

Supplement: Supplementary file 7 — Source data Fig. 6 [file 44321_2025_319_MOESM7_ESM.zip › Figure 6/Panel B/Permeability masks_time1_time2_used for analysis_Serum_free_media_only/PC68_11_Bottom_SM_SF_slice_2.tif]

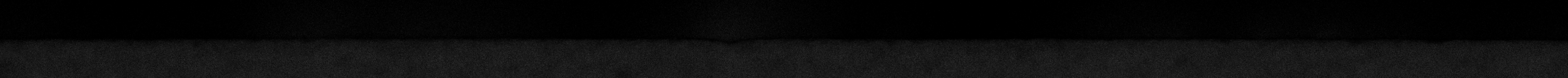

Supplement: Supplementary file 7 — Source data Fig. 6 [file 44321_2025_319_MOESM7_ESM.zip › Figure 6/Panel B/Permeability masks_time1_time2_used for analysis_Serum_free_media_only/PC68_11_Top_SM_SF_slice_12.tif]

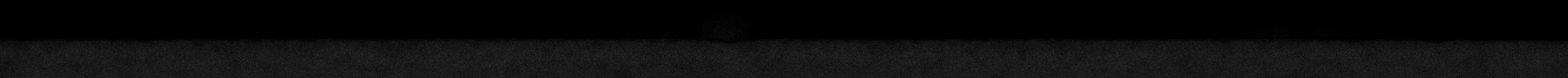

Supplement: Supplementary file 7 — Source data Fig. 6 [file 44321_2025_319_MOESM7_ESM.zip › Figure 6/Panel B/Permeability masks_time1_time2_used for analysis_Serum_free_media_only/PC68_11_Top_SM_SF_slice_2.tif]

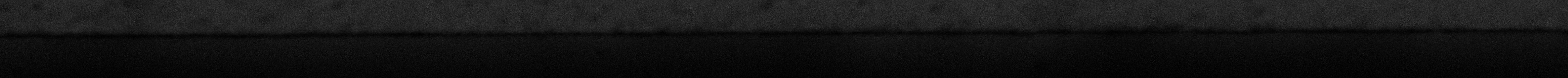

Supplement: Supplementary file 7 — Source data Fig. 6 [file 44321_2025_319_MOESM7_ESM.zip › Figure 6/Panel B/Permeability masks_time1_time2_used for analysis_Serum_free_media_only/PC68_2_Bottom_SM_SF_slice_12.tif]

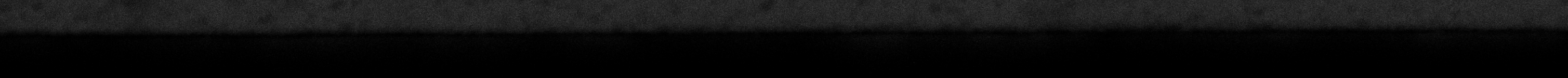

Supplement: Supplementary file 7 — Source data Fig. 6 [file 44321_2025_319_MOESM7_ESM.zip › Figure 6/Panel B/Permeability masks_time1_time2_used for analysis_Serum_free_media_only/PC68_2_Bottom_SM_SF_slice_2.tif]

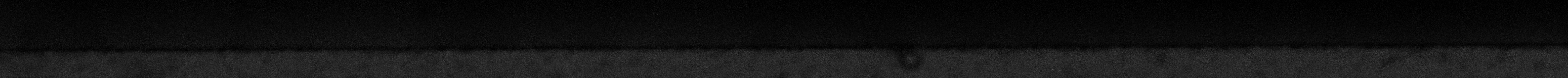

Supplement: Supplementary file 7 — Source data Fig. 6 [file 44321_2025_319_MOESM7_ESM.zip › Figure 6/Panel B/Permeability masks_time1_time2_used for analysis_Serum_free_media_only/PC68_2_Top_SM_SF_slice_12.tif]

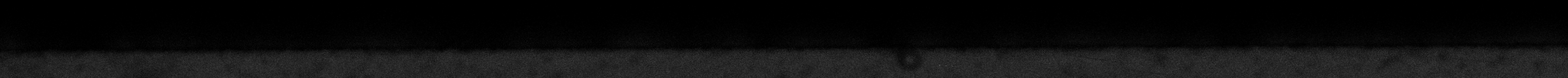

Supplement: Supplementary file 7 — Source data Fig. 6 [file 44321_2025_319_MOESM7_ESM.zip › Figure 6/Panel B/Permeability masks_time1_time2_used for analysis_Serum_free_media_only/PC68_2_Top_SM_SF_slice_2.tif]

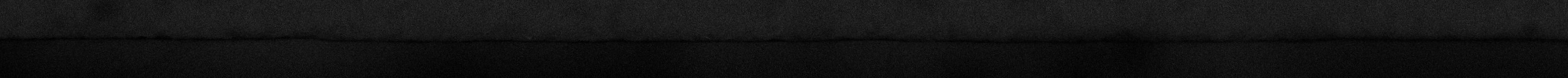

Supplement: Supplementary file 7 — Source data Fig. 6 [file 44321_2025_319_MOESM7_ESM.zip › Figure 6/Panel B/Permeability masks_time1_time2_used for analysis_Serum_free_media_only/PC68_4_Bottom_SM_SF_slice_12.tif]

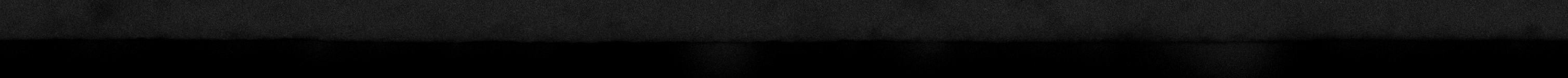

Supplement: Supplementary file 7 — Source data Fig. 6 [file 44321_2025_319_MOESM7_ESM.zip › Figure 6/Panel B/Permeability masks_time1_time2_used for analysis_Serum_free_media_only/PC68_4_Bottom_SM_SF_slice_2.tif]

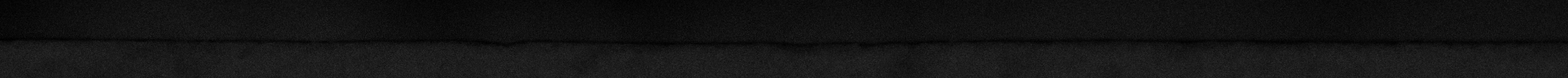

Supplement: Supplementary file 7 — Source data Fig. 6 [file 44321_2025_319_MOESM7_ESM.zip › Figure 6/Panel B/Permeability masks_time1_time2_used for analysis_Serum_free_media_only/PC68_4_Top_SM_SF_slice_12.tif]

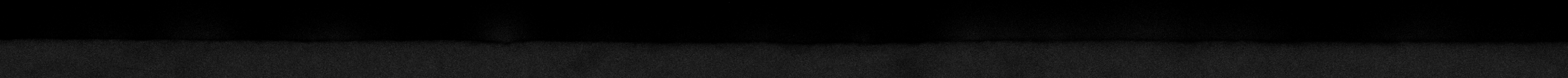

Supplement: Supplementary file 7 — Source data Fig. 6 [file 44321_2025_319_MOESM7_ESM.zip › Figure 6/Panel B/Permeability masks_time1_time2_used for analysis_Serum_free_media_only/PC68_4_Top_SM_SF_slice_2.tif]
