# Supplementary material for: Plasmodium falciparum impairs Ang-1 secretion by pericytes in a 3D brain microvessel model
Source: EMBO Mol Med. 2025 Oct 16;17(11):3110–38. doi: 10.1038/s44321-025-00319-y (PMC12603187; doi:10.1038/s44321-025-00319-y)
Supplement: Supplementary file 8 — Expanded View Figures [file 44321_2025_319_MOESM8_ESM.pdf]

Expanded View Figures

Figure EV1. Characterization of brain-specific endothelial and pericyte marker expression and secretion of angiopoietin–Tie axis components.

(**A**) Immunofluorescence maximum z-projection of a 2D HBMEC monolayer stained for adherens and tight junctional markers: β-catenin, VE-cadherin, ZO-1 (top) and claudin-5 (bottom), and 4′, 6-diamidino-2-phenylindole (DAPI). The merged image includes the adherens junction markers (white), tight junction markers (red) and DAPI labeling (blue). Scale bars: 50 μm. (**B**) Immunofluorescence maximum z-projection of a 2D HBMEC monolayer stained for vWF, brain glucose transporter GLUT-1, CD31, VE-cadherin and DAPI. The merge image includes vWF or GLUT-1 (white), CD31 or VE-cadherin (red) and DAPI labeling (blue). Scale bars: 50 μm. (**C**) Immunofluorescence maximum z-projection of a 2D HBVP monolayer stained for PDGFRβ (top) and NG-2 (bottom), Phalloidin and DAPI. The merge staining includes the pericyte markers (white), phalloidin (red) and DAPI labeling (blue). Scale bars: 50 μm. (**D**) Concentration of secreted angiopoietin-1 (top) and angiopoietin-2 (bottom) in supernatant obtained from either HBVP-HBMEC co-culture or HBMEC-only monolayers grown in a transwell model. Data is presented as mean $+/-$ standard deviation ($n = 3$ independent experiments for angiopoetin-1 measurement and 4 for angiopoietin-2 measurement, Mann–Whitney *U* test).

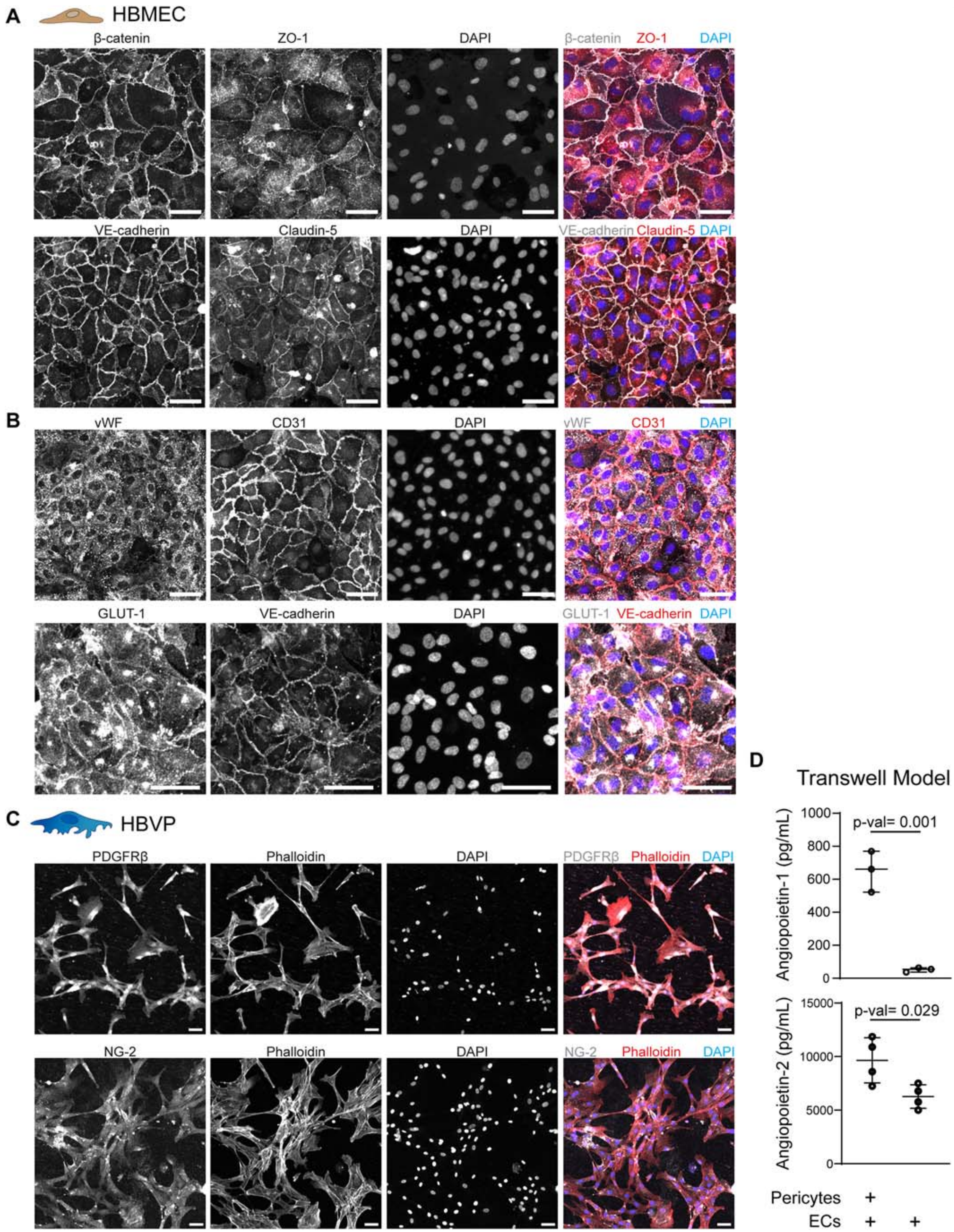
A
HBMEC
β-catenin
ZO-1
DAPI
β-catenin ZO-1 DAPI
VE-cadherin
Claudin-5
DAPI
VE-cadherin Claudin-5 DAPI
B
vWF
CD31
DAPI
vWF CD31 DAPI
GLUT-1
VE-cadherin
DAPI
GLUT-1 VE-cadherin DAPI
C
HBVP
PDGFRβ
Phalloidin
DAPI
PDGFRβ Phalloidin DAPI
NG-2
Phalloidin
DAPI
NG-2 Phalloidin DAPI
D
Transwell Model
Angiopoietin-1 (pg/mL)
p-val= 0.001
1000
800
600
400
200
0
Angiopoietin-2 (pg/mL)
p-val= 0.029
15000
10000
5000
0
Pericytes +
ECs + +

 © The Author(s)

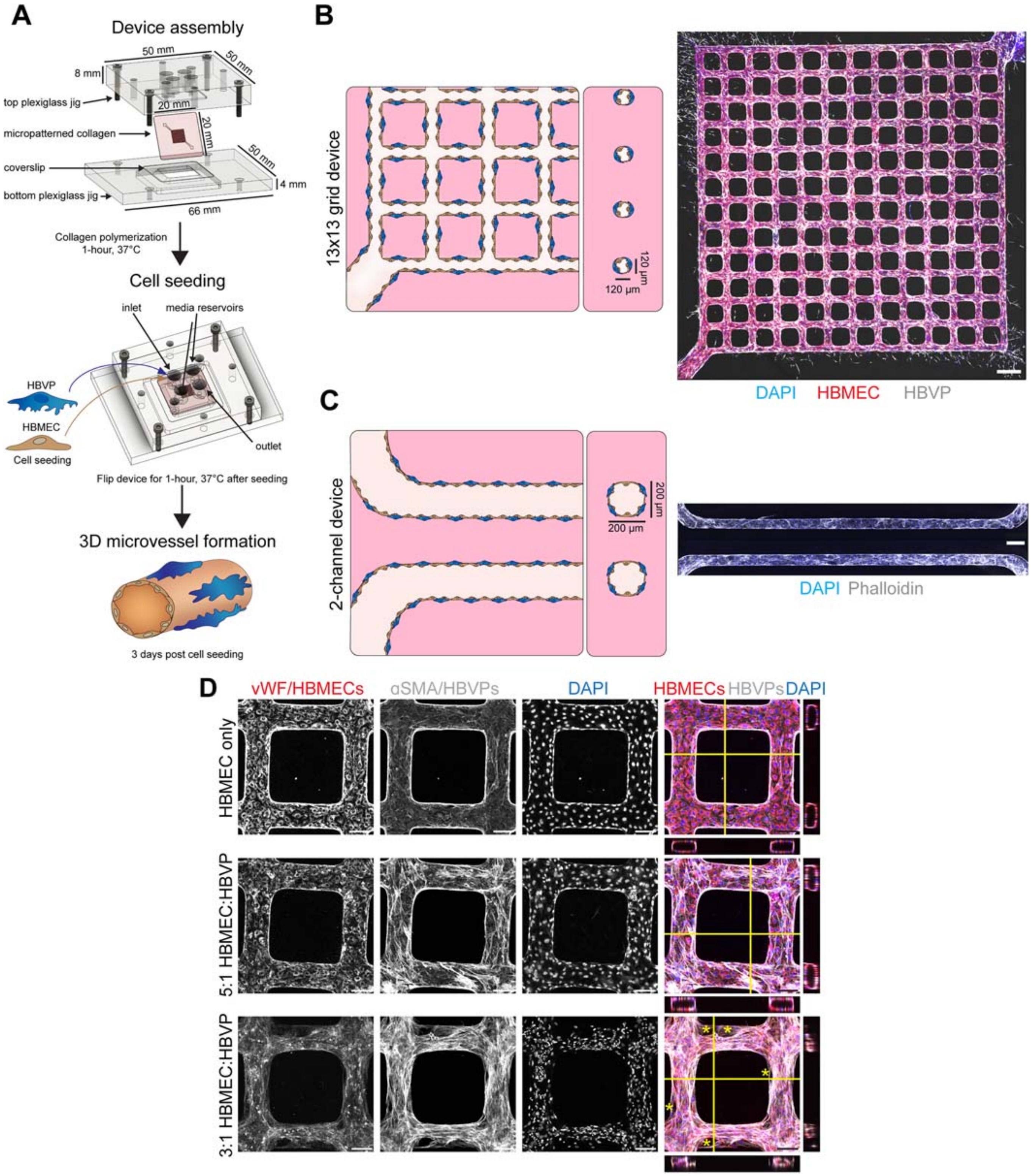
A
Device assembly
50 mm
50 mm
8 mm
top plexiglass jig
20 mm
20 mm
micropatterned collagen
50 mm
coverslip
4 mm
bottom plexiglass jig
66 mm
Collagen polymerization
1-hour, 37°C
Cell seeding
inlet
media reservoirs
HBVP
HBMEC
Cell seeding
outlet
Flip device for 1-hour, 37°C after seeding
3D microvessel formation
3 days post cell seeding
B
13x13 grid device
120 µm
120 µm
DAPI HBMEC HBVP
C
2-channel device
200 µm
200 µm
DAPI Phalloidin
D
vWF/HBMECs
αSMA/HBVPs
DAPI
HBMECs HBVPs DAPI
HBMEC only
5:1 HBMEC:HBVP
3:1 HBMEC:HBVP

◀ **Figure EV2. Fabrication of the 3D brain microvessel model.**

(**A**) Schematic representation of the protocol to fabricate the 3D brain microvessel model including device assembly, cell seeding and 3D microvessel formation. (**B**) Schematic overview and cross section of the 13 × 13 grid microvessel network (left). IFA maximum z-projection of the complete grid microvessel network stained for vWF for endothelial cells (red), mCherry for pericytes (white) and DAPI (blue). Scale bar: 500 µm. (**C**) Schematic overview and cross section of the 2-channel microvessel network (left). IFA maximum z-projection of the complete grid microvessel network stained for phalloidin (white) and DAPI (blue). Scale bar: 200 µm. (**D**) Immunofluorescence assay (IFA) maximum z-projection of microvessels fabricated using HBMEC-only, a 3:1 or 5:1 HBMEC to HBVP ratio labeled with vWF for HBMEC (red), αSMA for HBVP (white) and DAPI (blue). Orthogonal views display the presence or absence of a perfusable microvessel lumen and yellow asterisks represent regions of microvessel detachment. Scale bar: 100 µm.

 © The Author(s)

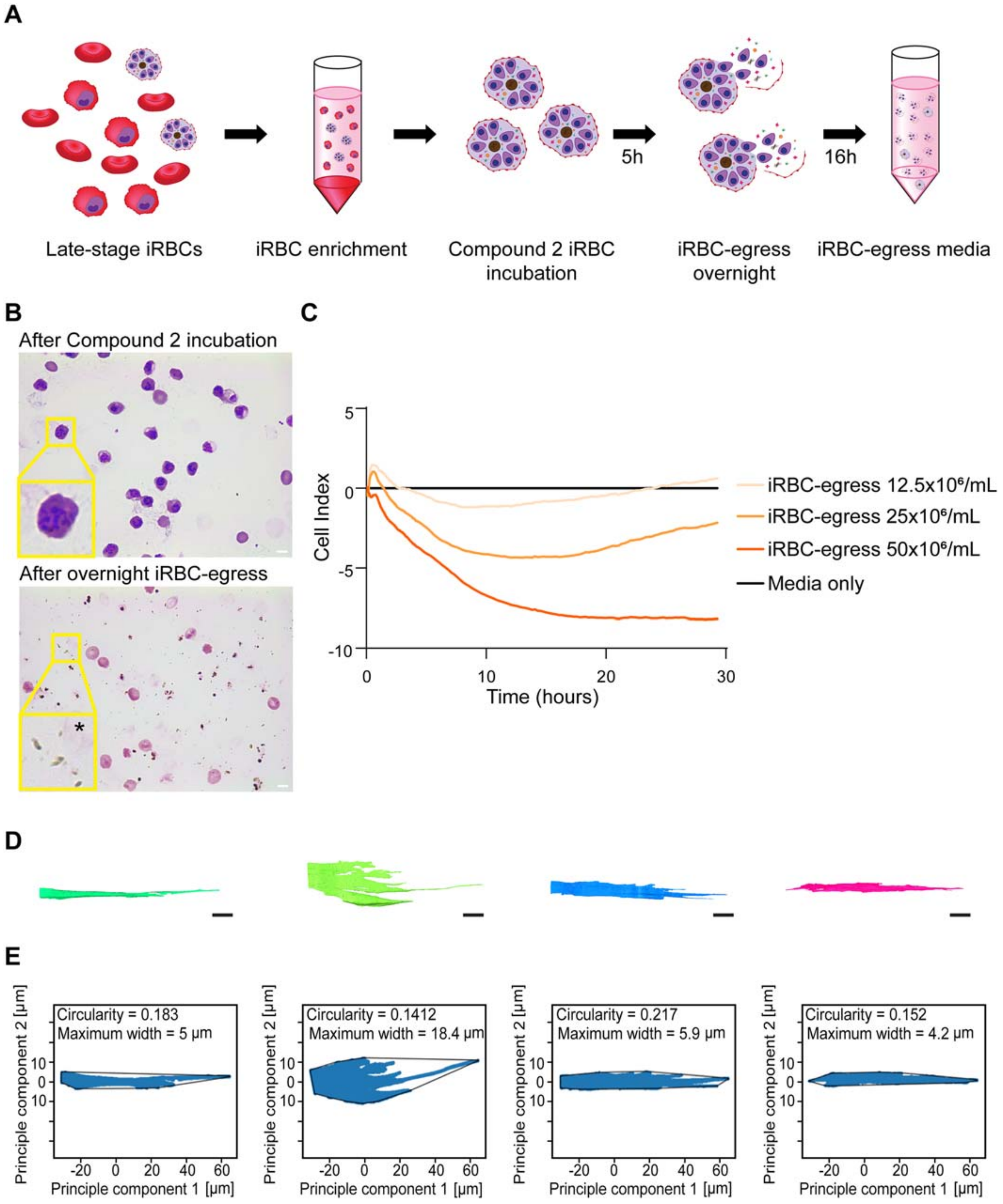
A
Late-stage iRBCs
iRBC enrichment
Compound 2 iRBC incubation
5h
iRBC-egress overnight
16h
iRBC-egress media
B
After Compound 2 incubation
After overnight iRBC-egress
*
C
Cell Index
Time (hours)
iRBC-egress 12.5x10⁶/mL
iRBC-egress 25x10⁶/mL
iRBC-egress 50x10⁶/mL
Media only
D
E
Circularity = 0.183
Maximum width = 5 µm
Circularity = 0.1412
Maximum width = 18.4 µm
Circularity = 0.217
Maximum width = 5.9 µm
Circularity = 0.152
Maximum width = 4.2 µm
Principle component 1 [µm]
Principle component 2 [µm]

Figure EV3. Generation of endothelial barrier disruptive iRBC-egress media and a pipeline for analysis of pericyte morphological features.

(**A**) Schematic representation of the protocol to make iRBC-egress media. In short, late-stage *P. falciparum*-iRBC are purified by a gelaspan gradient separation and then incubated for 5 h with compound-2 to synchronize them at the point of egress. Compound-2 is removed and the *P. falciparum*-iRBC are resuspended in vascular growth media and left overnight on a shaker at 50 rpm to egress. (**B**) A thin smear of tightly synchronized schizonts before the removal of Compound-2 (yellow inset highlights a ROI with a schizont-stage iRBC) or the resultant iRBC-egress media before centrifugation (yellow inset highlights a ROI with free hemozoin particles next to an iRBC ghost, denoted with an asterisk) stained with Giemsa. Scale bars: 5 µm. (**C**) Representative recording data of xCELLigence measurements on 2D HBMEC monolayers in the absence of HBVP taken after addition of iRBC-egress media at different concentrations. Data is normalized to the media-only control. (**D**) The analysis pipeline begins with the extraction of 3D segmented pericyte meshes. Shown here are four representative pericytes. Scale bars: 10 µm. (**E**) Pericyte geometry is flattened into two principal dimensions using PCA by a Python-based image analysis pipeline. The 2D geometry's shape is then determined using convex hull analysis. To describe pericyte morphology, pericyte cell borders are analyzed for circularity, calculated as 4π*Area/Perimeter^2, where a value of 1 indicates a perfect circle and values approaching 0 indicate increasingly elongated shapes, and maximum width.

 © The Author(s)

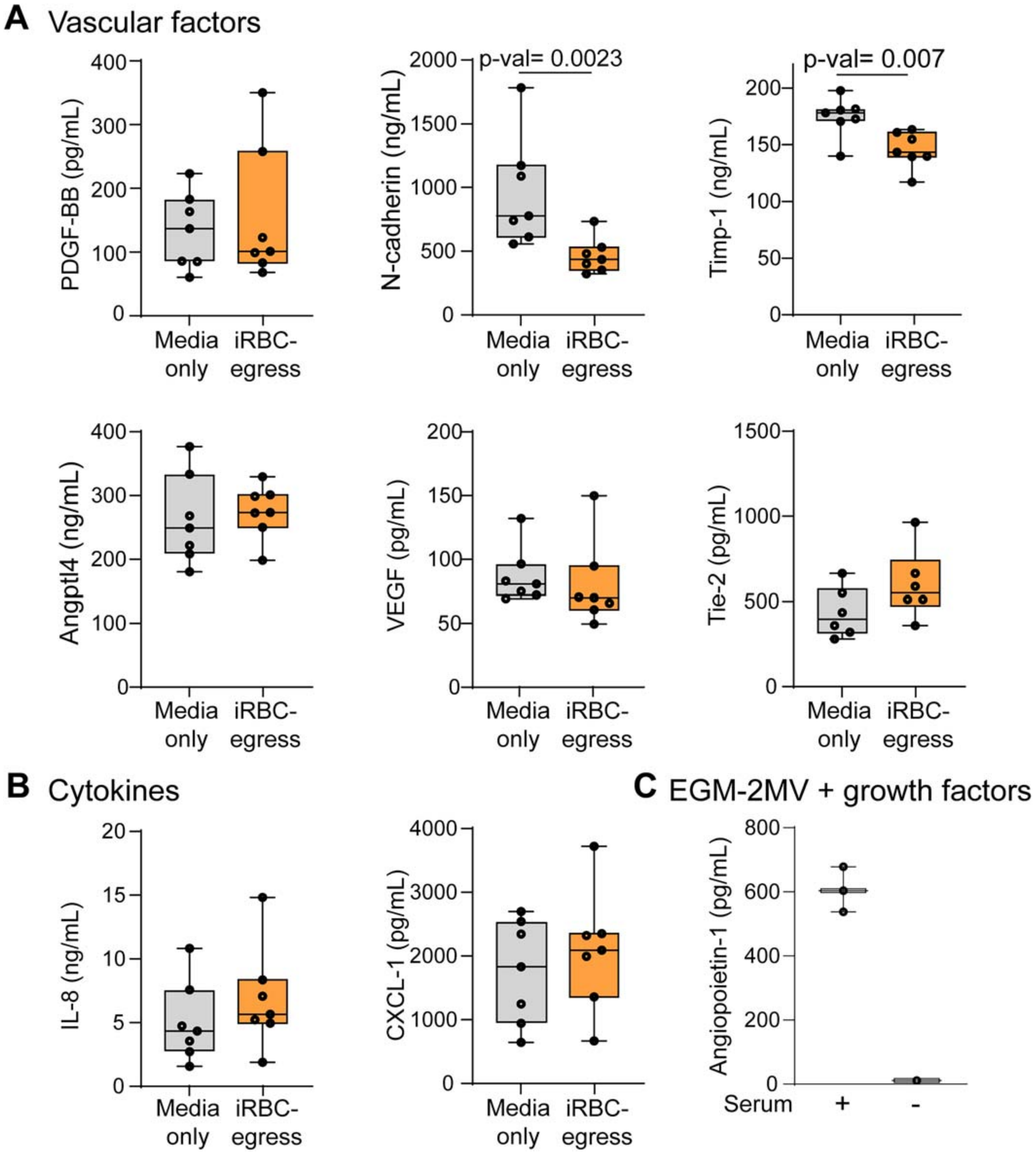

Figure EV4. iRBC egress products cause alteration of endothelial cell-pericyte interaction markers.

(**A**) Concentrations of vascular factors PDGF-BB, N-cadherin, Timp-1, Angptl4, VEGF and Tie-2 measured by Luminex from 3D brain microvessels supernatants treated with media-only or iRBC-egress media for 18-h. Box and whisker plots display the median, 25th and 75th percentiles and the minimum and maximum data points. ($n = 7$ supernatants pooled from 2–3 devices each). (**B**) Concentrations of released cytokines IL-8 and CXCL-1 measured by Luminex from 3D brain microvessels supernatants treated with media-only or iRBC-egress media for 18-h. Box and whisker plots display the median, 25th and 75th percentiles and the minimum and maximum data points. ($n = 7$ supernatants pooled from 2-3 devices each). (**C**) Concentrations of angiopoietin-1 measured by Luminex from freshly made growth factor-containing EGM-2MV with or without serum. Box and whisker plots display the median, 25th and 75th percentiles and the minimum and maximum data points. ($n = 3$, where angiopoietin-1 in serum-free vascular media was below the limit of detection). Data information: Statistical significance is analyzed by Mann–Whitney *U* test (**A**, **B**).

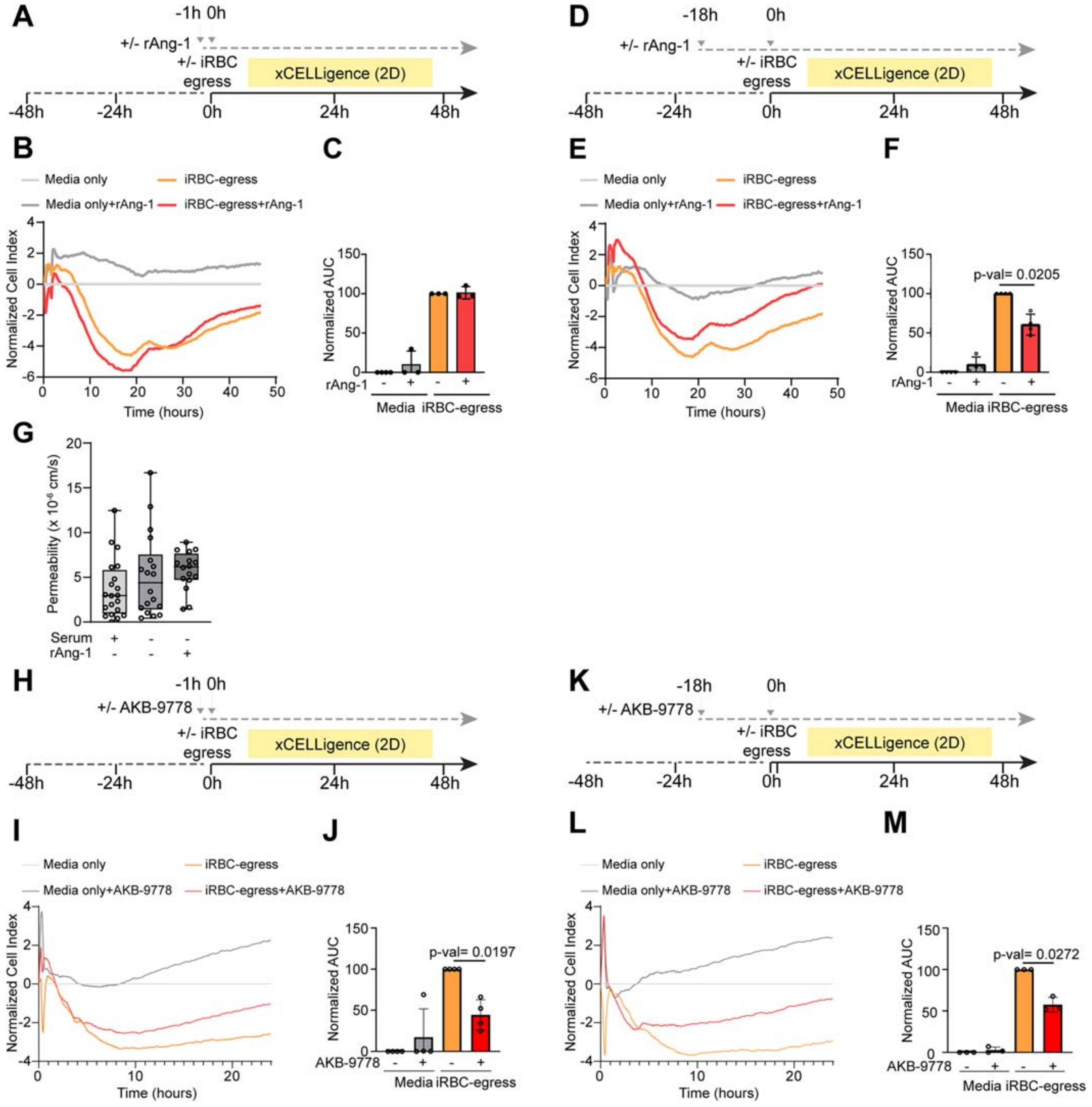
A
-1h 0h
+/- rAng-1
+/- iRBC egress
xCELLigence (2D)
-48h -24h 0h 24h 48h
B
Media only
iRBC-egress
Media only+rAng-1
iRBC-egress+rAng-1
Normalized Cell Index
Time (hours)
C
Normalized AUC
rAng-1
Media iRBC-egress
D
-18h 0h
+/- rAng-1
+/- iRBC egress
xCELLigence (2D)
E
F
p-val= 0.0205
G
Permeability (x 10⁻⁶ cm/s)
Serum + - -
rAng-1 - - +
H
-1h 0h
+/- AKB-9778
+/- iRBC egress
xCELLigence (2D)
I
Media only+AKB-9778
iRBC-egress+AKB-9778
J
p-val= 0.0197
AKB-9778
K
-18h 0h
+/- AKB-9778
L
M
p-val= 0.0272

Figure EV5. Recombinant Ang-1 or AKB-9778 pre-treatment partially protects against 2D endothelial monolayer permeability increase induced by iRBC egress products.

(**A**) Experimental outline of short-term (1-h) incubation with rAng-1 in a 2D HBMEC monolayer in the absence of HBVP. (**B**) Representative recording data of xCELLigence measurements taken after a 1-h +/-rAng-1 pre-treatment followed by +/- iRBC-egress media addition. All conditions were normalized to the media-only control. (**C**) Area under the curve analysis with iRBC-egress media normalized as 100 ($n = 3$ independent experiments run in triplicate). (**D**) Experimental outline of long-term (18-h) incubation with rAng-1 in a 2D HBMEC monolayer in the absence of HBVP. (**E**) Representative recording data of xCELLigence measurements taken after an 18-h +/− rAng-1 pre-treatment followed by +/− iRBC-egress media addition. All conditions were normalized to the media-only control. (**F**) Area under the curve analysis with iRBC-egress media normalized as 100. ($n = 4$ independent experiments run in triplicate). (**G**) Apparent permeability of 70 kDa FITC-dextran in microvessels pre-treated with serum containing media, serum-free media or serum-free media+rAng-1 ($n = 10$ individual devices for serum containing media incubation, 9 for serum-free media incubation and 8 for serum-free media+rAng-1 incubation). (**H**) Experimental outline of short-term (1-h) incubation with AKB-9778 in a 2D HBMEC monolayer in the absence of HBVP. (**I**) Representative recording data of xCELLigence measurements taken after a 1-h +/−AKB-9778 pre-treatment followed by +/− iRBC-egress media addition. All conditions were normalized to the media-only control. (**J**) Area under the curve analysis with iRBC-egress media normalized as 100 (n= of 4 independent experiments run in triplicate). (**K**) Experimental outline of long-term (18-h) incubation with AKB-9778 in a 2D HBMEC monolayer in the absence of HBVP.
(**L**) Representative recording data of xCELLigence measurements taken after an 18-h +/− rAng-1 pre-treatment followed by +/− iRBC-egress media addition. All conditions were normalized to the media-only control. (**M**) Area under the curve analysis with iRBC-egress media normalized as 100. ($n = 3$ independent experiments run in triplicate). Data information: In (**C**, **F**, **J**, **M**), data are presented as bar charts displaying the mean +/- standard deviation (Repeated measures one-way ANOVA test with Dunnett's multiple comparisons test. In (**G**), box and whisker plots display the median, 25th and 75th percentiles and the minimum and maximum data points (Kruskal–Wallis test corrected for multiple comparisons using the Benjamini, Krieger and Yekutieli method).

© The Author(s)
